# Real-time estimation of pathogen transmission dynamics from wastewater

**DOI:** 10.1038/s41467-026-75380-3

**Published:** 2026-07-11

**Authors:** Adrian Lison, Rachel E. McLeod, Jana S. Huisman, James D. Munday, Christoph Ort, Timothy R. Julian, Tanja Stadler

**Affiliations:** 1 ETH Zurich, Department of Biosystems Science and Engineering, Zurich, Switzerland; 2 SIB Swiss Institute of Bioinformatics, Lausanne, Switzerland; 3 Eawag, Swiss Federal Institute of Aquatic Science and Technology, Dubendorf, Switzerland; 4 Physics of Living Systems, Massachusetts Institute of Technology, Cambridge, MA USA; 5 Swiss Tropical and Public Health Institute, Allschwil, Switzerland; 6 University of Basel, Basel, Switzerland

**Keywords:** Water microbiology, Population dynamics, Statistical methods, Statistics, Infectious diseases

## Abstract

Wastewater monitoring proved effective for tracking SARS-CoV-2 transmission during the COVID-19 pandemic. However, estimating transmission parameters for other pathogens remains difficult due to lower concentrations in sewage, uncertain shedding kinetics, and limited clinical validation data. Here we present EpiSewer, a Bayesian semi-mechanistic wastewater model that jointly accounts for infection dynamics, pathogen shedding, and measurement noise, including outliers and non-detects. This enables direct inference of the effective reproduction number (*R*_*t*_) and epidemic growth rate (*r*_*t*_) from raw concentration and flow data, eliminating the need for prior smoothing, imputation, or outlier removal. We assessed EpiSewer across three seasons of multi-pathogen wastewater surveillance (Nov 2022 – May 2025) at 6–14 treatment plants in Switzerland, tracking SARS-CoV-2, influenza A virus (IAV), and respiratory syncytial virus (RSV) transmission in real time. *R*_*t*_ estimates were consistent and robust to measurement noise, even with IAV and RSV concentrations 10–50 times lower than SARS-CoV-2. The model provided well-calibrated fourteen-day concentration forecasts, with minimal bias across epidemic phases. Under reduced sampling frequencies, EpiSewer maintained unbiased forecasts while accurately reflecting uncertainty. Our approach enables robust inference of transmission dynamics for lower-abundance pathogens with limited clinical surveillance, using only a few wastewater samples per week.

## Abstract (web summary)

Tracking infectious disease transmission from wastewater is hindered by noise, sampling variation, and low pathogen concentrations. Here, the authors develop EpiSewer, a Bayesian model to estimate reproduction numbers and growth rates in real time.

## Introduction

Wastewater-based infectious disease surveillance has gained significant traction during the COVID-19 pandemic as an effective tool for monitoring community-wide transmission dynamics^1^. When infected individuals shed genetic material of a pathogen into a municipal sewage system, the pathogen concentration in wastewater samples, determined using polymerase chain reaction (PCR) techniques, provides a signal of the trend in infections over time^2,3^. The successful application of wastewater surveillance to SARS-CoV-2 has catalyzed the development of assays for other pathogens, including influenza A and B virus (IAV/IBV)^4,5^, respiratory syncytial virus (RSV)^6,7^, monkeypox virus^8^, and human adenovirus^9^. Recent advances, such as digital PCR multiplexing, now allow the simultaneous detection of multiple pathogens in a single assay^10,11^, enabling cost-effective, multi-target surveillance through shared sampling and laboratory processes^12^.

As wastewater surveillance programs expand to include a broader range of pathogens, wastewater-based estimates of transmission parameters can provide valuable insights into the spread of infectious diseases, supporting public health policy and health sector preparedness. For example, estimates of the effective reproduction number *R*_*t*_, indicating the average number of secondary infections caused per infected individual^13^, can quantify trends and regional differences in the circulation of common viral infections and inform healthcare demand planning^14,15^. Similarly, estimates of *R*_*t*_ and the epidemic growth rate can be used to identify exponential growth trends during outbreaks and support the implementation and evaluation of public health measures^16–18^. These applications of wastewater monitoring are especially promising for emerging and non-notifiable diseases, where clinical surveillance is often sparse or non-existent^19^.

To date, wastewater-based *R*_*t*_ and growth rates have been primarily estimated for SARS-CoV-2^20–28^, which is found in wastewater at high concentrations and for which clinical surveillance data were widely available for model calibration and validation during the COVID-19 pandemic. Increasingly, other respiratory viruses such as IAV, IBV, and RSV, are being monitored in sewage across several countries^4–7,10^. However, routine estimation of transmission dynamics from wastewater remains challenging due to several factors.

First, for many pathogens, measured gene concentrations are substantially lower than those of SARS-CoV-2, reducing the signal-to-noise ratio in wastewater data^7^. Specifically, PCR-based gene concentration measurements are subject to concentration-dependent measurement noise and non-detects^29,30^, which can frequently occur for pathogens detected at lower concentrations. These statistical properties are seldom reflected in epidemiological models, which typically assume – explicitly or implicitly – that wastewater measurements have a similar error structure as case counts^3,21^ or follow generic distributions such as the normal, log-normal or gamma distribution^25–27^. This also means that non-detects, i.e., zero measurements, must be imputed or removed from the data^26^.

In addition, wastewater time series often contain gaps from unsampled days, typically due to cost constraints in monitoring programs^31,32^. To interpolate and smooth these data, non-parametric methods, such as moving averages^20^, LOESS^21^, or smoothing splines^23^, are commonly applied. These methods rely on the calibration of hyperparameters such as window sizes, weights, or knot positions, which can substantially affect the inferred transmission dynamics. Calibration is particularly challenging for pathogens with limited or no supporting clinical data, as the variability of wastewater measurements depends on numerous factors, including the pathogen’s shedding kinetics, sampling frequency, and laboratory methods^33^. Moreover, uncertainty from non-parametric smoothing is generally difficult to propagate to the subsequent modeling.

Finally, transmission parameter estimates are influenced by the specific shedding kinetics of each pathogen. Because fecal shedding is distributed over time after infection^34,35^, current wastewater concentrations reflect the aggregate, partial shedding of individuals infected at different times in the recent past. Consequently, wastewater data provide a temporally blurred signal of community transmission dynamics^13^, imposing a natural limit on the precision with which *R*_*t*_ or growth rates can be estimated near the present, potentially leading to overconfident real-time estimates if not accounted for^20,21,36^.

However, the shedding profiles of most pathogens remain poorly characterized^37,38^, and shedding profile uncertainty is typically neglected when estimating transmission parameters from wastewater^24,27^.

Collectively, these factors reduce the reliability of wastewater-based estimation of transmission dynamics. Moreover, because wastewater models are rarely evaluated in real time – partly due to the lack of ground truth for *R*_*t*_ or growth rates – errors in model specification can go unnoticed, for example, when surveillance programs reduce sampling frequency to cut costs^31^.

To address these challenges, we developed EpiSewer, a Bayesian semi-mechanistic wastewater model that explicitly represents the underlying infection, shedding, and measurement processes in wastewater surveillance. This enables routine transmission monitoring of multiple pathogens by estimating *R*_*t*_ and growth rates from raw, noisy concentration measurement data without prior smoothing, imputation, or outlier detection. Through epidemiologically interpretable parameters, the model can be adapted to different pathogens, taking into account limited knowledge of shedding characteristics through uncertain priors. Moreover, our generative modeling approach naturally provides probabilistic short-term forecasts of concentration measurements, enabling performance evaluation in near real time during ongoing surveillance.

We have used EpiSewer during three years of multi-pathogen wastewater monitoring in Switzerland to produce real-time estimates of *R*_*t*_, informing Swiss public health officials via a public dashboard at https://wise.ethz.ch with data from currently 10 wastewater treatment plants (WWTPs). Here, we demonstrate the capabilities of our approach in quantifying the transmission dynamics of SARS-CoV-2, influenza A virus (IAV), and respiratory syncytial virus (RSV) across the last three seasonal waves. We evaluate the real-time performance of wastewater-based *R*_*t*_ estimates and pathogen concentration forecasts and test the model’s ability to handle non-daily sampling and outliers in the measurement data. Our findings highlight the strength of generative modeling to robustly infer transmission dynamics of pathogens from sparse and noisy wastewater data.

## Results

### Wastewater model

**Fig. 1:**
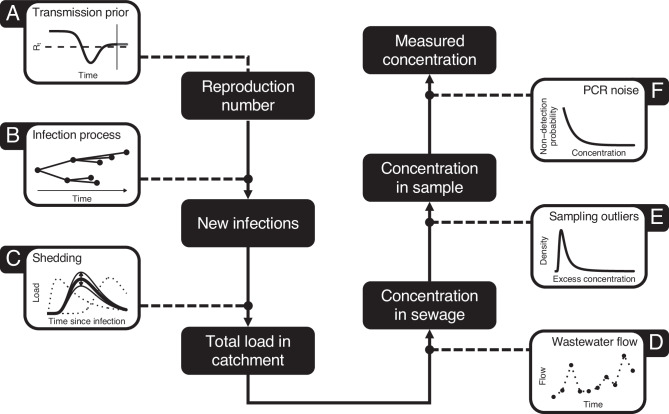
Visual summary of the EpiSewer model. **A** Population-level transmission dynamics are modeled via smooth changes in the effective reproduction number *R*_*t*_. **B** New infections are generated through an age-dependent branching process, represented by a stochastic renewal model. **C** Infected individuals shed genetic material into the wastewater, modeled via an uncertain shedding profile with individual-level variation in shedding intensity. **D** The concentration of genetic material in the sewage depends on the dilution by wastewater flow within the catchment area. **E** Concentrations in wastewater samples are assumed to be representative of sewage concentrations, with occasional outliers following an extreme value distribution. **F** PCR-based concentration measurements are modeled with concentration-dependent measurement noise and non-detects. We here assume a digital PCR approach.

EpiSewer is a Bayesian model describing the time evolution of pathogen concentrations in wastewater (Fig. 1). In this model, new infections are generated through a time-discrete, stochastic renewal process with a smoothly time-varying effective reproduction number *R*_*t*_ (Fig. 1A, B). After infection, individuals shed genetic material of the pathogen into the wastewater over a certain number of days according to a shedding profile (Fig. 1C). The relative intensity of shedding on each day after infection follows a discretized, parametric distribution with uncertain mean and variance, and the total shedding load can vary across individuals. We further assume that genetic material is well mixed in sewage and diluted in proportion to the daily measured wastewater flow within the catchment (Fig. 1D). Samples taken at a WWTP are assumed to be representative of the daily wastewater concentration, with the exception of occasional high outlier concentrations, e.g., due to incomplete mixing, which are modeled using an extreme value distribution (Fig. 1E). Finally, pathogen concentrations in the sample are quantified using PCR, where measurements are subject to concentration-dependent noise and false-negative non-detects, i.e., a measured concentration of zero despite the presence of target nucleic acid in the sample (Fig. 1F). We here model a digital PCR approach, in which sample extracts are independently amplified and tested across a large number of chambers or droplets. However, other techniques, such as quantitative PCR, can be incorporated in a similar manner.

Using Markov Chain Monte Carlo sampling, EpiSewer is fitted directly to a time series of measured pathogen concentrations to produce Bayesian estimates of the effective reproduction number *R*_*t*_, epidemic growth rate *r*_*t*_, and other latent parameters useful for model inspection and misspecification checking, e.g., infection numbers (implied by the assumed shedding load per infection) and total pathogen loads. By projecting the current effective reproduction number up to 14 days ahead, we also obtain a posterior predictive distribution of past and future concentration measurements. This enables inspection of the model fit on current data as well as retrospective model validation by assessing the accuracy of short-term concentration forecasts when new data become available.

### Handling of non-detects and outliers in concentration data

**Fig. 2:**
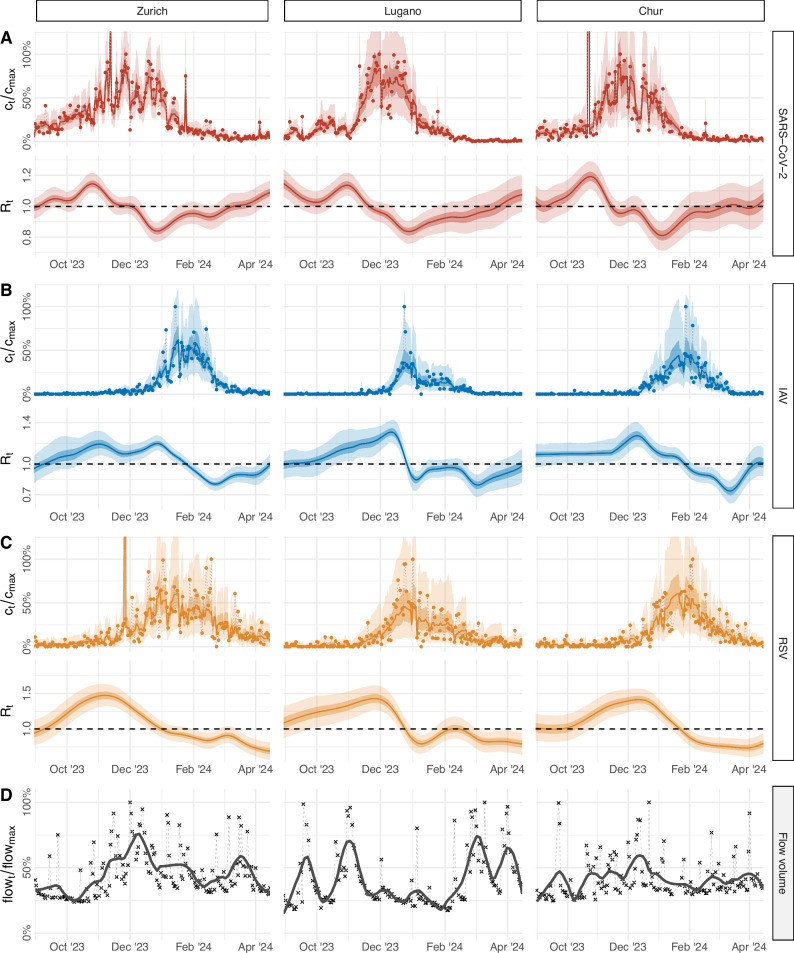
Viral transmission dynamics in Switzerland quantified from wastewater at treatment plants in Zurich, Chur, and Lugano during the 2023/24 winter season. **A**–**C** Relative pathogen concentrations (top) and estimates of the effective reproduction number *R*_*t*_ (bottom) for SARS-CoV-2 (red), influenza A virus (IAV; blue), and respiratory syncytial virus (RSV; orange) based on longitudinal samples from a large (Zurich, 471000 persons), medium-sized (Lugano, 124000 persons), and small (Chur, 55000 persons) wastewater catchment in Switzerland during the winter season 2023/24. Dots show measured pathogen concentrations. Lines indicate posterior medians; shaded bands represent 50% (dark) and 95% (light) credible intervals of predicted concentrations and estimated *R*_*t*_, respectively. Concentrations were normalized by the maximum observed non-outlier concentration (${c}_{\max }$). **D** Relative daily wastewater flow at the sampled treatment plant of each catchment. Lines show a smooth trend estimated using LOESS with a 4-week window. Flows were normalized by the maximum observed flow (${{\mbox{flow}}}_{\max }$). Source data are provided as a Source Data file.

We fitted the model to concentration measurements of SARS-CoV-2, IAV, and RSV from a multi-year wastewater surveillance program in Switzerland that comprised 6 WWTPs during the winter season 2022/23, 14 WWTPs during the winter season 2023/24, and 10 WWTPs during the winter season 2024/25^5,39^. For each WWTP, pathogen concentrations were quantified using digital droplet PCR from an average of 5 samples per week until 2025, then from 4 samples per week. Figure 2A–C shows concentrations measured during the winter season 2023/24 in Switzerland, which featured clear seasonal waves of all three pathogens, for the catchments of Zurich (471000 persons), Lugano (124000 persons), and Chur (55000 persons). Results for further catchments and the winter seasons 2022/23 and 2024/25 are shown in Supplementary Information F.

For each pathogen, gene concentrations were similar across catchments. However, absolute concentrations of IAV and RSV were considerably lower than those of SARS-CoV-2. Across the 14 WWTPs sampled, the maximum measured concentration (excluding outliers) ranged between 914–2973 gc/mL for SARS-CoV-2, but only 43–302 gc/mL for IAV and 18–104 gc/mL for RSV. The lower concentrations resulted in a large percentage of non-detects at the start of the winter season (46–83 % of IAV and 19–42 % of RSV measurements before December 31, 2023). Using our dPCR-specific measurement model, we were able to include these observations without imputation or censoring.

**Fig. 3:**
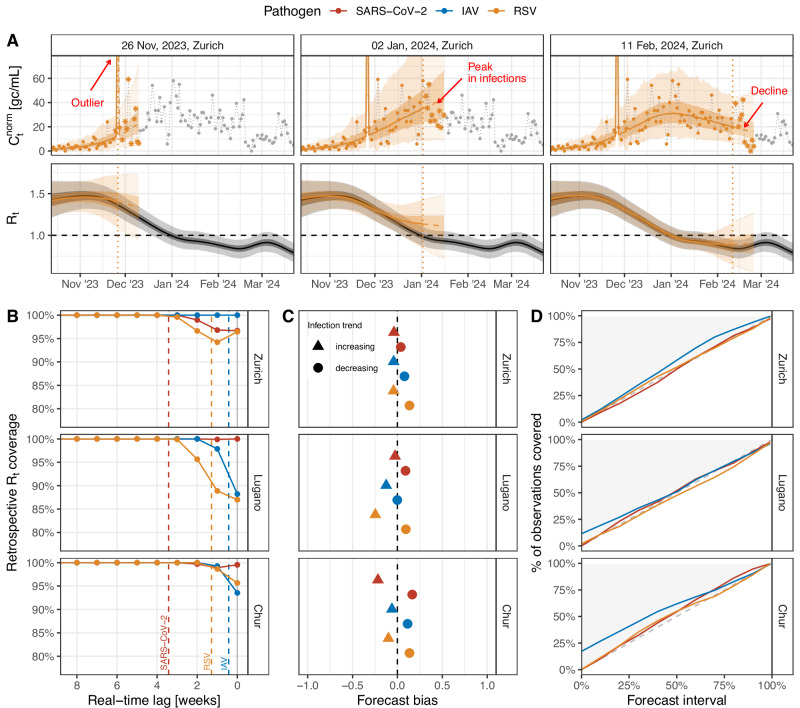
Real-time performance of wastewater-based effective reproduction number (*R*_*t*_) estimates and concentration forecasts. We evaluated real-time *R*_*t*_ estimates and 1–14-day-ahead forecasts of concentration measurements for the catchments of Zurich, Lugano, and Chur, Switzerland, during the 2023/24 seasonal wave of SARS-CoV-2, influenza A virus (IAV), and respiratory syncytial virus (RSV). **A** Example real-time estimates for RSV in Zurich on three estimation dates (vertical dotted lines): immediately after a high outlier measurement, at the peak of the seasonal wave, and during the declining phase of infections. Top panels show flow-adjusted (i.e., rescaled to median flow) forecasts and observed concentrations (stars). Bottom panels show real-time estimates and 14-day-ahead projections of *R*_*t*_, along with retrospective *R*_*t*_ (in gray). Lines indicate posterior medians; shaded bands represent 50% (dark) and 95% (light) credible intervals. **B** Consistency of real-time *R*_*t*_ (estimated on each day with new data) with retrospective *R*_*t*_ (estimated at the end of the seasonal wave). For each real-time *R*_*t*_ estimate, we computed the retrospective coverage, i.e., the percentage of days on which the 95% credible interval (CrI) contained the retrospective median *R*_*t*_. Dots show the coverage stratified by lags of 0–8 weeks from the date of estimation. Vertical dashed lines show the 90% quantile of the temporal shedding profile of each pathogen. **C** Bias of concentration measurement forecasts, stratified by increasing vs. decreasing infection trends. Scores range from -1 (maximum underprediction) to 1 (maximum overprediction); 0 indicates no bias. **D** Calibration of concentration measurement forecasts. The diagonal line indicates perfect calibration, where predictive intervals match their nominal coverage. Source data are provided as a Source Data file.

We also observed occasional extreme spikes in measured concentrations across all monitored seasons. By modeling excess sample concentrations via an extreme value distribution, EpiSewer can automatically identify individual outlier observations. For example, during the winter season 2023/24, we encountered outliers for SARS-CoV-2 in Chur (Oct 22, 2023) and for RSV in Zurich (Nov 26, 2023), falling into the 99.89% and 99.87% quantiles of the extreme value distribution (Fig. 2). This allowed us to fit our model without prior outlier detection. Indeed, excluding detected outliers before fitting the model produced practically identical *R*_*t*_ estimates, indicating that they had no significant impact on the estimated transmission dynamics (Supplementary Information E.2). Moreover, as shown for RSV in Zurich in Fig. 3A, real-time *R*_*t*_ estimates remained robust even when the most recent observation was an outlier.

### Estimation of transmission dynamics

Based on measured wastewater concentrations, we estimated the effective reproduction number *R*_*t*_ and epidemic growth rate *r*_*t*_ over time for each pathogen and catchment (Fig. 2A–C and Supplementary Information F). Pronounced seasonal waves were observed for all pathogens in all catchments and seasons, except for SARS-CoV-2 during the 2024/25 season (Supplementary Fig. 25). Maximum *R*_*t*_ values were generally higher for RSV (median estimate 1.41 – 1.47 across catchments in 2023/24) than for SARS-CoV-2 (1.14 – 1.19) and IAV (1.20 – 1.31). This difference likely reflects the longer generation time assumed for RSV in our analysis, rather than faster epidemic growth. Indeed, the epidemic growth rate *r*_*t*_ estimated by EpiSewer was similar across pathogens (Supplementary Figs. 31–33). While the timing of transmission dynamics varied by pathogen, it was largely synchronized across catchments. In 2023/24, we estimated that the peak in new infections (indicated by the median *R*_*t*_ falling below 1) occurred earlier for SARS-CoV-2 (Nov 6 – Dec 2 across catchments), followed by RSV (Dec 13 – Feb 01), and IAV (Dec 15 – Feb 09). Due to pathogen-specific shedding delays, the estimated infection peaks generally preceded peaks in wastewater concentrations.

To model pathogen loads in wastewater based on observed concentration measurements, EpiSewer incorporates the daily wastewater flow at each WWTP, which varies over time due to rainfall and other environmental factors. For instance, during the peak of the 2023/24 SARS-CoV-2 wave, wastewater flow was above its seasonal average at the Zurich WWTP and below its seasonal average at the Lugano WWTP (Fig. 2D). When not using flow data and assuming a constant flow instead, the SARS-CoV-2 wave is underestimated in Zurich and overestimated in Lugano (Supplementary Figs. 11, 12). This highlights the importance of adjusting modeled wastewater concentrations for catchment-specific temporal differences in the dilution of genetic material in sewage.

### Real-time performance

We fitted EpiSewer on each day with new measurement data, obtaining real-time estimates of *R*_*t*_ and short-term forecasts of concentration measurements (see Supplementary Movies 1–3 for real-time animations of model predictions). Figure 3 summarizes the model’s real-time performance across the catchments of Zurich, Lugano, and Chur during the 2023/24 winter season. Real-time *R*_*t*_ estimates showed greater uncertainty near the present but were generally consistent with retrospective estimates computed at the end of each wave (Fig. 3A). For estimates obtained in the same week as the latest observation, the 95% credible intervals of *R*_*t*_ captured the retrospective median *R*_*t*_ on 97–100% of dates for SARS-CoV-2, 88–100% for IAV, and 87–96% for RSV (Fig. 3B). With a one-week lag, retrospective coverage exceeded 88% for all pathogens and reached 100% after a three-week lag. The mean absolute error (MAE) of same-week median *R*_*t*_ estimates was 0.06–0.09 for SARS-CoV-2, 0.05–0.08 for IAV, and 0.11–0.14 for RSV, and fell below 0.04 for all pathogens after a three-week lag (Supplementary Figs. 28–30).

By projecting the recent long-term trend in *R*_*t*_ forward, we generated probabilistic forecasts of concentration measurements 1-14 days ahead. At each estimation date, we computed the forecast bias, which compares predicted quantiles to observations and is optimal when the predicted median matches the observed concentration. We also assessed forecast calibration by comparing the percentage of observations that fall within a given forecast interval with the interval’s nominal coverage. Example forecasts for RSV in Zurich are shown in Fig. 3A. Forecasts were generally well calibrated and showed little bias, indicating accurate prediction of short-term trends (Fig. 3C, D). During periods of increasing infection incidence, forecast bias was consistently above -25%, corresponding to an average underprediction by the 62.5% quantile (with 50% indicating an unbiased forecast). During decreasing infection incidence, forecast bias was consistently below 17%, corresponding to an average overprediction by the 41.5% quantile. Calibration was generally good across all pathogens, with forecast intervals capturing the expected proportion of observations. However, we observed a tendency towards over-coverage at narrow forecast intervals for IAV at smaller catchments. Comparable *R*_*t*_ consistency and forecast performance were achieved in the 2022/23 and 2024/25 winter seasons (Supplementary Figs. 26, 27). In the 2022/23 season, forecasts for IAV and RSV were overdispersed at the catchments of Lugano and Chur, as measurements were only available from November 1, 2022 onward.

### Robustness under sparse sampling

**Fig. 4:**
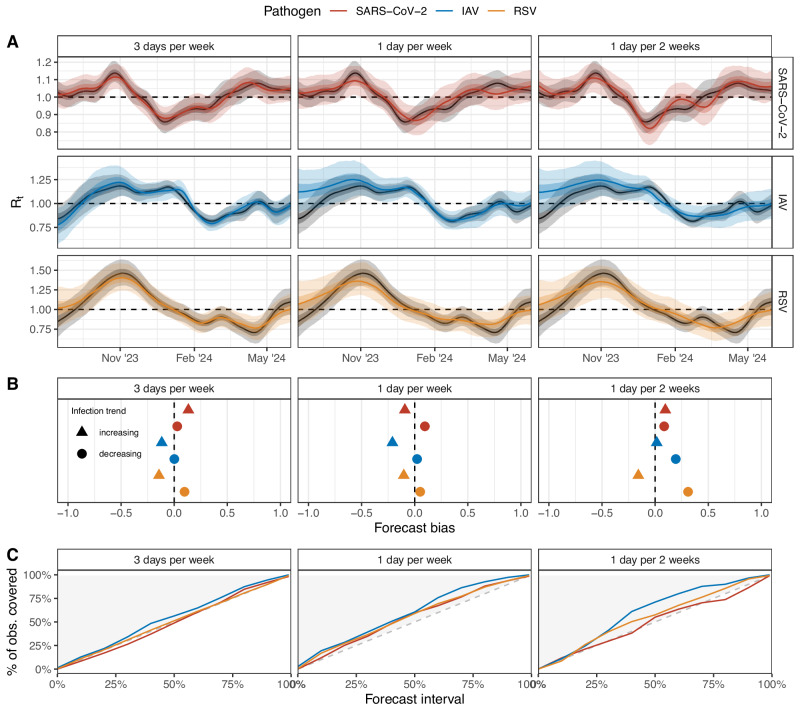
Effects of reduced sampling on effective reproduction number (*R*_*t*_) estimates and concentration forecasts. Concentration measurements of SARS-CoV-2, influenza A virus (IAV), and respiratory syncytial virus (RSV) at the Zurich wastewater catchment, Switzerland, during the winter season 2023/24 were subsampled at different frequencies to simulate reduced sampling. **A** Retrospective *R*_*t*_ estimates on May 31, 2024 based on samples on 3 days per week (Mon/Tue, Wed/Thu, and Fri), 1 day per week (every Fri), and 1 day per 2 weeks (every other Fri). Lines show posterior medians; bands indicate 50% (dark) and 95% (light) credible intervals. The baseline using all available data (5 days/week) is shown in gray. **B** Bias of 1–14-day ahead concentration forecasts from models fitted on subsampled data, stratified by increasing vs. decreasing infection trends. Scores range from − 1 (maximum underprediction) to 1 (maximum overprediction); 0 indicates no bias. **C** Calibration of 1–14-day ahead forecasts from models fitted on subsampled data. The diagonal line indicates perfect calibration, where predictive intervals match their nominal coverage. Source data are provided as a Source Data file.

Due to logistical and cost constraints, many monitoring programs are not able to sustain daily sampling and analysis of wastewater, resulting in gaps in the measurement data. In the model, missing measurements are omitted from the likelihood function, eliminating the need for imputation while naturally accounting for the associated uncertainty. To evaluate the robustness of *R*_*t*_ estimates at lower sampling frequencies, we subsampled the wastewater data (5 sampled days per week on average) to 3 days per week, 1 day per week, and 1 day per 2 weeks. Figure 4A shows the resulting *R*_*t*_ estimates for the catchment of Zurich during the winter season 2023/24. As sampling frequency decreased, *R*_*t*_ estimates became more uncertain but preserved the overall trajectory (Fig. 4A). Across pathogens, the mean absolute error (MAE) of retrospective median *R*_*t*_ estimated from subsampled data was 0.01–0.04 for 3 days per week, 0.01–0.06 for 1 day per week, and 0.01–0.08 for 1 day per 2 weeks (Supplementary Fig. 21). Estimates remained insensitive to the specific weekdays sampled at a frequency of 3 days per week, but became weekday-dependent at lower frequencies (Supplementary Fig. 22). This was particularly observed at the onset of the IAV and RSV seasons, where a high proportion of non-detects lead to a loss of signal for *R*_*t*_ under subsampling (Fig. 4A). The bias of real-time concentration forecasts from subsampled data remained small, however, overdispersion increased when sampling only 1 day per week or less (Fig. 4B, C).

## Discussion

In this study, we developed EpiSewer, a Bayesian model for real-time quantification of pathogen transmission dynamics from wastewater. By explicitly modeling the infection, shedding, and measurement processes that underlie wastewater data, we could parameterize the model using interpretable assumptions about PCR-based measurement error and the pathogen’s generation time and shedding profile (i.e., relative distribution of load over time), which can be informed by independent epidemiological evidence. Unlike other methods that rely on preprocessing steps such as smoothing, imputation, or outlier detection to analyze wastewater data^26^, EpiSewer avoids these steps entirely, reducing dependence on difficult-to-calibrate hyperparameters. This makes our approach well-suited for monitoring a broad range of pathogens through multi-target environmental surveillance^12^, even if historical wastewater and clinical data are limited. As evidence on shedding kinetics remains sparse for many infectious diseases^32,37^, we incorporate uncertainty using a prior distribution over both the mean and coefficient of variation of the shedding profile. We also modeled individual-level variation in shedding intensity, but found this to have minimal influence on inferred transmission dynamics, likely because WWTP-level concentrations alone do not provide sufficient information to distinguish individual variability from sampling and measurement noise.

For the pathogens analyzed in this study, real-time *R*_*t*_ estimates from EpiSewer were generally consistent with retrospective estimates, providing timely information about epidemic growth. This even applied to most same-week estimates, although these were subject to substantial uncertainty, stemming from delays in the transmission signal from wastewater. These inherent delays, resulting from biological lags between infection and pathogen shedding^34^, occur independently of sample logistics or lab processing and are comparable to reporting delays in clinical data, which similarly obscure recent changes in transmission^40^. Our wastewater model captured the uncertainty arising from delayed shedding through wider posterior distributions for *R*_*t*_ near the present. However, point estimates, such as the posterior median, should be interpreted with caution, as they only reliably reflected transmission changes after a lag of 2–3 weeks. As such, a consolidation period may be necessary before using real-time estimates in public health decision-making. Here we note that the reported lags are from the point of data availability; in practice, delays in laboratory processing may add further time before precise estimates are available. Importantly, because our *R*_*t*_ estimates are aligned to infection time rather than observation, the lags described here are not directly comparable to those between wastewater and clinical case data^2^.

A major challenge in evaluating wastewater-based transmission models is the absence of ground truth for key epidemiological parameters such as *R*_*t*_. Previous studies have validated wastewater-based *R*_*t*_ estimates against case-based estimates derived from clinical data, but this approach is prone to biases in case ascertainment and reporting, and is sensitive to the method used to estimate *R*_*t*_ from cases^3,20,24,41^. In this study, we used forecast-based metrics to evaluate the real-time performance of EpiSewer. This approach leverages the close link between real-time transmission dynamics and forecasting of observations in the generative model. Short-term concentration forecasts from EpiSewer showed little bias 1–14 days ahead and were generally well-calibrated, although we observed a slight tendency toward overdispersion in smaller catchments and for pathogens with low detectable concentrations, likely due to reduced signal-to-noise ratios when measurements are low and include many non-detects. These results indicate that the model reliably estimated the current growth trend in new infections, which, beyond *R*_*t*_ estimation, is the basis for downstream tasks such as nowcasting and forecasting clinical cases or hospitalizations^27^. Forecast-based evaluation also enables continuous model assessment during real-time surveillance. For instance, by monitoring the accuracy of flow-adjusted concentration measurement forecasts over time, violated model assumptions about the shedding or measurement of a pathogen could be swiftly detected. Unlike assessments that rely on predicted clinical data^24,26^, this approach requires no auxiliary observations and is particularly suited for non-notifiable diseases that lack clinical reporting. More broadly, forecast-based metrics could be used to benchmark alternative wastewater models, however many existing methods rely on non-parametric smoothing or interpolation of the data and do not naturally yield probabilistic forecasts of measured concentrations^3,20,21,23^. Simulations could also be used to assess real-time transmission dynamic estimates across models, but a fair simulation-based benchmark would require an independent mechanistic model of transmission and wastewater processes, which is beyond the scope of this study.

Due to resource and cost constraints, many wastewater monitoring programs have limited sampling frequency^31^. To evaluate how EpiSewer performs under such conditions, we tested its robustness by subsampling our wastewater data. Due to the model’s generative structure, increased measurement uncertainty from reduced sampling was inherently reflected in the *R*_*t*_ estimates, without requiring parameter adjustments. For the pathogens and sites analyzed, we found that duplicate measurements of 24 h composite samples on three days per week were sufficient to produce accurate *R*_*t*_ estimates with little additional uncertainty. At lower sampling frequencies, e.g., once per week, EpiSewer still captured overall transmission dynamics but produced more uncertain *R*_*t*_ estimates and, at very low concentrations, became sensitive to the specific weekdays sampled. These findings suggest that sampling frequency should ideally be tailored to the monitoring objective. For instance, identifying the onset of a seasonal wave likely requires three or more samples per week, whereas tracking overall *R*_*t*_ during peak transmission may tolerate slightly lower frequencies.

We note several limitations of our model. First, our approach is designed to estimate relative changes in transmission over time rather than absolute numbers of infections in a catchment. The latter are generally difficult to infer from wastewater data alone, due to considerable uncertainty regarding the total amount of pathogen shedding and the loss of detectable genetic material during in-sewer transport and laboratory processing^42,43^. Therefore, the latent parameter *I*_*t*_ in our model should only be used for model inspection and should not be interpreted in terms of absolute incidence. Second, although our model is robust to miscalibration of the average shedding load per infection, it does not account for potential differences in shedding between variants of the same pathogen. For the time period monitored in this study, SARS-CoV-2 infections in Switzerland were consistently dominated by the Omicron variant; however, Wannigama et al. found evidence for increased fecal shedding in the later sublineages BA.2.86 and JN.1^44^. For influenza A virus, both infections with pandemic (H1N1pdm09) and seasonal (H3N2) strains were common during the monitored time period, but we found no clear evidence for marked differences in shedding between these strains^45^. For RSV, there is an overall lack of quantitative evidence on shedding stratified by RSV-A and RSV-B subtypes. Generally, when a variant with substantially different shedding intensity replaces the dominant variant in a population, *R*_*t*_ estimates will be biased for a brief transition period. Importantly, however, this bias is only temporary and vanishes when the new variant has become dominant^46^. This inherent limitation of population-level *R*_*t*_ estimation from wastewater may be overcome through variant-specific *R*_*t*_ estimates, which can be obtained by applying EpiSewer to concentration measurements from variant-specific PCR assays^47^. Alternatively, our model could be extended by integrating wastewater sequencing data to jointly model the total absolute and variant-specific relative loads of a pathogen^48^. Third, to account for the varying dilution of genetic material in wastewater, EpiSewer requires flow data from the monitored sampling site and so is limited to programs using influent wastewater. If flow data are unavailable, a constant flow can be assumed; however, this makes it impossible to account for seasonal fluctuations, e.g., due to rainfall. Alternative approaches using fecal shedding markers to account for variable flow and further factors also exist^49,50^. These could be modeled similarly to flow and are also applicable to solids-based wastewater monitoring. Fourth, our approach is robust to sewer-specific factors influencing detectable gene loads, such as viral degradation and adsorption onto biofilms, as long as these factors do not vary systematically over time. To reliably model time-varying loss of detectable RNA/DNA in the sewer, more research is needed to quantify the effect of fluctuating environmental variables on these phenomena^51,52^. Fifth, we emphasize that clinical evidence on wastewater-related shedding remains limited. This includes uncertainty regarding the predominant sources of shedding into wastewater, e.g., urine, feces, nasal secretions, saliva, etc., for most pathogens. For IAV and RSV, we used available evidence from nasal samples to parameterize the shedding profile, noting that the relative contributions of fecal and oral shedding into wastewater remain largely unclear. Moreover, when modeling individual-level variation in shedding, we accounted only for differences in the absolute amount of genetic material shed, not for variation in the timing of shedding across individuals, as there is currently a lack of data to parameterize such models^34,53^. However, for larger catchment populations, we expect the dominant source of uncertainty in wastewater loads to stem from uncertainty in the average shedding profile and from variation in the total amount shed, which we explicitly model in our approach. Sixth, we used a realistic error model tailored to concentration measurements from digital PCR, which is widely used in wastewater surveillance due to its reliable quantification of absolute target concentrations^54^. Other studies, however, often rely on quantitative PCR, which can be modeled in EpiSewer using simpler likelihood functions such as the log-normal distribution. Quantitative PCR has similar error characteristics as digital PCR, including the occurrence of non-detects^30^, which EpiSewer also supports through a hurdle model likelihood. Finally, we modeled the infection process using a stochastic renewal model that does not account for population structure or differences in population size. However, the population contributing to transmission may differ from the wastewater catchment population, and the latter may vary due to human mobility, with potential impacts on the total load shed in the catchment over time^32^. While sensitivity analysis suggests that our approach is robust to regular weekly fluctuations in the population size, such as those caused by commuting (Supplementary Information E.5), more irregular changes in the catchment population, e.g., due to non-pharmaceutical interventions or tourism^55,56^, could bias estimates of transmission dynamics if unaccounted for. Incorporating these factors, e.g., using shedding markers, mobility data and information on contact patterns, can be particularly important when modeling low-prevalence pathogens, but will require a better understanding of wastewater-relevant shedding activities and their relationship with human mobility.

We implemented the EpiSewer model as a user-friendly and configurable open-source R package to support its use across different surveillance settings^57^. As part of our ongoing multi-pathogen wastewater surveillance in Switzerland, we will continue to publish up-to-date reproduction number estimates on our public dashboard (https://wise.ethz.ch) for Swiss health authorities and the general public. As wastewater monitoring expands to new pathogens of interest, semi-mechanistic modeling of wastewater data, as demonstrated in this work, allows robust estimation of transmission dynamics in real time and provides the necessary interpretability to check for violated epidemiological assumptions and other forms of model misspecification. This will enable more detailed analysis of wastewater data to complement traditional public health surveillance and provide insights into the transmission of clinically under-monitored and emerging infectious diseases.

## Methods

### Ethics statement

No ethics approval was required for this population-level wastewater study because no human participants or identifiable personal data were involved. Written informed consent was not applicable.

### Wastewater data

In November 2022, Eawag, Swiss Federal Institute of Aquatic Science and Technology, launched the combined monitoring of SARS-CoV-2, influenza A and B virus (IAV and IBV) and respiratory syncytial virus (RSV) in Swiss wastewater^5,39^. Autosampler-based, 24 h volume-proportional composite samples of raw influent wastewater were collected on average 5 days per week from 6 municipal WWTPs across Switzerland and analyzed using a droplet-based, four-plex digital PCR (dPCR) assay for SARS-CoV-2 (*N2* gene), influenza A virus (*M* gene), influenza B virus (*M* gene), and RSV (*N* gene), as previously described in Nadeau et al.^5^. Target concentrations in gene copies per mL were measured using two technical replicates per sample and summarized by the arithmetic mean. In July 2023, an improved six-plex dPCR assay with two additional targets (*N1* gene of SARS-CoV-2, *M* gene of Murine Hepatitis Virus for extraction efficiency control) was introduced, and monitoring was expanded to a total of fourteen WWTPs^39^. The catchment areas of the treatment plants vary in size from 16500 to 471000 inhabitants and together cover 27% of the Swiss population. Since January 2025, the monitoring was downsized to ten WWTPs. Overall, *n* = 6352 samples were included across the study period (Nov 2022 – May 2025). Further details on the treatment plants, analyzed samples, extraction, and preprocessing are provided in Supplementary Information A. In our analysis, we used the *N2* gene as a target for SARS-CoV-2, which was measured by both the four-plex and the six-plex assay. Furthermore, since IBV did not consistently show epidemic waves during the monitored seasons, we only included results for IAV. To account for the varying dilution of wastewater due to rainfall and other factors, we used the daily flow measured at each treatment plant in our analysis.

### Model definition

In the following, we define the EpiSewer model, a Bayesian generative model of wastewater data that can be used to estimate the effective reproduction number *R*_*t*_ and other measures of transmission. Our model applies to a time series of concentration measurements for a specific sampling site and target gene. We use a unified time index *t* ∈ {1, 2, …, *T*} for all time-dependent variables, where *t* = 1 denotes the first day of the modeled infection trajectory and *t* = *T* the day of the last wastewater measurement. Due to delays in shedding from infected individuals, we also model infections before the first wastewater measurement, i.e., wastewater measurements start only on day *t* = 1 + *S*, with shedding of genetic material up to *S* days after infection.

To model daily measured pathogen concentrations *c*_*t*_, we use a dPCR-specific likelihood from Lison et al.^58^ that accounts for concentration-dependent measurement noise and false negatives. Specifically, we let *λ*_*t*_ be the expected target concentration in the wastewater sample collected on day *t*. Using *p*_zero_(*λ*_*t*_), the probability of false non-detection from dPCR, we define a hurdle model likelihood for dPCR measurements (Supplementary Information B), i. e.,

1 $${L}_{{C}_{t}}({c}_{t}| {\lambda }_{t})=\left\{\begin{array}{ll}{p}_{{{{\rm{zero}}}}}({\lambda }_{t}),\hfill&\,{\mbox{if}}\,{c}_{t}=0\\ (1-{p}_{{{{\rm{zero}}}}}({\lambda }_{t}))\,{p}_{{{{\rm{Gamma}}}}}({c}_{t};\,{\mu }_{t},\nu ({\lambda }_{t})),\quad &\,\quad{\mbox{otherwise}}\,\end{array}\right.,$$

where *p*_Gamma_ is the probability density of non-zero concentrations, modeled as gamma distributed with mean ${\mu }_{t}=\frac{{\lambda }_{t}}{1-{p}_{{{{\rm{zero}}}}}({\lambda }_{t})}$ and coefficient of variation *ν*(*λ*_*t*_). This likelihood accounts for the partitioning statistics of dPCR^29^ and allows direct fitting to concentration measurements, including non-detects. We note that *p*_zero_(*λ*_*t*_) and *ν*(*λ*_*t*_) depend on lab-specific parameters such as the pre-PCR coefficient of variation and the volume and number of partitions in the dPCR assay, which we jointly estimate from the measurement data (Supplementary Information C.1).

Assuming a sewer residence time of less than one day and uniform mixing of genetic material in the wastewater^3^, we model the expected concentration *λ*_*t*_ in a 24 h composite sample as

2 $${\lambda }_{t}=\frac{{\omega }_{t}}{{{\mbox{flow}}}_{t}}+{\epsilon }_{t},$$

where *ω*_*t*_ is the daily expected detectable load shed in the catchment, flow_*t*_ is the measured flow (i.e., daily wastewater volume) at the sampling site, and *ϵ*_*t*_ > 0 represents positive outlier concentrations that violate the uniform mixing assumption. We use *ϵ*_*t*_ to model occasional spikes in concentrations as regularly found in wastewater data^59^. As a prior for *ϵ*_*t*_, we use a Type II extreme value distribution with parameters chosen such that 99% of the probability mass is below a concentration that would be observable from one infection in the catchment, which matches historically observed outlier frequencies in our data (Supplementary Information C.4). This heavily right-tailed prior can capture extreme spikes in concentrations while having a negligible influence on modeled concentrations on most days.

The expected load shed in the catchment on day *t*, denoted *ω*_*t*_, depends on the number of individuals infected on previous days and their intensity of shedding *s* days after infection. We model *ω*_*t*_ via a convolution

3 $${\omega }_{t}={\sum }_{s=0}^{S}{\mu }^{{{{\rm{load}}}}}\,{\tau }_{s}^{\,{\mbox{shed}}\,}\,{\zeta }_{t-s},$$

where *μ*^load^ is the average detectable total shedding load per person, ${{{{\boldsymbol{\tau }}}}}^{{{{\rm{shed}}}}}=({\tau }_{0}^{{{{\rm{shed}}}}},{\tau }_{1}^{{{{\rm{shed}}}}},\ldots,{\tau }_{S}^{{\mbox{shed}}\,})$ is a discrete distribution representing the temporal shedding profile (i.e., the relative distribution of load shed by an individual over time), and ${\zeta }_{t} \sim \,{\mbox{Gamma}}\,(\alpha=\frac{{I}_{t}}{{\nu }_{\zeta }^{2}},\beta=\frac{1}{{\nu }_{\zeta }^{2}})$ is the number of newly infected individuals *I*_*t*_, weighted by their overall shedding intensity. Here, the coefficient of variation *ν*_*ζ*_ describes how the strength of shedding varies across individuals, with an intensity  < 1 indicating below-average shedding and an intensity  > 1 indicating above-average shedding. We obtain the temporal shedding profile by placing priors on the mean *μ*^shed^ and coefficient of variation *ν*^shed^ of a positive continuous distribution (e.g., gamma or log-normal), and transforming it into a discrete distribution ***τ***^shed^ within the model (Supplementary Information C.2). This approach has been previously used to model reporting delay distributions^60^ and here allows us to account for uncertain shedding profiles.

To model the number of individuals newly infected on day *t*, we use a stochastic renewal model similar to earlier work^40,61^. This defines the expected number of new infections *ι*_*t*_ through the renewal equation

4 $${\iota }_{t}={{{\rm{E}}}}[{I}_{t}]={R}_{t}{\sum }_{s=1}^{G}{\tau }_{s}^{\,{\mbox{gen}}\,}\,{I}_{t-s}\,| \,t > G,$$

where *R*_*t*_ is the instantaneous effective reproduction number on day *t*^13^ and ${{{{\boldsymbol{\tau }}}}}^{{{{\rm{gen}}}}}=({\tau }_{1}^{{{{\rm{gen}}}}},{\tau }_{2}^{{{{\rm{gen}}}}},\ldots,{\tau }_{G}^{{\mbox{gen}}\,})$ is a discrete generation time distribution with maximum generation time *G*. The realized number of new infections *I*_*t*_ is modeled as Poisson distributed, i. e.

5 $${I}_{t}| {\iota }_{t} \sim {{{\rm{Poisson}}}}({\iota }_{t}).$$

At the start of the modeled time period, when the renewal model cannot be applied, we model a seeding phase^62^ in which the expected number of new infections follows an exponential growth process with initial value *ι*_1_ and a time-varying growth rate *r*_*t*_, i. e.

6 $$\log ({\iota }_{t})=\log ({\iota }_{t-1})+{r}_{t},\,\,{r}_{t}| {r}_{t+1} \sim {{{\mathcal{N}}}}({r}_{t+1},{{\sigma }_{r}}^{2})\,\,| \,1 < t\le G,$$

where *r*_*t*_ follows a backward-in-time random walk with initial value *r*_*G*+1_ and standard deviation *σ*_*r*_. We set *r*_*G*+1_ to be consistent with the reproduction number *R*_*t*_ at the end of the seeding phase by solving the renewal equation ${R}_{G+1}(\mathop{\sum }_{s=1}^{G}{\tau }_{s}^{\,{\mbox{gen}}\,}{e}^{-s{r}_{G+1}})=1$^63^. If there are many non-detects at the beginning of the measured time series, we extend the seeding phase until the first date with three consecutive detects. From the estimated number of infections, we also compute *r*_*t*_ for *t* > *G* using an infectiousness-based estimator by Parag et al.^64^ (Supplementary Information C.5).

### Priors and epidemiological assumptions

We informed model parameters related to infection and shedding based on findings from the epidemiological literature. For the generation time distribution, we obtained parametric distributions for the serial interval for SARS-CoV-2^65^, human influenza^66^, and RSV^67,68^ and discretized them with a daily resolution. For the temporal shedding profile, i.e., the relative distribution of the shedding load over time, we used uncertainty information from estimates in the literature to define appropriate priors for the mean and coefficient of variation. For SARS-CoV-2, we used a gamma distribution with a mean of 8.0–17.7 days, based on an observational study that combined wastewater measurements with case surveillance data collected between August and December 2020^35^. Although this study characterized shedding for the SARS-CoV-2 wild type, its estimates are consistent with later clinical evidence on shedding kinetics for several Omicron subvariants^44^. For IAV and RSV, we used the relative distribution of viral concentration measurements from nasal wash or swab samples as a proxy for shedding into wastewater over time, assuming constant material loads over the course of infection. Note that for other pathogens, specifically gastrointestinal viruses, time-varying material loads might need to be accounted for. For IAV, we used a gamma distribution with a mean of 2.1–2.9 days based on a meta-analysis of volunteer challenge studies^5,69^. For RSV, we used a gamma distribution with a mean of 4.4–9.2 days based on a household study by Otomaru et al.^70^. Details on the discretization of the shedding profile in our model and the priors used for each pathogen are provided in Supplementary Information C.2.

We assumed a pathogen- and catchment-specific total detectable load per infection *μ*^load^, i.e., the absolute number of gene copies shed by an infected individual and detectable from wastewater samples^3^, which also depends on the degradation of genetic material during in-sewer transport and storage^52^ and extraction efficiency in the lab. In our main analysis, we calibrated *μ*^load^ to catchment-specific infection numbers estimated using publicly available data from Sentinella reporting and participatory surveillance in Switzerland (Supplementary Information C.3). Generally, case data used for calibration cannot be assumed unbiased due to imperfect ascertainment and reporting, and any bias in the calibration of *μ*^load^ will affect the estimated number of new infections *I*_*t*_ in our model. However, as *μ*^load^ scales *I*_*t*_ only by a constant factor, transmission dynamic estimates remain invariant to such miscalibration except when incidence is low enough for stochastic infection and shedding dynamics to dominate (Supplementary Fig. 18). Accordingly, we obtained practically identical *R*_*t*_ estimates for most catchments using a simpler calibration without case data, in which the initial load detected at the start of each season was equated to an incidence of 0.01% of the catchment population (Supplementary Figs. 15–17). Similarly, estimates remain robust when calibrating *μ*^load^ using case data with time-varying reporting intensity (Supplementary Fig. 19), which represents an important advantage over joint inference from case and wastewater data.

To account for heterogeneity in shedding^34,37,53,71^, we modeled individual-level variability in shedding loads. Due to the lack of quantitative estimates in the literature, we assumed a coefficient of variation of 1, implying that the top 25% of individuals contribute 60% of the total load, and the bottom 25% less than 4%, consistent with observed SARS-CoV-2 shedding variability^34^. Sensitivity analysis showed that transmission estimates are largely insensitive to this assumption (Supplementary Fig. 20).

To regularize the estimated *R*_*t*_ trajectory, we decomposed the reproduction number into a long-term trend (*α*_*t*_) and a short-term deviation (*δ*_*t*_), each modeled with an approximate Gaussian process^60,72^ using long- and short-term kernels, respectively, i. e.,

7 $$\alpha \sim {{{\mathcal{GP}}}}\,\,\left(1,\,{k}_{\nu }(\tau ;\,{\ell }_{\alpha },{\sigma }_{\alpha }^{2})\right),\,\delta \sim {{{\mathcal{GP}}}}\,\,\left(0,\,{k}_{\nu }(\tau ;\,{\ell }_{\delta },{\sigma }_{\delta }^{2})\right),$$

with Matérn kernel *k*_*ν*_ and smoothness parameter $\nu=\frac{3}{2}$. For the long-term trend, we used truncated normal priors on the length scale *ℓ*_*α*_ (mean 12 weeks, SD 1 week) and kernel magnitude *σ*_*α*_ (mean 0.25, SD 0.05). For the short-term deviation, we placed truncated normal priors on *ℓ*_*δ*_ (mean 3 weeks, SD 0.5 weeks) and *σ*_*δ*_ (mean 0.125, SD 0.025). To ensure *R*_*t*_ > 0, we computed the reproduction number as *g*(*R*_*t*_) = *α*_*t*_ + *δ*_*t*_, where $g(x)=\frac{1}{k}\log \left({e}^{kx}-1\right)$ is an inverse softplus link with sharpness parameter *k* = 4, yielding an approximately linear mapping for realistic *R*_*t*_ values. The smoothing of *R*_*t*_ is described in more detail in Supplementary Information C.5. For the laboratory parameters of our dPCR-specific measurement model, we used broad priors reflecting typical dPCR instruments and protocols (Supplementary Information C.1).

### Estimation

Our model defines a likelihood for observed concentration measurements **c** given *R*_*t*_, measured flow, and further parameters related to the infection (**θ**^inf^), shedding (**θ**^shed^), and measurement (**θ**^meas^) process (see Supplementary Table 3 for a detailed description of all estimated parameters). We use Markov Chain Monte Carlo to draw samples from the posterior distribution of *R*_*t*_, i.e.,

8 $$P\left({{{{\bf{R}}}}}_{\{t| 1+G\le t\le T\}},\,{{{{\mathbf{\theta }}}}}^{{{{\rm{inf}}}}},\,{{{{\mathbf{\theta }}}}}^{{{{\rm{shed}}}}},{{{{\mathbf{\theta }}}}}^{{{{\rm{meas}}}}}| {{{{\bf{c}}}}}_{\{t| 1+S\le t\le T\}}\right)\propto$$

9 $$P\left({{{{\bf{c}}}}}_{\{t| 1+S\le t\le T\}}| {{{{\bf{R}}}}}_{\{t| 1+G\le t\le T\}},\,{{{{\mathbf{\theta }}}}}^{{{{\rm{inf}}}}},\,{{{{\mathbf{\theta }}}}}^{{{{\rm{shed}}}}},{{{{\mathbf{\theta }}}}}^{{{{\rm{meas}}}}}\right)\times P\left({{{{\bf{R}}}}}_{\{t| 1+G\le t\le T\}}\right)\times$$

10 $$P\left({{{{\mathbf{\theta }}}}}^{{{{\rm{inf}}}}}\right)\times P\left({{{{\mathbf{\theta }}}}}^{{{{\rm{shed}}}}}\right)\times P\left({{{{\mathbf{\theta }}}}}^{{{{\rm{meas}}}}}\right),$$

with priors $P\left({{{{\bf{R}}}}}_{\{t| 1+G\le t\le T\}}\right)$, $P\left({{{{\mathbf{\theta }}}}}^{{{{\rm{inf}}}}}\right)$, $P\left({{{{\mathbf{\theta }}}}}^{{{{\rm{shed}}}}}\right)$, and $P\left({{{{\mathbf{\theta }}}}}^{{{{\rm{meas}}}}}\right)$ for *R*_*t*_ and the infection, shedding, and measurement-related parameters, respectively. For this, we implemented our model in the probabilistic programming language stan^73^. We fitted the model by running 4 chains, each with 1000 warm-up and 2500 sampling iterations, of the No-U-Turn Sampler (NUTS) in cmdstan v2.34.1 through the cmdstanr v0.9.0 interface^74^. We assessed convergence and effective sample sizes using standard Bayesian diagnostics^75,76^. Further details on the model implementation, sampling, average runtimes, and diagnostic checks can be found in Supplementary Information D. The model is integrated into the open-source R package EpiSewer^57^, which facilitates model specification, fitting, and visualization of results. Analyses were conducted in R v4.5.2, using R packages dplyr v1.1.4, tidyr v1.3.1, and data.table v1.17.8 for data processing, ggplot2 v4.0.0 for plotting, and targets v1.11.4 for pipeline orchestration.

### Forecasting

To produce short-term forecasts of concentration measurements, we projected transmission dynamics forward by evaluating the Gaussian processes for the long-term trend and short-term deviations in *R*_*t*_ up to 14 days into the future. Due to the different length scales of the long-term and short-term components, forecasts naturally tend towards the recent long-term trend. For each posterior sample from the fitted model, we then propagated the renewal equation forward using the forecasted *R*_*t*_ trajectory and other sampled parameters, applying the shedding and measurement components to simulate dPCR measurements on the forecasted dates. By combining simulated measurements from all posterior samples, we obtained a posterior predictive distribution for future concentration measurements. We note that to predict concentrations, the unknown future wastewater flow was imputed with its historically observed median value. For the retrospective evaluation of forecasts, we used flow-adjusted concentration measurements to avoid bias from the imputation of unknown future flow at the time of prediction.

### Real-time performance and subsampling study

We assessed the performance of real-time estimation during seasonal infection waves of SARS-CoV-2, IAV, and RSV. For each pathogen, we defined the start of the seasonal wave as the first week in the second half of the year with a 10% weekly growth rate and no consecutive non-detects before the peak. We defined the end of a seasonal wave as the first week three months after the peak, with a decrease in weekly incidence of less than 10%. On each day of the wave with new measurements, we obtained real-time *R*_*t*_ estimates for the respective pathogen by fitting EpiSewer to all measurements up to that date, using data from 1 month before the start of the wave. For each date, we also produced real-time forecasts of the measured concentrations for the next 14 days. We note that in the winter season 2022/23, measurements for Lugano, Chur, Sensetal, and Altenrhein were only available from November 1, 2022 onward. Posterior *R*_*t*_ and forecast distributions were summarized using the median and the 1%, 3%, … , 97%, and 99% credible intervals. Real-time estimates were compared to a retrospective estimate based on all measurements up to August 1 of the following year (June 1 for 2025). For this, we calculated the percentage of days on which the retrospective *R*_*t*_ estimate was covered by the 95% credible interval of the real-time estimate, stratified by lag. To evaluate the performance of short-term concentration forecasts, we computed scores for forecast calibration^77^, i.e., the percentage of observations within nominal forecast intervals, and forecast bias^78^, i.e.,

11 $${B}_{t}=(1-2\cdot \max \{i| {q}_{t,i}\in {Q}_{t}\wedge {q}_{t,i}\le {l}_{t}\}){{{{\bf{1}}}}}_{{l}_{t}\le {q}_{t,0.5}} \\+\,(1-2\cdot \min \{i| {q}_{t,i}\in {Q}_{t}\wedge {q}_{t,i}\ge {l}_{t}\}){{{{\bf{1}}}}}_{{l}_{t}\ge {q}_{t,0.5}},$$

where *l*_*t*_ = *c*_*t*_ × flow_*t*_ is the flow-adjusted concentration measurement and *Q*_*t*_ is the set of lower and upper quantiles of the credible intervals of the forecast distribution for day *t*. Calibration curves and bias were averaged across forecasts and estimation dates. To mimic sparse sampling, we subsampled SARS-CoV-2, IAV, and RSV measurement time series from the Zurich catchment during the winter season 2023/24 to lower frequencies, i.e., 3 days per week (Mon/Tue, Wed/Thu, and Fri), 1 day per week (every Fri), and 1 day per 2 weeks (every other Fri). The original time series had a frequency of 5 days per week, with measurements missing only on Mon and Wed in one week and on Tue and Thu the following week. When subsampling, we replaced days with missing measurements in the original series by the next day when possible. To evaluate the real-time performance of EpiSewer on subsampled data, we measured the calibration and bias of short-term concentration forecasts as described above. To examine the impact of the specific weekdays sampled, we computed retrospective *R*_*t*_ estimates for all possible weekday combinations of each subsampling frequency.

### Reporting summary

Further information on research design is available in the Nature Portfolio Reporting Summary linked to this article.

## Supplementary information

Supplementary Information

Description of additional supplementary files

Supplementary Movie 1

Supplementary Movie 2

Supplementary Movie 3

Reporting Summary

Transparent Peer Review File

## Source data

Source Data

## Data Availability

This work is based on a secondary analysis of open wastewater data provided by Eawag, Swiss Federal Institute of Aquatic Science and Technology, under a CC BY 4.0 license. The data are publicly available at https://github.com/adrian-lison/EpiSewer-study^79^ and from the public wastewater dashboard at https://wise.ethz.ch. Source data are provided in this paper.

## Code availability

All code and analysis scripts are publicly available at https://github.com/adrian-lison/EpiSewer-study^79^. The EpiSewer model is available as an open-source R package at https://github.com/adrian-lison/EpiSewer.
