# Supplementary material for: Real-time estimation of pathogen transmission dynamics from wastewater
Source: Nat Commun. 2026 Jul 11;17:8563. doi: 10.1038/s41467-026-75380-3 (PMC13483917; doi:10.1038/s41467-026-75380-3)
Supplement: Supplementary file 1 [file 41467_2026_75380_MOESM1_ESM.pdf]

Supplementary information for: Real-time estimation of pathogen transmission dynamics from wastewater

Adrian Lison*, Rachel E. McLeod, Jana S. Huisman, James D. Munday, Christoph Ort, Timothy R. Julian, and Tanja Stadler

*Corresponding author: adrian.lison@bsse.ethz.ch

Contents

A Wastewater data **2**

B Modeling measurements from digital PCR **6**

B.1 Probability of non-detection 6

B.2 Distribution of non-zero measurements 7

C Priors and epidemiological assumptions **8**

C.1 Digital PCR parameters 8

C.2 Generation time distribution and shedding profile 9

C.3 Total shedding load per infection 10

C.4 Outliers 12

C.5 Transmission dynamics 14

D Estimation **16**

D.1 Parameters 16

D.2 Implementation 16

D.3 Sampling and diagnostics 18

E Sensitivity analyses **23**

E.1 Flow data 23

E.2 Outlier detection 24

E.3 Shedding load 26

E.4 Subsampling 32

E.5 Catchment population size 34

F Additional results **36**

Supplementary References **56**

A Wastewater data

We used data from three seasons of wastewater surveillance conducted by the Swiss Federal Institute of Aquatic Science and Technology (EAWAG). During the respiratory virus season 2022/23, samples were collected since November 2022 from 6 wastewater treatment plants and analyzed with a frequency of 5 days per week, i. e. all weekdays except for Wednesdays and Fridays (Supplementary Fig. 1). During the season 2023/24, samples were collected from 14 wastewater treatment plants and analyzed with a frequency of 5 days per week, using a biweekly sampling scheme that varied between batch groups (Supplementary Fig. 2). During the season 2024/2025, samples were collected from 10 wastewater treatment plants and analyzed with a frequency of 4 days per week, again using a biweekly sampling scheme that varied between batch groups (Supplementary Fig. 3). The population of the associated catchment of each wastewater treatment plant is given in Supplementary Figs. 1 to 3, respectively.

The specific extraction and quantification protocol used has been described earlier in Huisman et al. [1] and Nadeau et al. [2]. Briefly, vacuum filtration was used to extract viral RNA from a 40 mL aliquot of each sample. The resulting elution of 80 µL was diluted by a factor of 1:3. A 25 µL reaction mix with 5 µL of the template and 20 µL of reagents was created. Target concentrations were then quantified by running a droplet-based, multiplex digital PCR assay using the Naica Crystal Digital PCR System (Stilla® Technologies). Sapphire Chips with a maximum number of 30000 droplets and an average droplet volume of 0.519 nL were used. Each sample was run in duplicate, and we used the arithmetic mean of the replicate measurements for analysis.

During the respiratory virus season 2022/23, a fourplex digital PCR assay with the targets SARS-CoV-2 (N2 gene), Influenza A (M gene), Influenza B (M gene), and RSV (N gene) was used. For the seasons 2023/24 and 2024/2025, a similar, six-plex assay with two additional targets (N1 gene of SARS-CoV-2, M gene of Murine Hepatitis Virus for extraction efficiency control) was used. Here, we focused our analysis on the targets SARS-CoV-2 (N2 gene), Influenza A (M gene), and RSV (N gene), which were consistently found across seasons.

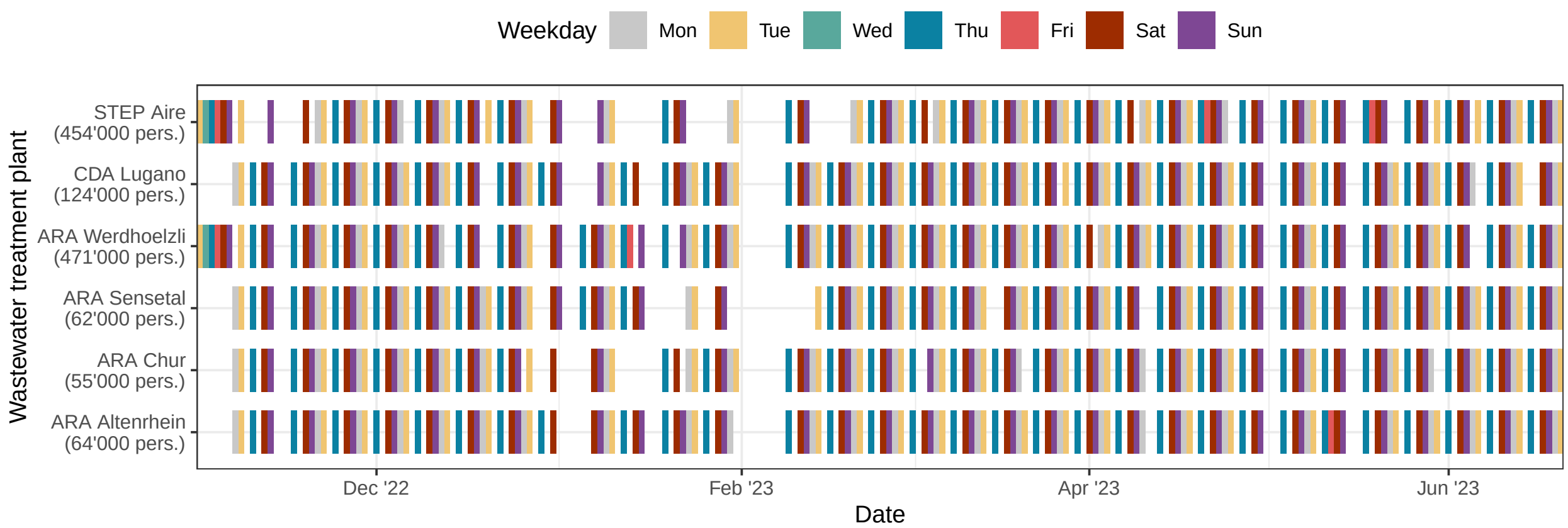

Supplementary Fig. 1. Overview of analyzed samples from 6 wastewater treatment plants during the 2022/23 respiratory virus season in Switzerland. As a rule, 5 samples per week (all weekdays except for Wednesdays and Fridays) from each treatment plant were analyzed. For each sample, the concentration of SARS-CoV-2 (N2 gene), Influenza A and B virus (M gene), and Respiratory syncytial virus (N gene) was quantified in duplicate using a fourplex digital PCR assay. Only samples which passed quality control are shown. Source data are provided as a Source Data file.

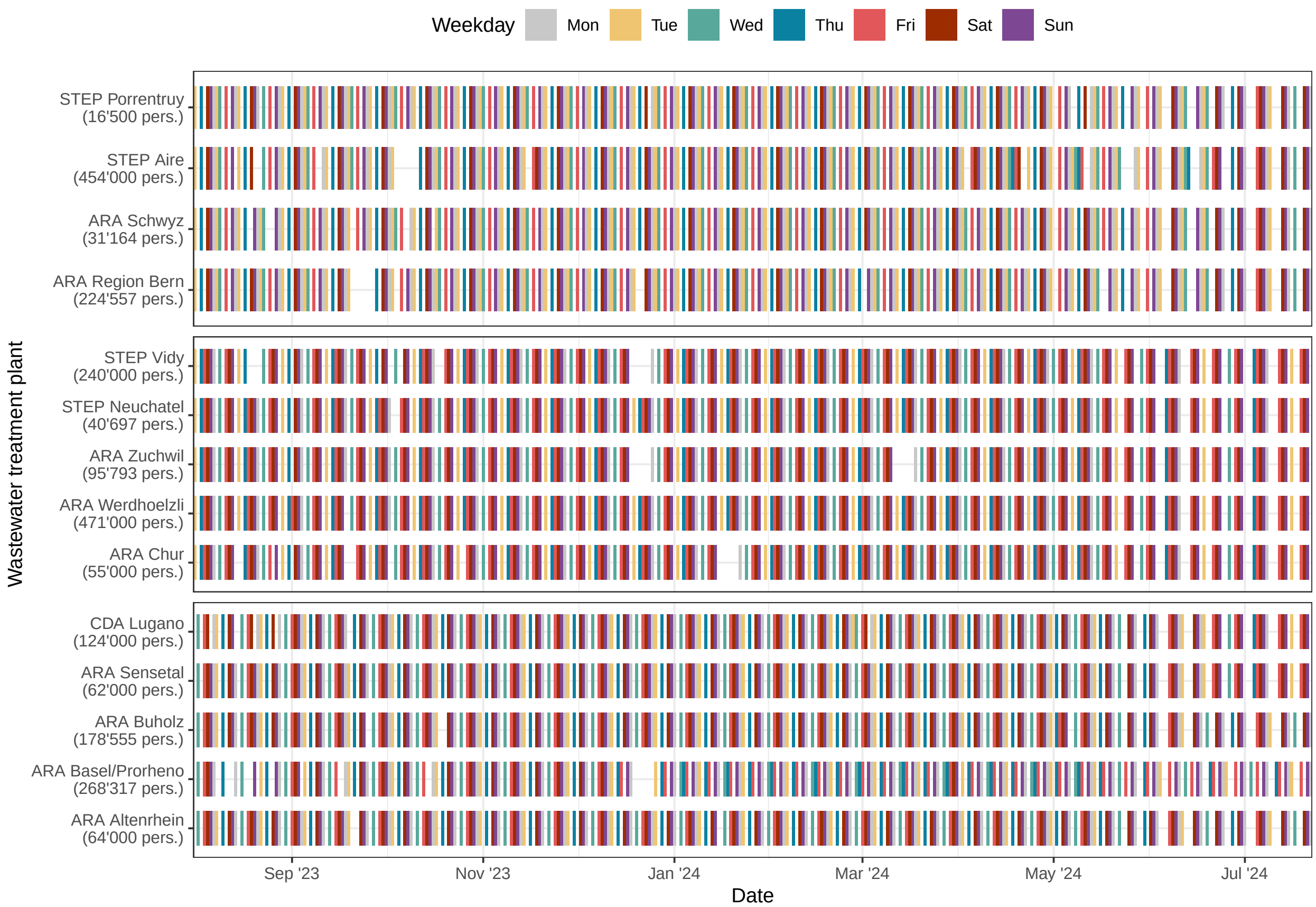

Supplementary Fig. 2. Overview of analyzed samples from 14 wastewater treatment plants during the 2023/24 respiratory virus season in Switzerland. As a rule, 5 samples per week from each treatment plant were analyzed. The specific weekdays analyzed varied between batch groups and alternated on a biweekly basis. For each sample, the concentration of SARS-CoV-2 (N1 and N2 genes), Influenza A and B virus (M gene), Respiratory syncytial virus (N gene), and Murine Hepatitis Virus (M gene, internal control) was quantified in duplicate using a six-plex digital PCR assay. Only samples which passed quality control are shown. Source data are provided as a Source Data file.

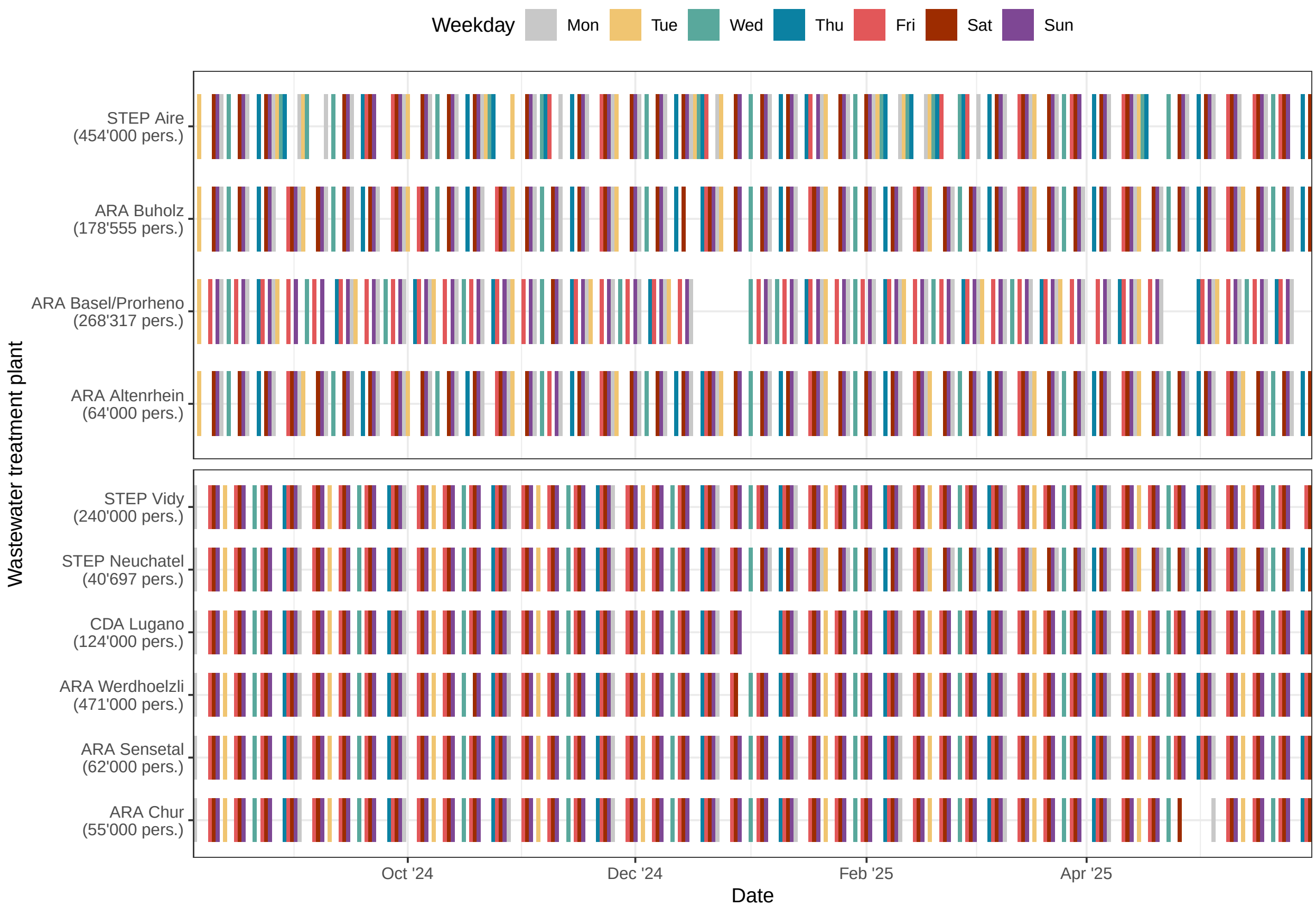

Supplementary Fig. 3. Overview of analyzed samples from 10 wastewater treatment plants during the 2024/25 respiratory virus season in Switzerland. As a rule, 4 samples per week from each treatment plant were analyzed. The specific weekdays analyzed varied between batch groups and alternated on a biweekly basis. For each sample, the concentration of SARS-CoV-2 (N1 and N2 genes), Influenza A and B virus (M gene), Respiratory syncytial virus (N gene), and Murine Hepatitis Virus (M gene, internal control) was quantified in duplicate using a six-plex digital PCR assay. Only samples which passed quality control are shown. Source data are provided as a Source Data file.

B Modeling measurements from digital PCR

We assume that given the expected concentration in wastewater (λ_t), the concentration in a PCR reaction mix is unbiased but subject to a random multiplicative error η_t with mean 1 and coefficient of variation ν_{pre}. This "coefficient of pre-PCR variation" describes all unexplained variation in target concentrations before the PCR quantification, including noise from wastewater sampling and from extraction and preprocessing. In a digital PCR reaction, each partition is assumed to have an expected number of target molecules $\Theta = \lambda_t \eta_t \kappa$, where the conversion factor κ accounts for the partition volume as well as any relative differences in concentration between the original sample and the reaction mix, e. g. due to dilution and addition of reagents. For each replicate run i of the dPCR assay, we also model the total number of valid partitions m_i to account for partition loss.

B.1 Probability of non-detection

From the Poisson count statistics of positive partitions in digital PCR, it can be shown [3] that under the above assumptions, the probability of non-detection, i. e. a zero measurement, is

$$p_{\text{zero}}(\lambda_t) = \mathbb{E}[\exp(-\Theta \sum_{i=1}^{n} m_i)], \tag{1}$$

where n is the number of technical replicates of the sample. The expected value $\mathbb{E}[\exp(-\Theta \sum_{i=1}^{n} m_i)]$ can be obtained from the moment-generating function (MGF) of Λ_t^{pre}. Here we assume that the pre-PCR variation is log-normal distributed, and use an approximation of the MGF of the log-normal distribution by Asmussen et al. [4], i. e.

$$\mathbb{E}[\exp(-\Theta \sum_{i=1}^{n} m_i)] \approx \frac{1}{\sqrt{1+w}} \exp\left(-\frac{(w^2+2\,w)}{2\log(1+\nu_{\text{pre}}^2)}\right), \tag{2}$$

where w is an evaluation of the Lambert W function [5] with

$$w = W\left(-\lambda_t \kappa \sum_{i=1}^{n} m_i \frac{\log(1+\nu_{\text{pre}}^2)}{\sqrt{1+\nu_{\text{pre}}^2}}\right). \tag{3}$$

As described in the main text, the probability of non-detection $p_{\text{zero}}(\lambda_t)$ is used in the hurdle model as the likelihood of zero measurements, and to adjust the distribution of non-zero measurements for

the conditioning on detection.

B.2 Distribution of non-zero measurements

We characterize the distribution of non-zero measurements by the mean and relative variance conditional on detection. As we assume an unbiased assay, the conditional mean of non-zero measurements is

$$\mathbb{E}\left[\hat{\Lambda}_t \middle| \hat{\Lambda}_t > 0\right] = \frac{\mathbb{E}\left[\hat{\Lambda}_t\right]}{P(\hat{\Lambda}_t > 0)} = \frac{\mathbb{E}\left[\hat{\Lambda}_t\right]}{1 - p_{\text{zero}}(\lambda_t)} = \frac{\lambda_t}{1 - p_{\text{zero}}(\lambda_t)}. \tag{4}$$

Moreover, the unconditional asymptotic variance of a concentration estimate from n technical replicates is

$$\text{Var}[\hat{\Lambda}_t] = \lambda_t^2 \, \nu_{\text{pre}}^2 + \frac{1}{\kappa^2 \sum_{i=1}^n m_i} \left(\mathbb{E}[\exp(\Theta)] - 1\right), \tag{5}$$

where $\mathbb{E}[\exp(\Theta)]$ can again be obtained from the moment-generating function (MGF) of Λ_t^{pre} [3]. For this, we again use the approximation of the log-normal MGF from Eq. (2), but now let

$$w = W\left(\lambda_t \, \kappa \, \frac{\log(1 + \nu_{\text{pre}}^2)}{\sqrt{1 + \nu_{\text{pre}}^2}}\right). \tag{6}$$

Using the unconditional variance from Eq. (5), the conditional relative variance of non-zero measurements is [3]

$$\nu^2(\lambda_t) = \frac{\text{Var}[\hat{\Lambda}_t]}{\lambda_t^2} (1 - p_{\text{zero}}(\lambda_t)) - p_{\text{zero}}(\lambda_t). \tag{7}$$

Finally, to model the conditional distribution of non-zero measurements via a gamma distribution, we obtain the relevant parameters from the conditional mean and relative variance, i. e. with shape α and rate β given by

$$\alpha = \frac{\mathbb{E}\left[\hat{\Lambda}_t \middle| \hat{\Lambda}_t > 0\right]^2}{\text{Var}\left[\hat{\Lambda}_t \middle| \hat{\Lambda}_t > 0\right]} = \frac{1}{\nu^2(\lambda_t)}, \quad \beta = \frac{\mathbb{E}\left[\hat{\Lambda}_t \middle| \hat{\Lambda}_t > 0\right]}{\text{Var}\left[\hat{\Lambda}_t \middle| \hat{\Lambda}_t > 0\right]} = \frac{1 - p_{\text{zero}}(\lambda_t)}{\lambda_t \, \nu^2(\lambda_t)}, \tag{8}$$

which corresponds to a mean of $\mu = \frac{\lambda_t}{1 - p_{\text{zero}}(\lambda_t)}$ and coefficient of variation of $\nu = \nu(\lambda_t)$.

C Priors and epidemiological assumptions

C.1 Digital PCR parameters

The dPCR-specific observation model described in Supplementary Information B assumes a certain number of valid partitions m for each dPCR run and a conversion factor κ that maps the target concentration in the sample to the expected number of target molecules per partition in the assay. Although m and κ were known to us for our specific laboratory setup, we used broad priors for these parameters to mimic settings in which detailed laboratory information might not be available [3]. Specifically, we modeled the total number of valid partitions $m_{t,i}$ for replicate i of the sample on day t as $m_{t,i} = m_{\max}(1 - \pi^{\text{loss}}_{t,i})$, where $m_{\max}$ is the maximum number of partitions supported by the dPCR system and $\pi^{\text{loss}}_{t,i}$ is the percentage of invalid partitions (i. e. relative partition loss) for replicate i of sample t. The maximum number of partitions $m_{\max}$ is typically reported by the manufacturer of the dPCR system or chip, but it is also possible to place an uncertain prior on this parameter. To account for partition loss, we modeled $\pi^{\text{loss}}_{t,i}$ as truncated logit-normal distributed with location $\mu_{\pi^{\text{loss}}}$, scale $\sigma_{\pi^{\text{loss}}}$, and upper bound $\pi^{\text{loss}}_{\max}$. The latter allows us to account for potential quality control (QC) thresholds in the lab, which discard a dPCR run if it has too many invalid partitions. We here used a default threshold of $\pi^{\text{loss}}_{\max} = 50\%$. We also used a broad prior for the pre-PCR coefficient of variation ν_{pre}. An overview of the priors chosen is given in Supplementary Table 1.

Supplementary Table 1. Overview of priors for the dPCR-specific model. Shown are priors for the parameters of the digital PCR model described in Supplementary Information B. Normal$^+$ denotes the zero-truncated, positive normal distribution.

Parameter	Description	Prior	Details
$m_{\max}$	maximum number of partitions	Uniform$(a = 20\,000, b = 40\,000)$	accommodates dPCR systems with 20 000 to 40 000 partitions
$\pi^{\text{loss}}_{\max}$	maximum relative partition loss	$\pi^{\text{loss}}_{\max} = 0.5$	fixed to default of 50%
$\mu_{\pi^{\text{loss}}}$	logit-level expected relative partition loss	Normal$(\mu = -1.90, \sigma = 0.64)$	expected loss approx. 5 % to 30 %
$\sigma_{\pi^{\text{loss}}}$	logit-level standard deviation of partition loss	Normal$^+(\mu = 1.24, \sigma = 0.46)$	$\sigma_{\pi^{\text{loss}}} \approx [0.5, 2]$
κ	concentration conversion factor	Uniform $\left(a = 1 \times 10^{-6}, b = 1 \times 10^{-4}\right)$	$1 \times 10^{-6} \leq \kappa \leq 100 \times 10^{-6}$
ν_{pre}	pre-PCR coefficient of variation	Normal$^+(\mu = 0, \sigma = 1)$	weakly informative prior, less than 200% variation

C.2 Generation time distribution and shedding profile

Supplementary Table 2 provides an overview of the generation time distribution and shedding profiles assumed for SARS-CoV-2, Influenza A, and RSV. In our discrete-time renewal model, the generation time has a minimum of 1 day. Thus, to represent the assumed, log-normal distributed generation time of SARS-CoV-2, we discretized the probability mass between 0 and 2 to a generation time of 1 day. For the gamma distributed generation time of IAV and RSV, we used the shifted gamma distribution as proposed in Cori et al. [6], which corresponds to a gamma random variate shifted by one day. In contrast, the temporal shedding profiles for SARS-CoV-2, IAV, and RSV were discretized as $0, 1, 2, \ldots$ days after infection, since shedding can also start on the day of infection in our model. The maximum generation time G and shedding delay S were chosen heuristically to cover at least 99.5% of the probability mass of the provided prior.

To model uncertainty of the temporal shedding profile, we placed priors on the mean μ^{shed} and coefficient of variation ν^{shed} of the respective distribution. To construct the priors, we extracted parameter estimates with uncertainty information from the literature. For SARS-CoV-2, we used results from an observational study by Cavany et al. [7], where the temporal shedding profile was inferred using a Bayesian model of wastewater and case surveillance data. We used two sets of positive normal priors to account for the range of possible parameters found by the authors in a sensitivity analysis of the testing delay. These priors represent the posterior distributions of parameters for an assumed testing delay of 0 and 5 days, respectively. We then modeled μ^{shed} and ν^{shed} as weighted means of the parameters for a delay of 0 and 5 days, with the weights θ given by a Dirichlet prior. Here, we used a symmetric Dirichlet prior with concentration parameter $a = 1$, corresponding to a uniform distribution over the weight simplex. This means that we equally support parameters in between the two extremes found in the sensitivity analyses by Cavany et al. [7]. By choosing a smaller concentration parameter, this assumption can be modified towards a more isolated support of the extremes, which may be more appropriate in other cases. Moreover, this approach generalizes to more than two sets of priors, and allows for differential weighting of results from different studies through the parameters of the Dirichlet prior. We note that the estimates by Cavany et al. represent the SARS-CoV-2 wild type, however newer clinical evidence on the fecal shedding of Omicron variants is compatible with this prior. Specifically, fecal viral load measurements by

Wannigama et al. for the sub-variants XBB.1.5, EG.5.1, HV.1, J.D.1.1, BA.2.86, and JN.1 suggest a peak in shedding around 7-9 days after symptom onset, with the majority of patients shedding for at least 21 days after symptom onset [8]. Accounting for an average incubation period of 3-4 days for Omicron variants [9], this time window is compatible with our broad prior (mean of 8.0-17.7 days, 90% quantile of 17-29 days) for the shedding profile.

For IAV, we used a gamma viral load distribution from Nadeau et al. [2] that was fitted to results from a meta-analysis of human volunteer challenge studies by Carrat et al. [10], based on nasal wash samples. We used the estimated parameters and associated standard errors of the model fitted by [2] to construct positive normal priors for μ^{shed} and ν^{shed}. For RSV, we used results from a household study based on nasal swab samples by Otomaru et al. [11], and constructed normal priors for μ^{shed} and ν^{shed} based on the means and credible intervals of parameters of a gamma distribution fitted by the authors. We note that when constructing priors for the mean and coefficient of variation of the gamma distribution, we were not able to account for the correlation between the original shape and rate parameters estimated by the respective studies. Moreover, our shedding profiles for IAV and RSV reflect the temporal distribution of viral concentrations measured from patient samples. This is based on the assumption that the overall material load shed is constant over the course of an infection and thus only serves as a scaling factor that does not affect the relative shedding profile. For other pathogens, such as gastrointestinal viruses with variation in the amount of fecal excretion over the course of an infection, material loads might need to be incorporated when estimating the distribution of loads over time.

C.3 Total shedding load per infection

We obtained site-specific estimates for the average detectable load per infection by relating a catchment's loads in the wastewater to the number of infections in that catchment. To estimate the number of infections in a catchment, we used data from the Swiss Sentinel Surveillance System (Sentinella). The data is publicly available from the Infectious Diseases Dashboard by the Swiss Federal Office of Public Health [16]. This system is based on voluntary contributions from approximately 170 enrolled general practitioners, internists and paediatricians, who report information on consultations of patients with acute respiratory infection (ARI, "acute onset of illness with cough, sore throat, shortness of breath or rhinitis AND of infectious origin, as judged by a physician") or influenza-like

Supplementary Table 2. Overview of assumed generation time distributions and shedding profiles. Note that for easier comparison, we here parameterize the log-normal and gamma distribution by their mean μ and standard deviation σ or coefficient of variation ν. Gamma$^{\rightarrow}$ denotes the shifted gamma distribution, as proposed in Cori et al. [6]. If X is shifted gamma distributed with some parameters, then $X-1$ is gamma distributed with identical parameters. $\boldsymbol{\theta}$, $\boldsymbol{\mu}^{\text{shed}}$, and $\boldsymbol{\nu}^{\text{shed}}$ denote vectors. Note that the generation time distributions and shedding profiles are discretized as $\tau^{\text{gen}} = (\tau_1^{\text{gen}}, \tau_2^{\text{gen}}, \dots, \tau_G^{\text{gen}})$ and $\tau^{\text{shed}} = (\tau_0^{\text{shed}}, \tau_1^{\text{shed}}, \dots, \tau_S^{\text{shed}})$ in our model. N^+ denotes the zero-truncated, positive normal distribution.

**Distribution**	**Pathogen**	**Assumption**	**Details**
Generation time	SARS-CoV-2	Log-Normal$(\mu = 3.0, \sigma = 1.5)$	Park et al. [12], Omicron generation time, growth-rate corrected within-household estimate
	IAV	Gamma$^{\rightarrow}(\mu = 2.6, \sigma = 1.7)$	te Beest et al. [13]
	RSV	Gamma$^{\rightarrow}(\mu = 7.5, \sigma = 2.1)$	Vink et al. [14], serial interval (originally normal distributed), data from Crowcroft et al. [15]
Shedding profile	SARS-CoV-2	Gamma$(\mu = \boldsymbol{\theta} \cdot \boldsymbol{\mu}^{\text{shed}}, \nu = \boldsymbol{\theta} \cdot \boldsymbol{\nu}^{\text{shed}})$<br>$\boldsymbol{\mu}^{\text{shed}} \sim \mathcal{N}^+\left((9.16, 15.69)^T, \text{diag}(0.83^2, 0.79^2)\right)$<br>$\boldsymbol{\nu}^{\text{shed}} \sim \mathcal{N}^+\left((0.87, 0.53)^T, \text{diag}(0.06^2, 0.04^2)\right)$<br>$\boldsymbol{\theta} \sim \text{Dirichlet}(1, 1)$	Cavany et al. [7], observational study, range of sensitivity analysis assuming a testing delay of 0–5 days
	IAV	Gamma$(\mu = \mu^{\text{shed}}, \nu = \nu^{\text{shed}})$<br>$\mu^{\text{shed}} \sim \mathcal{N}^+\left(2.50, 0.19^2\right)$<br>$\nu^{\text{shed}} \sim \mathcal{N}^+\left(0.34, 0.01^2\right)$	Nadeau et al. [2], parametric estimate using empirical distribution from Carrat et al. [10], nasal washing
	RSV	Gamma$(\mu = \mu^{\text{shed}}, \nu = \nu^{\text{shed}})$<br>$\mu^{\text{shed}} \sim \mathcal{N}^+\left(6.76, 1.17^2\right)$<br>$\nu^{\text{shed}} \sim \mathcal{N}^+\left(0.35, 0.05^2\right)$	Otomaru et al. [11], nasal swabs

illness (ILI, "sudden onset of high fever (>38°C) and cough or sore throat") in medical practices and during home visits. Nasopharyngeal swabs are taken from a subset of consulted patients with acute respiratory infection, and tested at the National Reference Centre for Influenza (NRCI) for various respiratory pathogens, including SARS-CoV-2, Influenza A and B virus, and RSV. From the ratio of the number of positive tests for a given pathogen j to the total number of tests, the weekly proportion p_w^{j} of consulted patients with ARI that are infected with the pathogen can be estimated. In addition, using the reported numbers of ARI consultations, the total number of consultations, reported age and sex distributions of consulted patients, and general population statistics in Switzerland, the Federal Office of Public Health (FOPH) projects a weekly incidence of ARI-related consultations c_w^{ARI} for the Swiss population. Finally, we used estimates based on syndromic reports by the participatory surveillance system "Grippenet Suisse" [17] for the incidence of influenza-like illness infections in Switzerland as a conservative proxy (subset of ARI infections) for the under-ascertainment of acute respiratory infections in Switzerland. For each flu season, we estimated the underreporting factor as $\rho = \frac{\sum_w I_w^{\mathrm{Grippenet}}}{\sum_w c_w^{\mathrm{ARI}}}$, where $I_w^{\mathrm{Grippenet}}$ is the estimated ILI incidence from Grippenet and c_w^{ARI} the number of ARI consultations in week w of the season, respectively. Assuming that consultation is independent of the probability of infection with a pathogen, we then estimated the total number of new infections with pathogen j in week w in the Swiss population as $I_w^j = p_w^{\mathrm{j}} \times c_w^{\mathrm{ARI}} \times \rho$. Using the population size n_k of a catchment k relative to the Swiss population size $n = 8\,770\,000$, we obtained a crude estimate of the weekly number of new infections with the pathogen in that catchment, $I_{w,k}^j = I_w^j \times \frac{n_k}{n}$. For each week, we divided the sum of measured loads on all days $t \in T_w$ of the week by the weekly number of infections to obtain a weekly estimate of the load per infection $\hat{\mu}_w^{\mathrm{load}} = \frac{\sum_{t \in T_w} c_t \times \mathrm{flow}_t}{I_{w,k}^j}$. We found the estimated weekly load per infection to be relatively stable over time and during seasonal waves. For our model parameter μ^{load}, we used the median of weekly estimated average loads per infection from the first weeks of the flu season before December 1.

C.4 Outliers

The parameter ϵ_t represents spikes in sample concentrations that strongly deviate from the concentration predicted by our model under the assumption of uniform mixing. We here focus on positive outliers, which we have historically observed to occur at an average frequency of several times per year across different pathogens and which can take extreme values corresponding to the

shedding by thousands of infected individuals [18]. While there are various potential explanations for such spikes, including unrepresentative sampling and sewer-level effects [19], evidence on the exact mechanisms leading to outlier observations is still limited. Moreover, the sparsity of outliers renders identification of predictors for outlier frequency and intensity highly challenging. We thus employ a phenomenological approach in which we parameterize ϵ_t using a fixed but broad outlier distribution that accommodates outliers across multiple scales. Specifically, we use a generalized extreme value distribution, i. e.

$$\epsilon_t \sim \mathrm{GEV}(k\mu^\epsilon, k\sigma^\epsilon, \xi^\epsilon). \tag{9}$$

with scaling factor k, location $\mu^\epsilon = 0$, scale $\sigma^\epsilon = 2 \times 10^{-8}$, and shape $\xi^\epsilon = 4$. This yields a Type II/Frèchet extreme value distribution with a heavy right tail. As a scaling factor, we chose $k = \frac{\mu^{\text{load}}}{\text{med(flow)}}$ to approximate the expected sample concentration corresponding to only 1 infection in the catchment. The resulting prior assigns less than 1% probability to spikes larger than 1 infection (≈ 3 per year), approximately 0.5% to spikes larger than 10 infections ($\approx$ 1–2 per year), approximately 0.3% to spikes larger than 100 infections (≈ 1 per year), and still over 0.1% to spikes larger than 1000 infections (≈ 0.5 per year). These probabilities approximately match the observed frequency of outliers in our historical data (Supplementary Fig. 4). We note that our choice of an additive rather than a multiplicative outlier component is primarily motivated by reasons of identifiability. As sample concentrations in digital PCR approach zero, the coefficient of variation of measurements grows without bound (see Supplementary Information B.2). At low concentrations, this increased relative measurement error becomes practically indistinguishable from a multiplicative outlier component. For example, a 100% deviation from the expected concentration would qualify as an outlier at high concentrations, but is consistent with typical dPCR measurement error at very low concentrations. In contrast, by modeling outliers as additive deviations from the expected concentration, we can apply a consistent prior across epidemic phases that matches the calibrated load per infection.

While strong negative deviations from the expected concentration are also observed in our data, these observations do not necessarily constitute outliers. Specifically, low concentrations can be caused by dilution of genetic material at high flows, e. g. during extreme rainfall. Moreover, as measurement error in digital PCR approaches a near-constant coefficient of variation at high sample

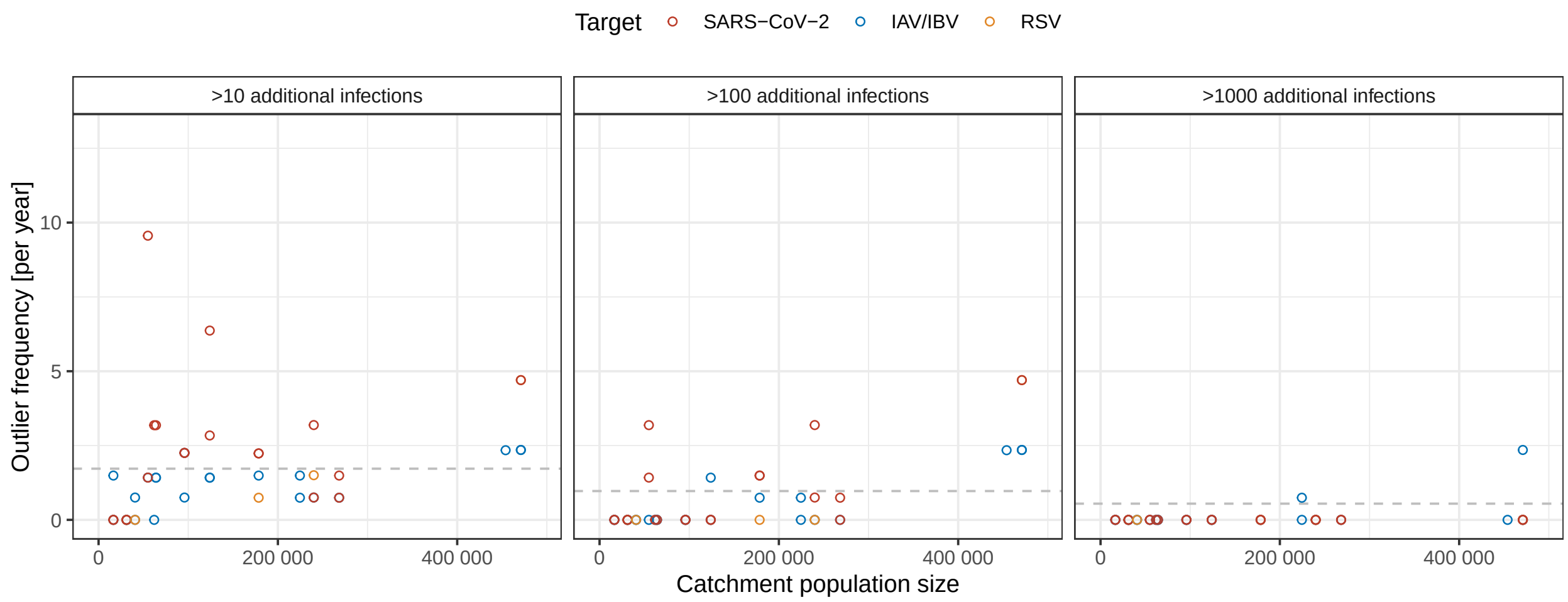

Supplementary Fig. 4. Frequency and size of observed outliers. Shown are observed yearly frequencies of outliers across pathogen targets and locations by size of the catchment population. Outliers were labeled by human experts and grouped by size, corresponding to the number of additional infections implied. Additional infections were estimated using the calibrated detectable load per infection (Supplementary Information C.3) and the deviation of the outlier concentration from a robust, retrospective trend estimate [18]. Dashed horizontal lines show the expected outlier frequency implied by our extreme value distribution prior. Source data are provided as a Source Data file.

concentrations, large absolute deviations from the expected concentration are not unusual. By accounting for these factors in our model, we find that low concentrations observed in our data were sufficiently captured without a negative outlier component. If negative outliers were to be included in the model, however, this would likely require a separate modeling component, given that positive and negative outliers may be caused by vastly different mechanisms. For example, settling-resuspension phenomena may have non-linear effects on sample concentrations (i. e. gradual settling, rapid resuspension caused by an external event), potentially favoring positive outliers [20].

C.5 Transmission dynamics

In the following, we provide further details on the priors used for the reproduction number R_t and the seeding phase of the infection trajectory. As described in the main text, we smoothed R_t using two approximate Gaussian processes with long- and short-term kernels, i. e.

$$\alpha \sim \mathcal{GP}(1, k_\nu(\tau; \ell_\alpha, \sigma_\alpha^2)),\ \delta \sim \mathcal{GP}(0, k_\nu(\tau; \ell_\delta, \sigma_\delta^2)), \tag{10}$$

see Supplementary Information D for details on the approximation. By decomposing R_t into long- and short-term components, we obtain desirable behavior in real-time estimation, where transmission

dynamics are only partially informed by wastewater data due to shedding delays. In the absence of signal, the short-term deviation component reverts to its mean of zero, causing R_t to follow the long-term trend α_t. Although α_t also reverts to its prior mean of $R_t = 1$ over long time scales, this effect is negligible for real-time estimation and short-term forecasts, as the long kernel length scale enforces strong correlation between consecutive R_t values. Thus, in expectation, R_t remains at its current level near the present, representing a minimally informed assumption of stable transmission dynamics when wastewater data provide no contrary evidence. Moreover, through the use of an inverse softplus link $g(R_t) = \alpha_t + \delta_t$, we obtain a prior for R_t that is approximately symmetric with respect to changes in transmission. This is not given for other popular link functions such as the logarithmic function, which e. g. favors a decrease from $R_t = 2$ to $R_t = 1$ over an increase from $R_t = 1$ to $R_t = 2$. In contrast, the softplus function closely approximates the identity function for practically relevant values of R_t when a high scaling factor ($k = 4$) is used.

For the seeding phase, during which R_t cannot be calculated, we let the expected number of new infections ι_t follow an exponential growth process, where ι_1 is the initial number of infections. We used a broad prior for ι_1, informed by a rough empirical estimate of the initial number of infections to ensure that the seeding phase is modeled on the right order of magnitude. Specifically, we used the supplied wastewater measurements and shedding assumptions to approximately back-calculate the initial number of infections and then constructed a zero-truncated, positive normal prior with parameters chosen such that the 5% and 95% of the distribution correspond approximately to 1/10 and 10 times the empirical estimate of the number of infections. The exponential growth rate r_t during the seeding phase was modeled via a backward-in-time random walk with starting value r_{G+1} and standard deviation σ_r, i. e.

$$r_t | r_{t+1} \sim \mathcal{N}(r_{t+1}, {\sigma_r}^2) \mid 1 < t \leq G. \tag{11}$$

We obtain r_{G+1} from the reproduction number R_{G+1} using the relationship $R_{G+1}(\sum_{s=1}^{G} \tau_s^{\text{gen}} e^{-s r_{G+1}}) = 1$, which we solve numerically for r_{G+1} via root-finding with the Newton–Raphson method. For σ_r, we used a zero-truncated positive normal prior, i. e. $\sigma_r \sim \text{Normal}^+(\mu = 0, \sigma = 0.01)$, allowing the growth rate to vary by up to $\approx \pm 4$ percentage points per day. This approach models the initial R_t values as a continuation of an exponential growth

trend, while allowing us to express the expected growth rates during the seeding phase via a prior for the initial reproduction number R_{G+1}.

To compute the growth rate for $t > G$, we used the estimator

$$r_t = \frac{d \log \Lambda_{t+\bar{\tau}}}{dt}, \quad \Lambda_t = \sum_{s=1}^{G} \tau_s^{\text{gen}} \iota_{t-s}, \tag{12}$$

which corresponds to the logarithmic derivative of the total expected infectiousness Λ_t, shifted backward in time by the mean generation time $\bar{\tau}$. This estimator was proposed by Parag et al. [21] as a more stable alternative to the logarithmic derivative of new infections.

D Estimation

D.1 Parameters

Supplementary Table 3 provides an overview of all parameters estimated by fitting our model to observed concentration measurements c. Note that for our model implementation, we reparameterized certain model components to achieve better sampling performance under Hamiltonian Monte Carlo sampling (see Supplementary Information D.2). As a result, some of the parameters described in Supplementary Table 3 are not sampled directly; instead, they are computed based on transformations of simpler variables. The model remains semantically equivalent, however.

D.2 Implementation

We implemented our Bayesian hierarchical model in the probabilistic programming language "stan" [22] to allow sampling from the posterior distribution of parameters via Hamiltonian Monte Carlo. As sampling of discrete parameters is not supported in stan, we used continuous approximations for the realized number of new infections, where I_t is modeled as zero-truncated, positive normal distributed with mean and standard deviation parameters matching those of a Poisson variate [23–26], i. e.

$$I_t | \iota_t \sim \text{Normal}^+(\mu = \iota_t, \sigma = \sqrt{\iota_t}). \tag{13}$$

To improve sampling efficiency, we also approximated the distribution for $\zeta_t \sim \text{Gamma}(\alpha = \frac{I_t}{\nu_\zeta^2}, \beta = \frac{1}{\nu_\zeta^2})$ using a zero-truncated, positive normal distribution with matching mean and standard deviation parameters when the shape parameter of the gamma distribution was $\alpha \geq 30$.

Supplementary Table 3. Overview of estimated parameters. The estimated model parameters together define the transmission dynamics (R_t) and the infection, shedding, and measurement process. Non-hierarchical parameters have independent priors, while hierarchical parameters have priors that depend on other parameters.

Parameter	Description	Hierarchical
**Gaussian process (GP) parameters for R_t**		
ℓ_α	Length scale of the long-term trend GP	no
ℓ_δ	Length scale of the short-term deviation GP	no
σ_α	Kernel magnitude of the long-term trend GP	no
σ_δ	Kernel magnitude of the short-term deviation GP	no
α_t	Realized daily value of the long-term trend	yes
δ_t	Realized daily value of the short-term deviation	yes
**Infections θ^{inf}**		
ι_1	Initial expected number of new infections (seeding phase)	no
σ_r	Standard deviation of random walk for r_t (seeding phase)	no
r_t	Realized daily growth rate of new infections (seeding phase)	yes
I_t	Realized daily number of new infections	yes
**Shedding θ^{shed}**		
μ^{shed}	Mean of the temporal shedding profile	no
ν^{shed}	Coefficient of variation of the temporal shedding profile	no
ζ_t	Number of new infects weighted by shedding intensity	yes
**Measurement θ^{meas}**		
ν_{pre}	Pre-PCR coefficient of variation	no
κ	Concentration conversion factor	no
$m_{\max}$	Maximum number of partitions in the assay	no
$\pi_{t,i}^{\text{loss}}$	Realized proportion of invalid partitions (replicate i of sample t)	yes
$\mu_{\pi^{\text{loss}}}$	Location parameter of the distribution for $\pi_{t,i}^{\text{loss}}$	no
$\sigma_{\pi^{\text{loss}}}$	Scale parameter of the distribution for $\pi_{t,i}^{\text{loss}}$	no
ϵ_t	Realized daily outlier component	no

To sample from the Gaussian processes used as smoothing priors for R_t, we implemented a basis function approximation using Laplace eigenfunctions described in Riutort-Mayol et al. [27,28]. This approach was implemented in a similar fashion by Abbott et al. for estimating R_t from case count data [29]. In our implementation, we used a boundary factor of $c = 3$, which is higher than the minimum recommended by Riutort-Mayol et al. to ensure accurate real-time estimates, i. e. also near the boundaries. Moreover, based on recommendations in Riutort-Mayol et al., we used $m = 3.42\,c\,\frac{S}{l}$ different basis functions, where l is the length scale of the Gaussian process (we used the 5% quantile of the length scale prior for a conservative result), and S is the maximum absolute value of the zero-centered input space, which is $\frac{n-1}{2}$, where n is the number of R_t time steps modeled. We note that in an earlier draft of this manuscript, the parameterization of the noise in the Gaussian process components had an implementation error, which effectively let the expected prior variance of the process correspond to that of a half-normal rather than a normal distribution, i. e. $\mathbb{E}[\sigma^2] - \frac{2}{\pi}(\mathbb{E}[\sigma])^2$ instead of $\mathbb{E}[\sigma^2]$. The error has been fixed and the respective priors adjusted.

We generally used non-centered parameterizations of normally distributed nuisance parameters such as random walk increments. Where possible, we reparameterized parameters on the logarithmic scale to improve numerical stability.

D.3 Sampling and diagnostics

For sampling, we ran the No-U-Turn Sampler (NUTS) implemented in cmdstan version 2.34.1 [22] using 4 parallel chains with 1000 warm-up and 2500 sampling iterations each, a maximum tree depth of 15, an initial step size of 0.01, and an adaptation target acceptance statistic of 0.99. For each fitted model, we assessed several diagnostics of the NUTS sampler, specifically the number of divergent transitions [30] and the Bayesian fraction of missing information [31]. The majority of fitted models had no divergent transitions (Supplementary Fig. 5). We found divergent transitions for model fits on some days, in particular when fitting models during the off-season 2022/23, which included a two-week processing break due to the switch from a four-plex to a six-plex assay. However, we did not observe a noticeable difference between transmission dynamic estimates on days with and without divergent transitions. This suggests that small numbers of divergent transitions might be acceptable for our application [32], but if highly accurate estimates are important, we suggest running the model with a higher adaptation target acceptance statistic. The Bayesian fraction of missing information was consistently above the default threshold of 0.2 across model fits (Supplementary Fig. 6). We also checked the daily R_t estimates via the Gelman-Rubin convergence diagnostic ($\hat{R} < 1.05$) [33] and for sufficient effective sample sizes (ESS > 400) [34]. $\hat{R}$ values were below 1.05 for most model fits, although there were some outliers with higher $\hat{R}$ values on a small number of dates (Supplementary Fig. 7). Effective sample sizes were consistently above 400 for both the bulk and tail of the distribution, again with the exception of a small number of dates that had a reduced bulk effective sample size (Supplementary Figs. 8 and 9). Per-chain runtimes increased approximately linearly with the length of the modeled time window, with an average slope of 3.2 min per week of data. For the given chain length (1000 warm-up and 2500 sampling iterations), runtimes ranged from an average of 12.6 min per chain for 4 weeks of data to 85.0 min per chain for 26 weeks of data (Supplementary Fig. 10).

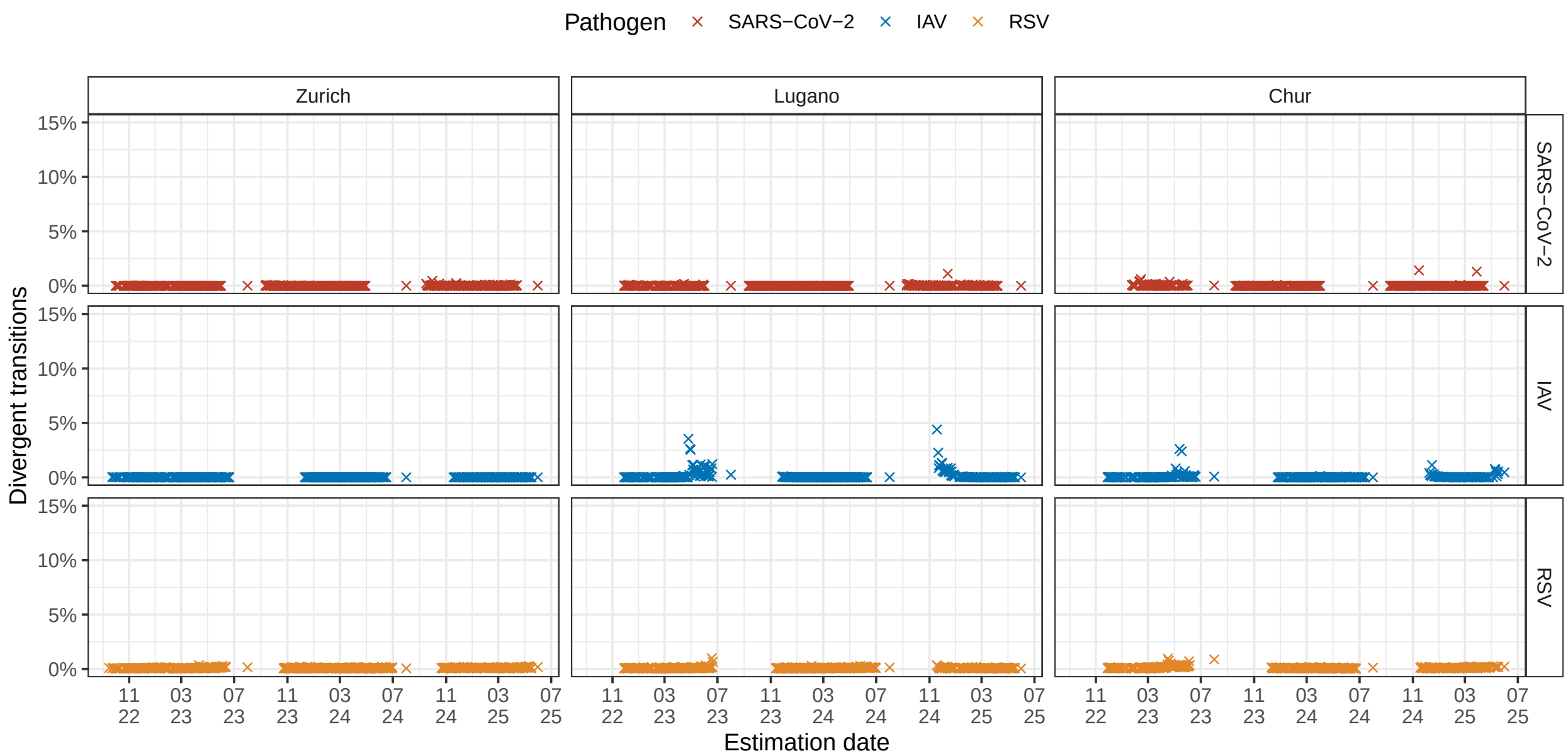

Supplementary Fig. 5. Percentage of divergent transitions per fitted model. Shown is the share of divergent transitions out of 10 000 sampling iterations for all real-time model fits for SARS-CoV-2, influenza A virus (IAV), and respiratory syncytial virus (RSV), across catchments and seasons. Source data are provided as a Source Data file.

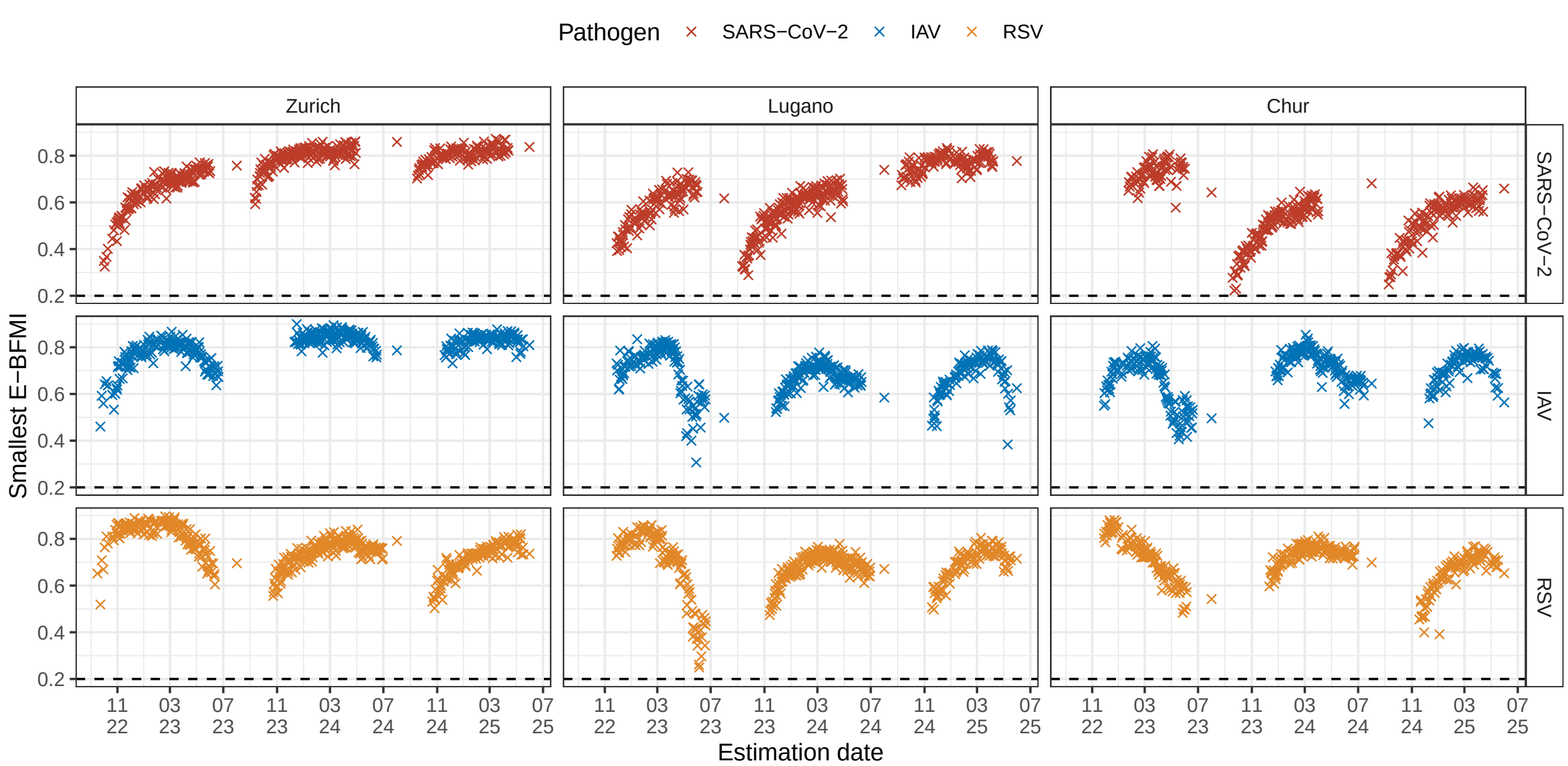

Supplementary Fig. 6. Smallest E-BFMI per fitted model. Shown is the smallest E-BFMI out of 4 chains for all real-time model fits for SARS-CoV-2, influenza A virus (IAV), and respiratory syncytial virus (RSV), across catchments and seasons. E-BFMI values above 0.2 are considered sufficient. Source data are provided as a Source Data file.

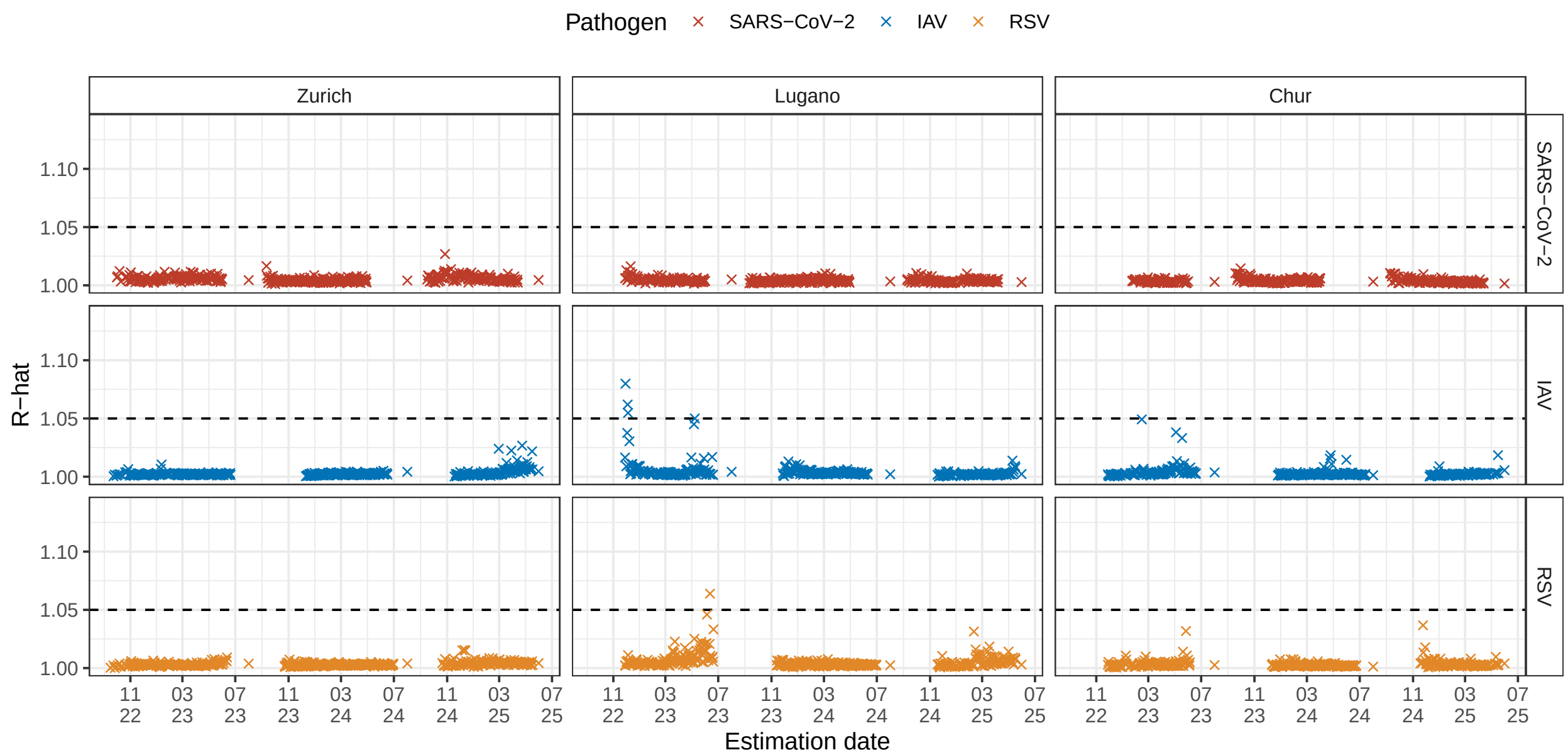

Supplementary Fig. 7. Gelman-Rubin convergence diagnostic ($\hat{R}$) per fitted model. Shown is the average $\hat{R}$ value of effective reproduction number (R_t) estimates for all real-time model fits for SARS-CoV-2, influenza A virus (IAV), and respiratory syncytial virus (RSV), across catchments and seasons. Values of $\hat{R} < 1.05$ are considered sufficient. Source data are provided as a Source Data file.

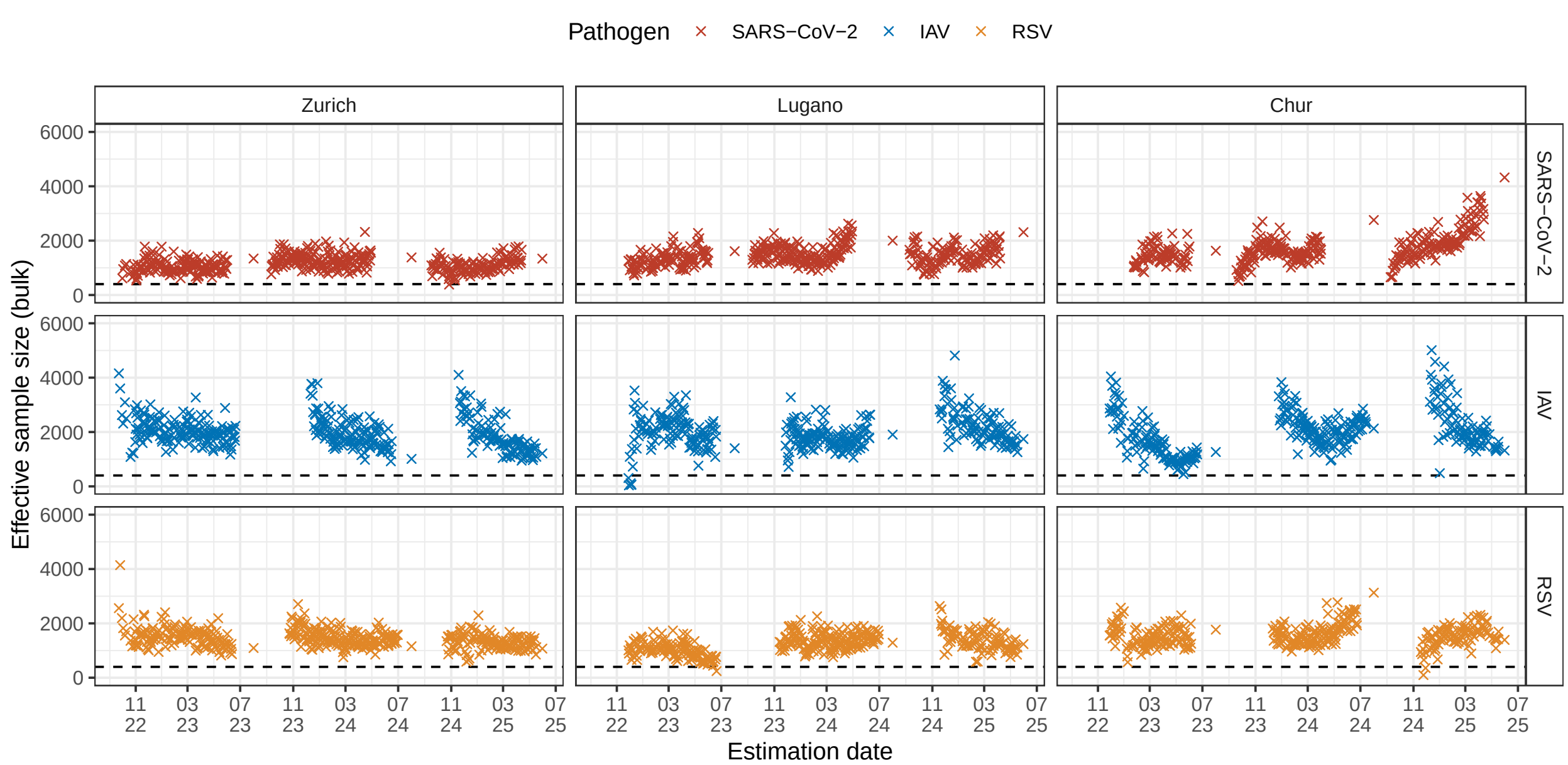

Supplementary Fig. 8. Bulk effective sample size per fitted model. Shown is the average bulk effective sample size of effective reproduction number (R_t) estimates for all real-time model fits for SARS-CoV-2, influenza A virus (IAV), and respiratory syncytial virus (RSV), across catchments and seasons. An effective sample size above 100 per Markov chain is considered sufficient. Source data are provided as a Source Data file.

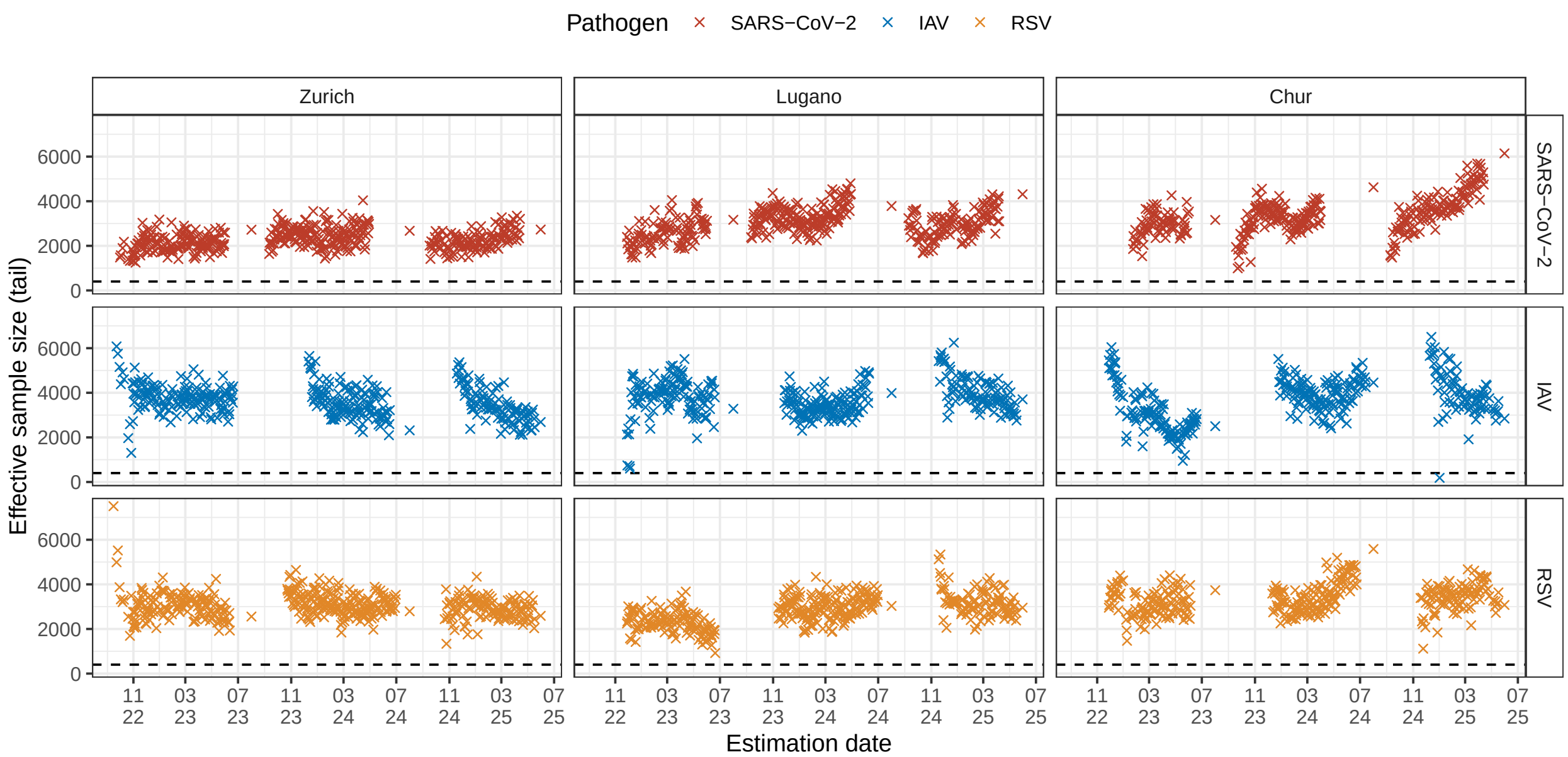

Supplementary Fig. 9. Tail effective sample size per fitted model. Shown is the average tail effective sample size of effective reproduction number (R_t) estimates for all real-time model fits for SARS-CoV-2, influenza A virus (IAV), and respiratory syncytial virus (RSV), across catchments and seasons. An effective sample size above 100 per Markov chain is considered sufficient. Source data are provided as a Source Data file.

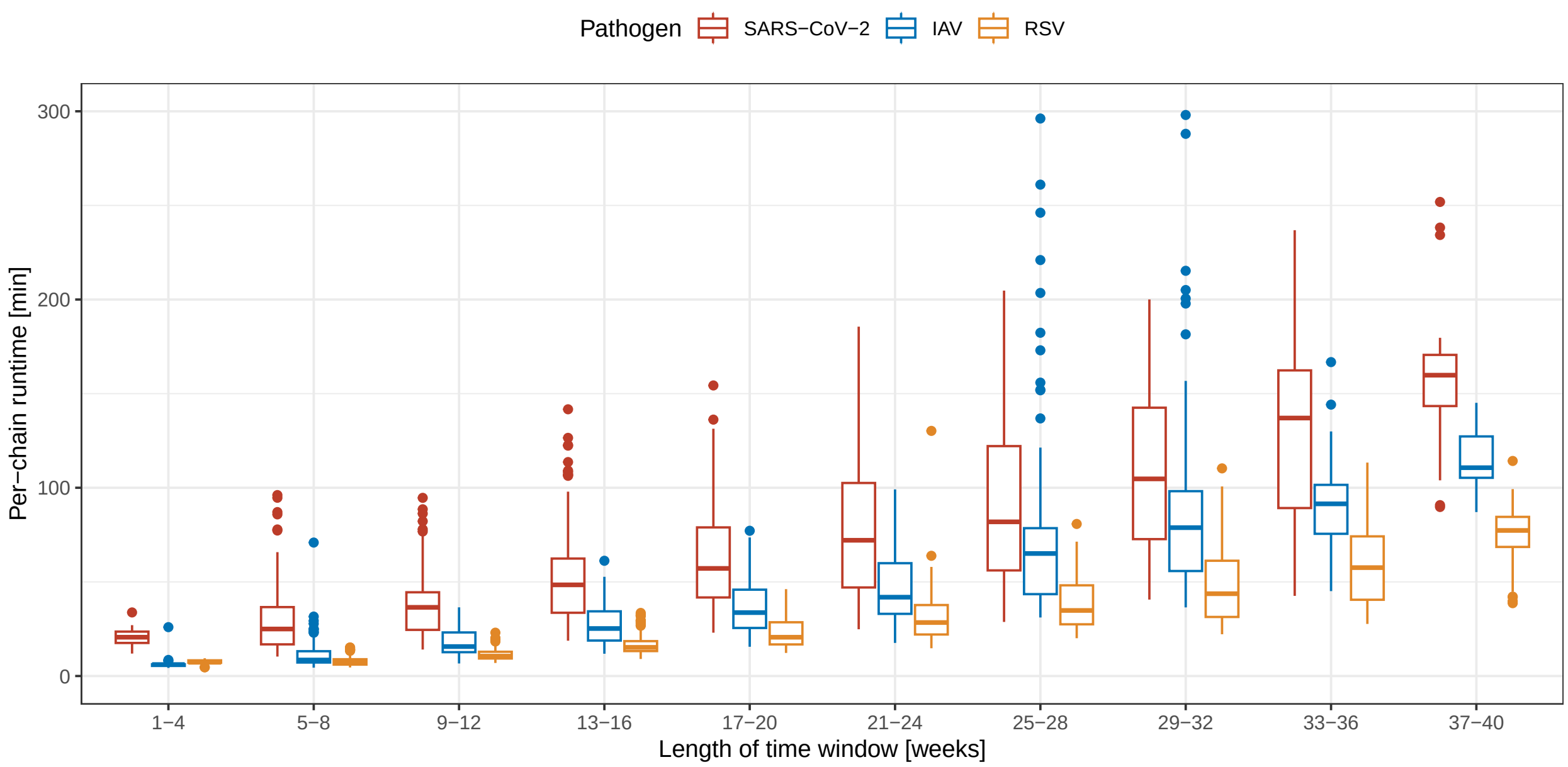

Supplementary Fig. 10. Average chain runtime per fitted model. Shown is the relationship between the length of the modeled time window and the average runtime per chain across all real-time model fits for SARS-CoV-2, influenza A virus (IAV), and respiratory syncytial virus (RSV) for the different catchments and seasons. Box plots show the median (centre line), 25th and 75th percentiles (box bounds), and 1.5x the interquartile range (whiskers). Values beyond the whiskers are plotted as individual points (outliers). Models were run on HPC nodes with an AMD EPYC 7763 CPU or similar, and are comparable in runtime to a modern laptop. Chains were run with 1000 warm-up and 2500 sampling iterations each. Source data are provided as a Source Data file.

E Sensitivity analyses

E.1 Flow data

Supplementary Figs. 11 and 12 show a comparison of models fitted with and without daily flow data to measurements of SARS-CoV-2 concentrations at the WWTPs of Zurich and Lugano. When no daily flow data was used, we assumed a constant flow equal to the median value. As can be seen, the estimated wave for SARS-CoV-2 in Zurich had a lower peak and a smaller R_t when no flow data was used, because the increased flow during the winter season was not taken into account (Supplementary Fig. 11). In contrast, the estimated wave in Lugano had a higher peak and a higher R_t when no flow data was used, as Lugano had less flow during the winter season (Supplementary Fig. 12).

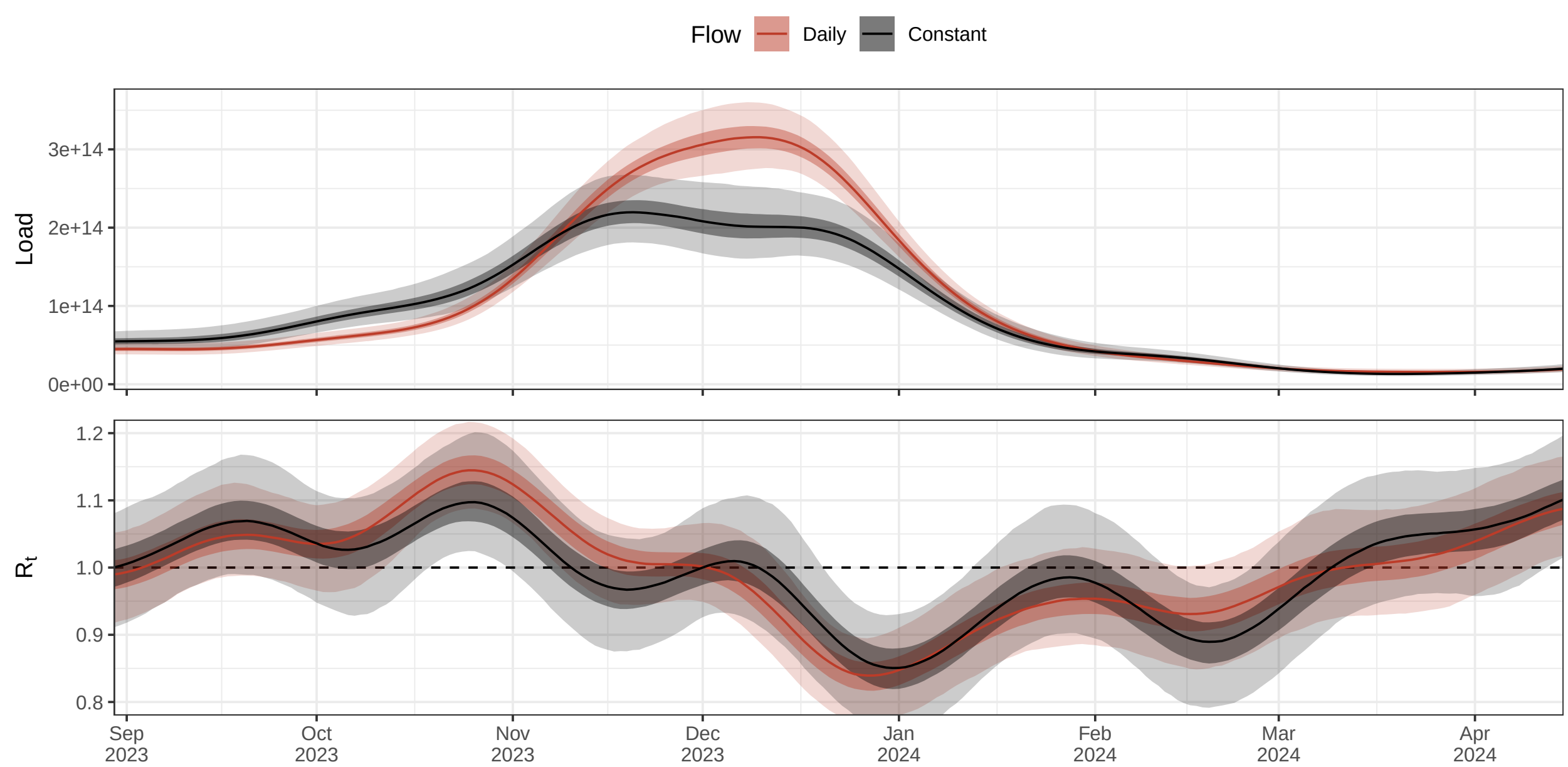

Supplementary Fig. 11. Comparison of SARS-CoV-2 wave in Zurich estimated with and without flow data. Shown is the estimated total daily load in wastewater (top) and the effective reproduction number (bottom) for SARS-CoV-2 during the winter season 2023/24 in Zurich, estimated i) using daily flow data (red) and ii) assuming a constant flow (black). Lines indicate the posterior median and dark and light bands the 50% and 95% credible intervals of the marginal posterior distribution, respectively. Source data are provided as a Source Data file.

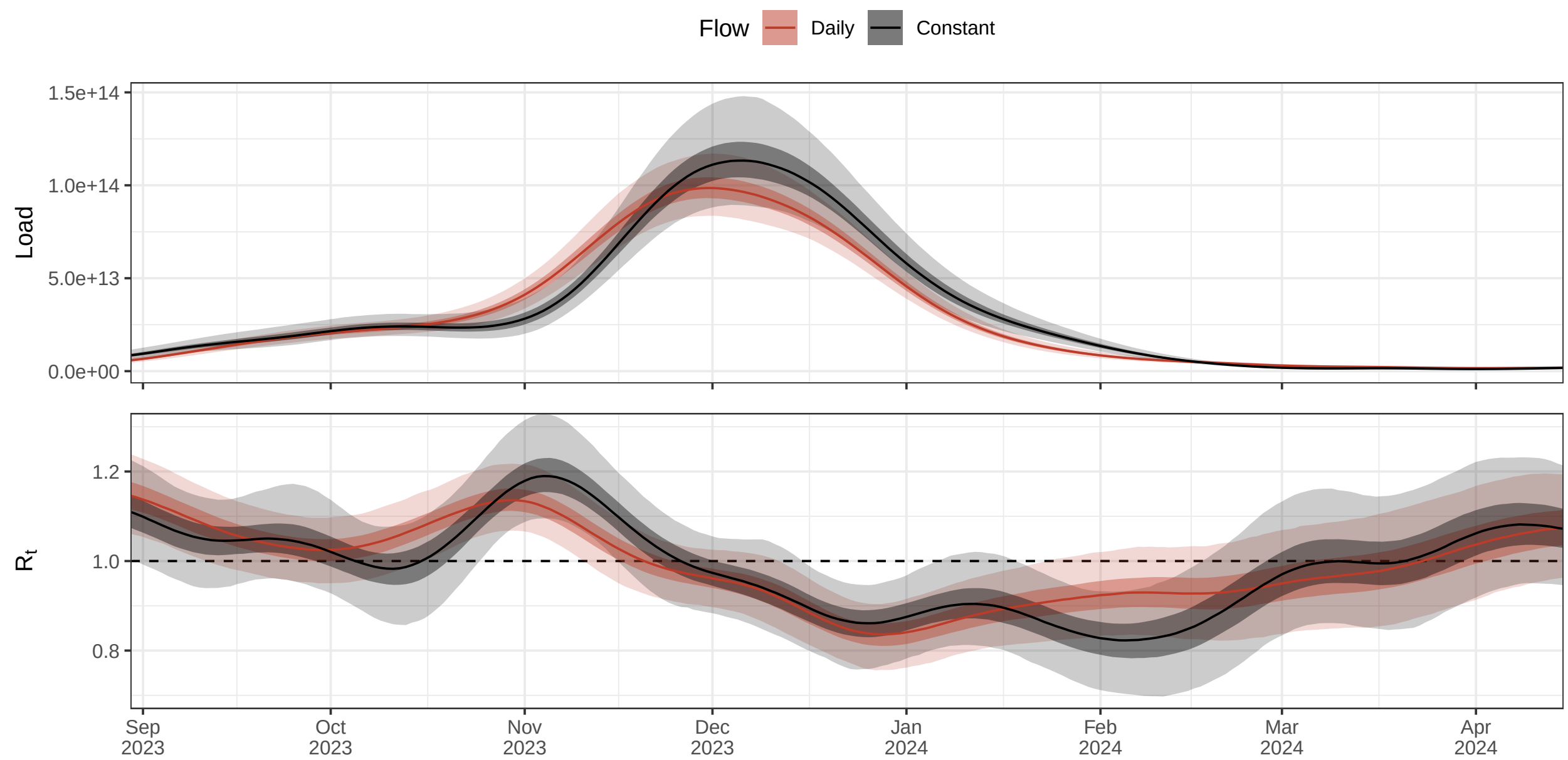

Supplementary Fig. 12. Comparison of SARS-CoV-2 wave in Lugano estimated with and without flow data. Shown is the estimated total daily load in wastewater (top) and the effective reproduction number (bottom) for SARS-CoV-2 during the winter season 2023/24 in Lugano, estimated i) using daily flow data (red) and ii) assuming a constant flow (black). Lines indicate the posterior median and dark and light bands the 50% and 95% credible intervals of the marginal posterior distribution, respectively. Source data are provided as a Source Data file.

E.2 Outlier detection

Supplementary Figs. 13 and 14 show a comparison of effective reproduction number estimates obtained when either including outliers, as in the main text, or removing them before model fitting. As can be seen, both for SARS-CoV-2 in Chur and for RSV in Zurich, the removal of outliers had no relevant effect on the estimated R_t trajectory. This suggests that our modeling of occasional, strong spikes in concentrations can successfully identify and isolate individual outliers, reducing their effect on the estimated transmission dynamics to near zero.

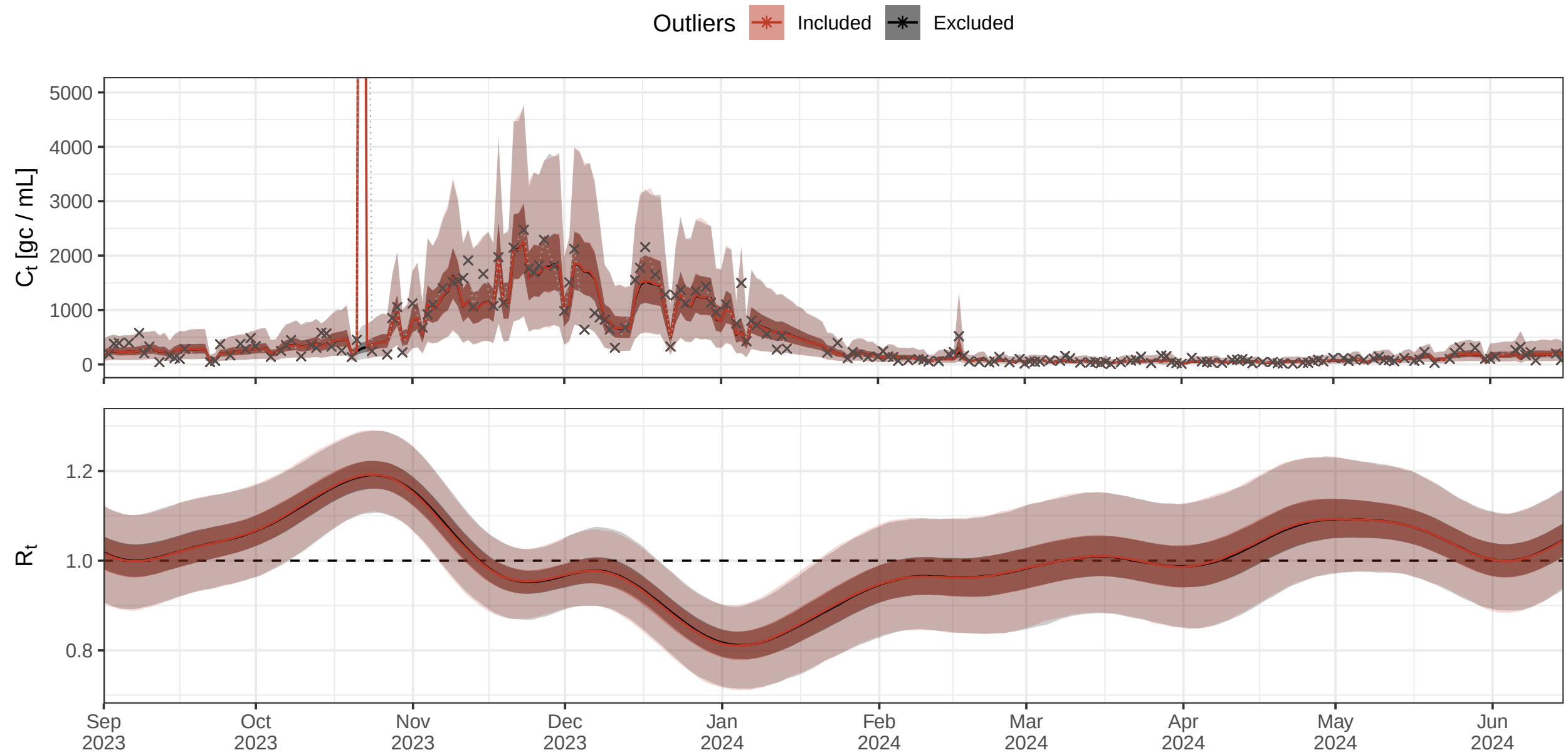

Supplementary Fig. 13. Robustness of estimated transmission dynamics of SARS-CoV-2 in Chur to outlier measurements. Shown is the model fit of the measured concentration in wastewater (top) and the estimated effective reproduction number (bottom) for SARS-CoV-2 during the winter season 2023/24 in Chur, estimated i) with outliers included, as in the main text (orange) and ii) with outliers removed (black). Lines indicate the posterior median and dark and light bands the 50% and 95% credible intervals of the marginal posterior distribution, respectively. Source data are provided as a Source Data file.

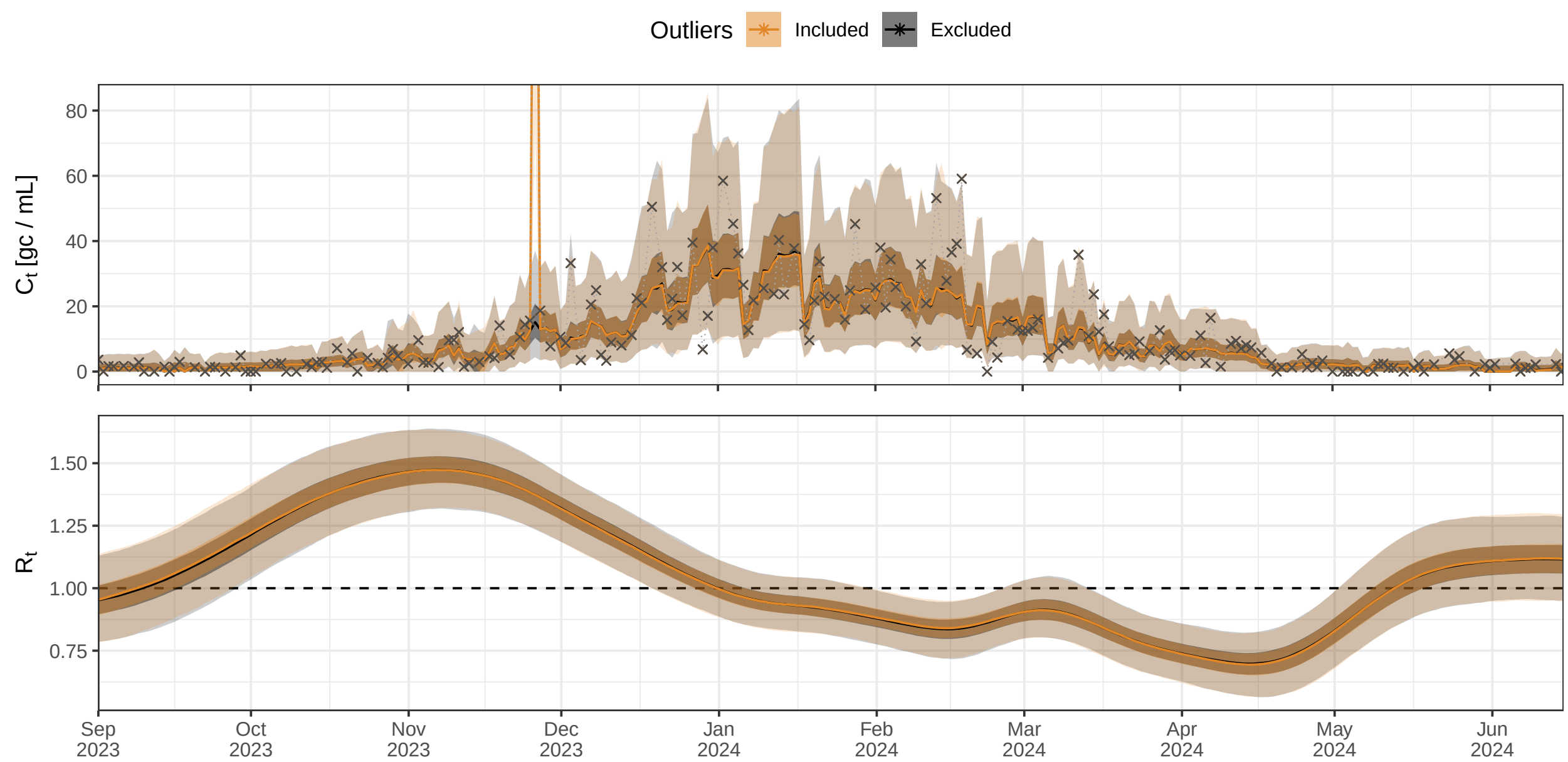

Supplementary Fig. 14. Robustness of estimated transmission dynamics of RSV in Zurich to outlier measurements. Shown is the model fit of the measured concentration in wastewater (top) and the estimated effective reproduction number (bottom) for RSV during the winter season 2023/24 in Zurich, estimated i) with outliers included, as in the main text (orange) and ii) with outliers removed (black). Lines indicate the posterior median and dark and light bands the 50% and 95% credible intervals of the marginal posterior distribution, respectively. Source data are provided as a Source Data file.

E.3 Shedding load

E.3.1 Average detectable shedding load per infection

In our main analysis, we calibrated the average shedding load per infection using data from Sentinel and participatory surveillance in Switzerland (Supplementary Information C.3). For comparison, Supplementary Figs. 15 to 17 show R_t estimates based on a simpler calibration, where we assumed that the load detected at the start of each season (average of first three consecutive positive samples) corresponds to the total load shed by 0.01% of the catchment population. For most catchments and targets, the resulting R_t estimates were practically identical to the main analysis. Notable exceptions were estimates for SARS-CoV-2 at the catchments of Lugano and Chur, which were more uncertain than in the main analysis, particularly during the winter seasons 2022/23 and 2024/25. We also conducted a systematic sensitivity analysis of the assumed total detectable load per infection using the example of SARS-CoV-2 in Zurich during the winter season 2023/24 (Supplementary Fig. 18). Here we find that estimated transmission parameters such as the effective reproduction number are in principle invariant to the assumed load per infection, which acts as a constant scaling factor for the number of new infections in the population. We therefore obtain practically identical estimates in most settings. However, as our model also accounts for stochasticity in the infection process, estimates of R_t become more uncertain when the infection incidence is very low. In the example shown in Supplementary Fig. 18, we find a notable difference in uncertainty when the estimated peak incidence is 20 instead of 2000, which corresponds to a regime with few infections that is considerably influenced by chance events. Overall, this means that misspecification of the load per infection is only relevant if the true incidence is very low (in this case, an underestimation of the load per infection could lead to an underestimation of R_t uncertainty) or if the true incidence is high but the load per infection is massively overestimated (in this case, the estimated incidence would be too low, leading to overly uncertain R_t estimates). Outside these cases, the assumed load per infection has little effect on R_t estimates. Moreover, as demonstrated in Supplementary Fig. 19, this also implies that R_t remains unbiased even if the average load per infection is calibrated to case data with time-varying underreporting. In contrast, if R_t was jointly inferred from both case and wastewater data, time-varying biases in case data could still impact transmission dynamic estimates, highlighting an important advantage of our calibration-based approach.

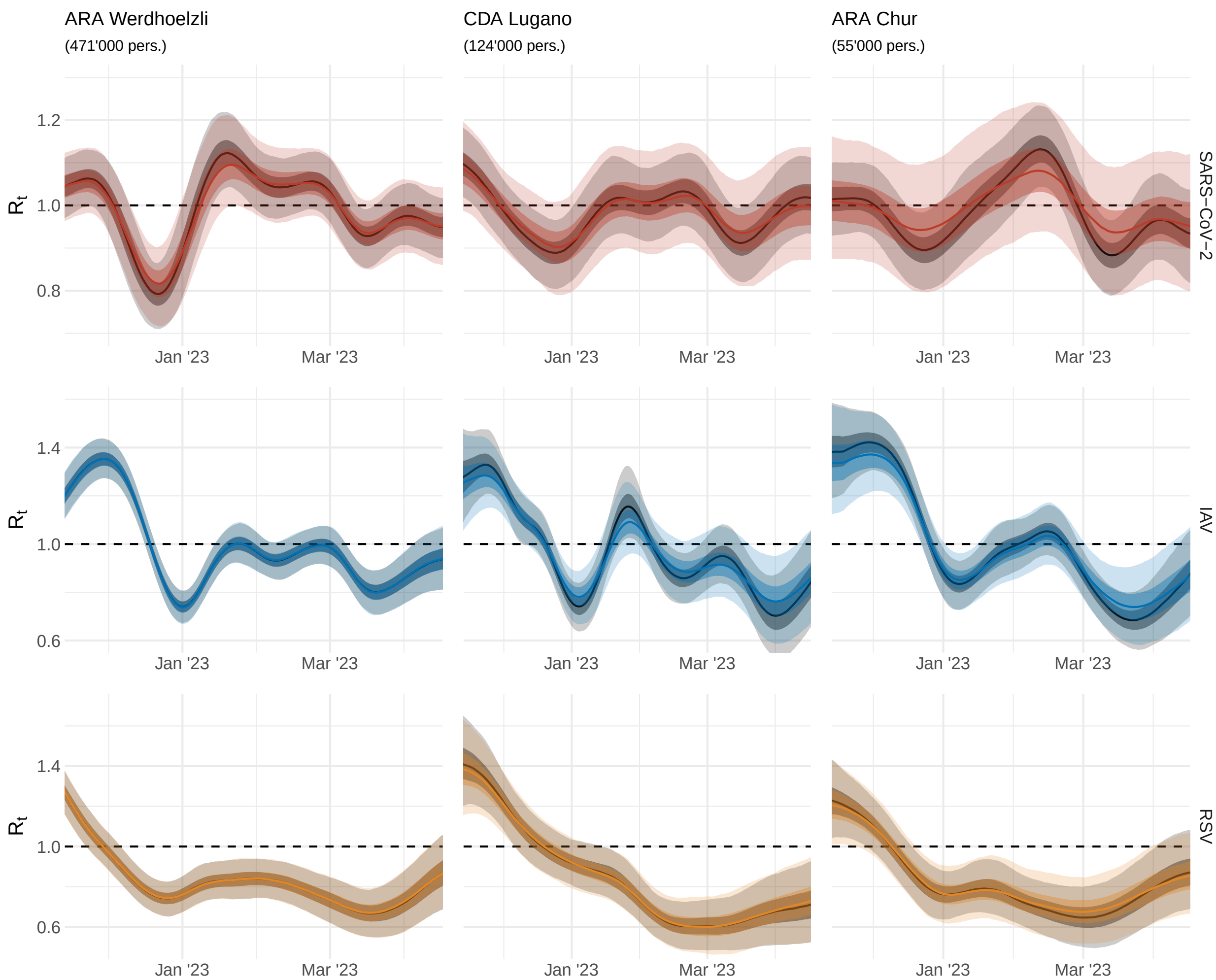

Supplementary Fig. 15. Effective reproduction number (R_t) estimates based on an alternative calibration of the shedding load per infection during the winter season 2022/23. Estimated R_t of SARS-CoV-2, influenza A virus (IAV), and respiratory syncytial virus (RSV) for the wastewater catchments of Zurich, Lugano, and Chur, Switzerland, during the winter season 2022/23 obtained when calibrating the total detectable shedding load per infection at the start of the season to 0.01% of the catchment population. For comparison, the corresponding R_t estimates from the main analysis are shown in grey. Lines indicate the posterior median and dark and light bands the 50% and 95% credible intervals of the marginal posterior distribution, respectively. Source data are provided as a Source Data file.

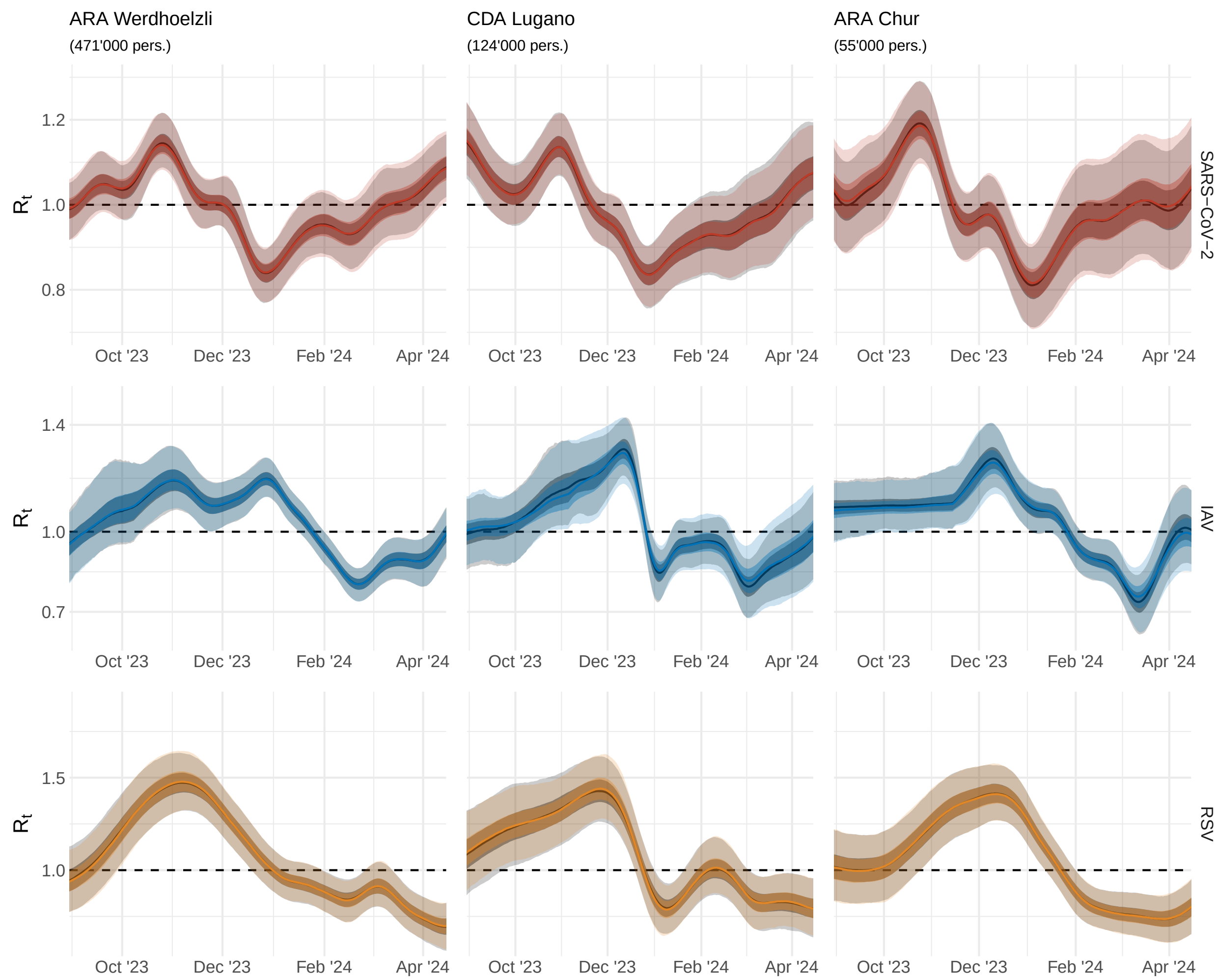

Supplementary Fig. 16. Effective reproduction number (R_t) estimates based on an alternative calibration of the shedding load per infection during the winter season 2023/24. Estimated R_t of SARS-CoV-2, influenza A virus (IAV), and respiratory syncytial virus (RSV) for the wastewater catchments of Zurich, Lugano, and Chur, Switzerland, during the winter season 2023/24 obtained when calibrating the total detectable shedding load per infection at the start of the season to 0.01% of the catchment population. For comparison, the corresponding R_t estimates from the main analysis are shown in grey. Lines indicate the posterior median and dark and light bands the 50% and 95% credible intervals of the marginal posterior distribution, respectively. Source data are provided as a Source Data file.

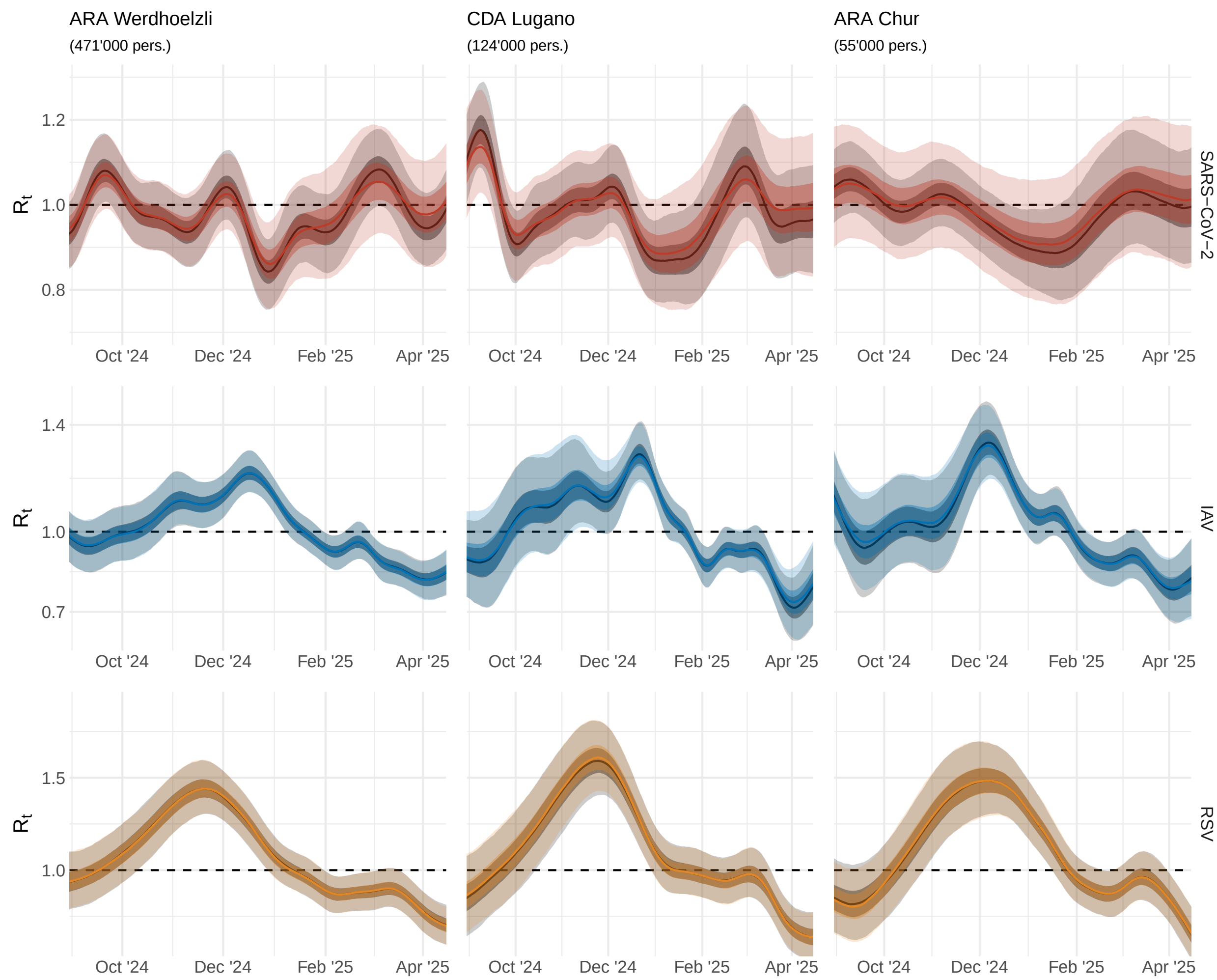

Supplementary Fig. 17. Effective reproduction number (R_t) estimates based on an alternative calibration of the shedding load per infection during the winter season 2024/25. Estimated R_t of SARS-CoV-2, influenza A virus (IAV), and respiratory syncytial virus (RSV) for the wastewater catchments of Zurich, Lugano, and Chur, Switzerland, during the winter season 2024/25 obtained when calibrating the total detectable shedding load per infection at the start of the season to 0.01% of the catchment population. For comparison, the corresponding R_t estimates from the main analysis are shown in grey. Lines indicate the posterior median and dark and light bands the 50% and 95% credible intervals of the marginal posterior distribution, respectively. Source data are provided as a Source Data file.

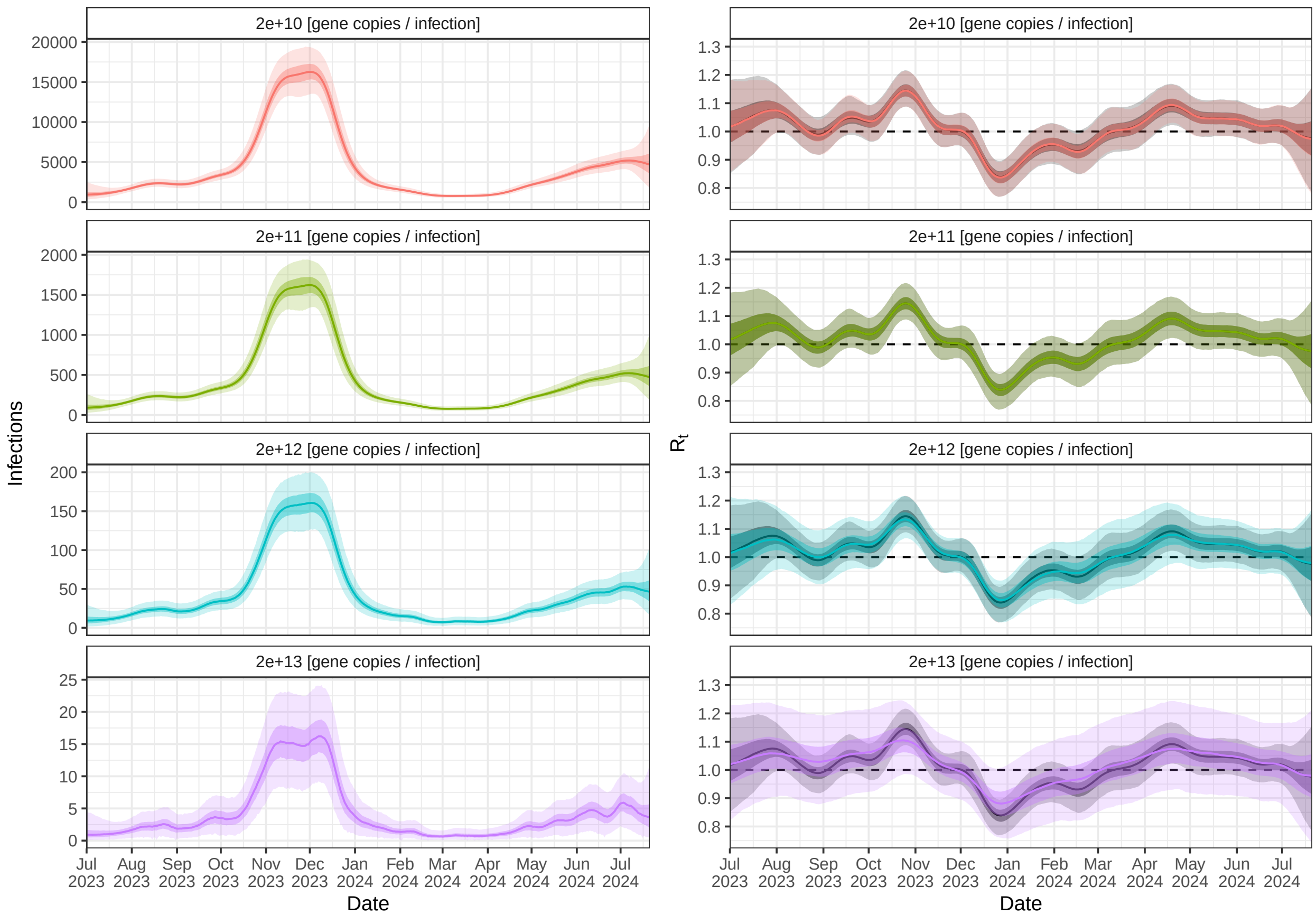

Supplementary Fig. 18. Sensitivity of estimated transmission dynamics to the assumed shedding load per infection. Shown are estimated infection numbers and effective reproduction number (R_t) of SARS-CoV-2 in Zurich during the winter season 2023/24, under different assumptions about the total detectable load shed per infection. Higher loads per infection correspond to lower infection numbers, and vice versa. In the main analysis, a load per infection of 2×10^{11} gene copies per infection was assumed. For comparison, the corresponding R_t estimate from the main analysis is shown in grey in all panels. Lines indicate the posterior median and dark and light bands the 50% and 95% credible intervals of the marginal posterior distribution, respectively. Source data are provided as a Source Data file.

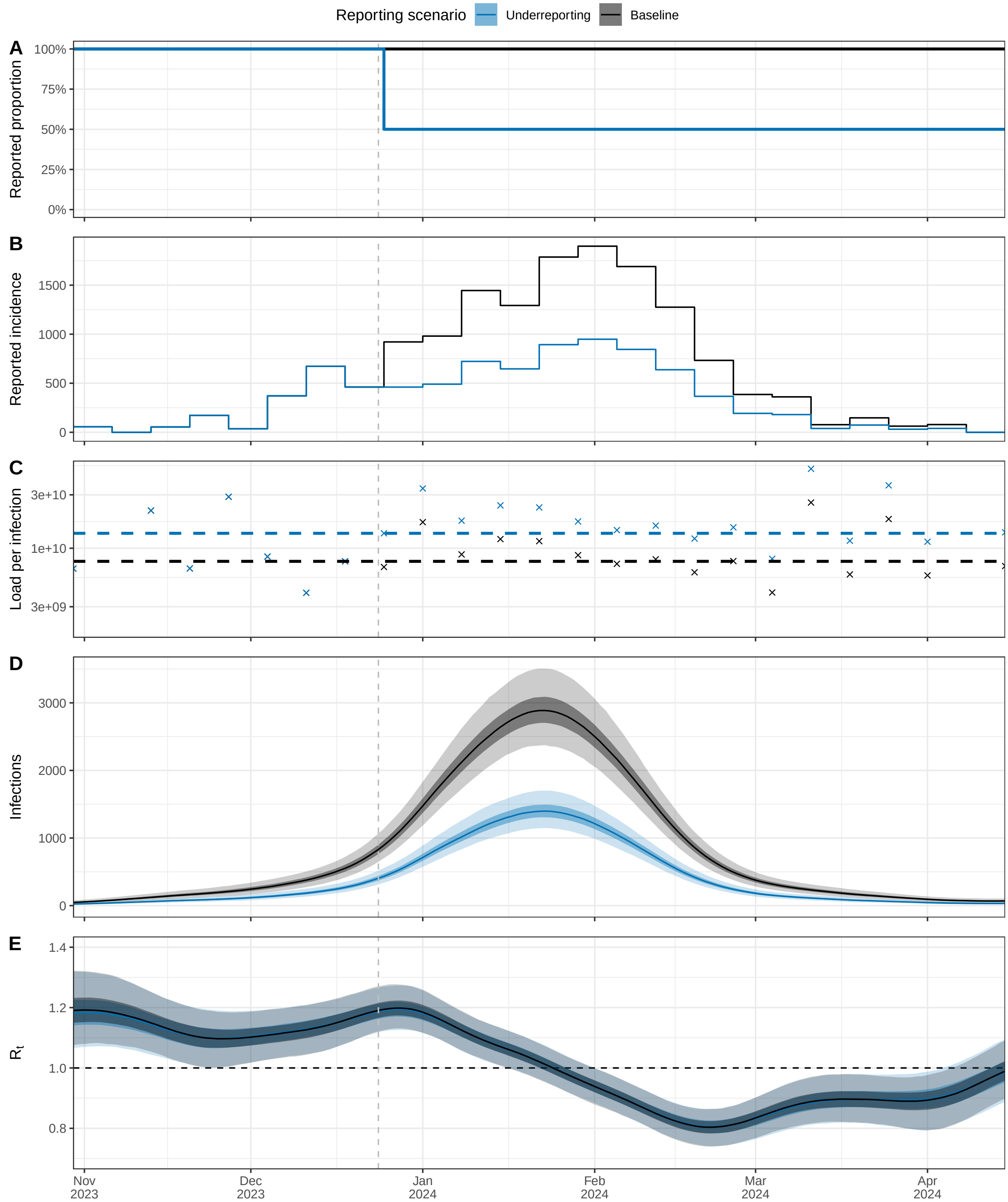

Supplementary Fig. 19. Robustness of estimated transmission dynamics to calibration from case data with time-varying underreporting. (A) We simulated a 50% reduction in the reporting of influenza A virus (IAV) infections in Zurich from December 24, 2023 onward (vertical dashed line). (B) This results in a time-varying bias of the reported incidence that would substantially distort case-based effective reproduction number (R_t) estimates. (C) Underreported incidence impacts the calibration of the average total detectable load per infection in our model (horizontal dashed lines). (D) This miscalibration also biases the number of new infections as estimated from wastewater, but only by a constant scaling factor. (E) R_t estimates remain robust to the miscalibration. In panels (D) and (E), lines indicate the posterior median and dark and light bands the 50% and 95% credible intervals of the marginal posterior distribution, respectively. Source data are provided as a Source Data file.

E.3.2 Individual-level variation in shedding loads

Supplementary Fig. 20 furthermore shows a sensitivity analysis of the assumed individual-level variation in shedding loads for the example of RSV in Zurich during the winter season 2023/24. Here we find that R_t estimates are practically identical for assumed coefficients of load variation of 0% (no variation), 100% (as in main text) or 200%. This result does not imply that individual-level variation in shedding has no effect on the noise of pathogen concentrations in wastewater. Rather, any unexplained variation in concentrations is captured by our model of measurement noise, leading to identical estimates of R_t. This also underlines the lack of identifiability of individual-level shedding variation from a single time series of concentration measurements with many other potential noise factors.

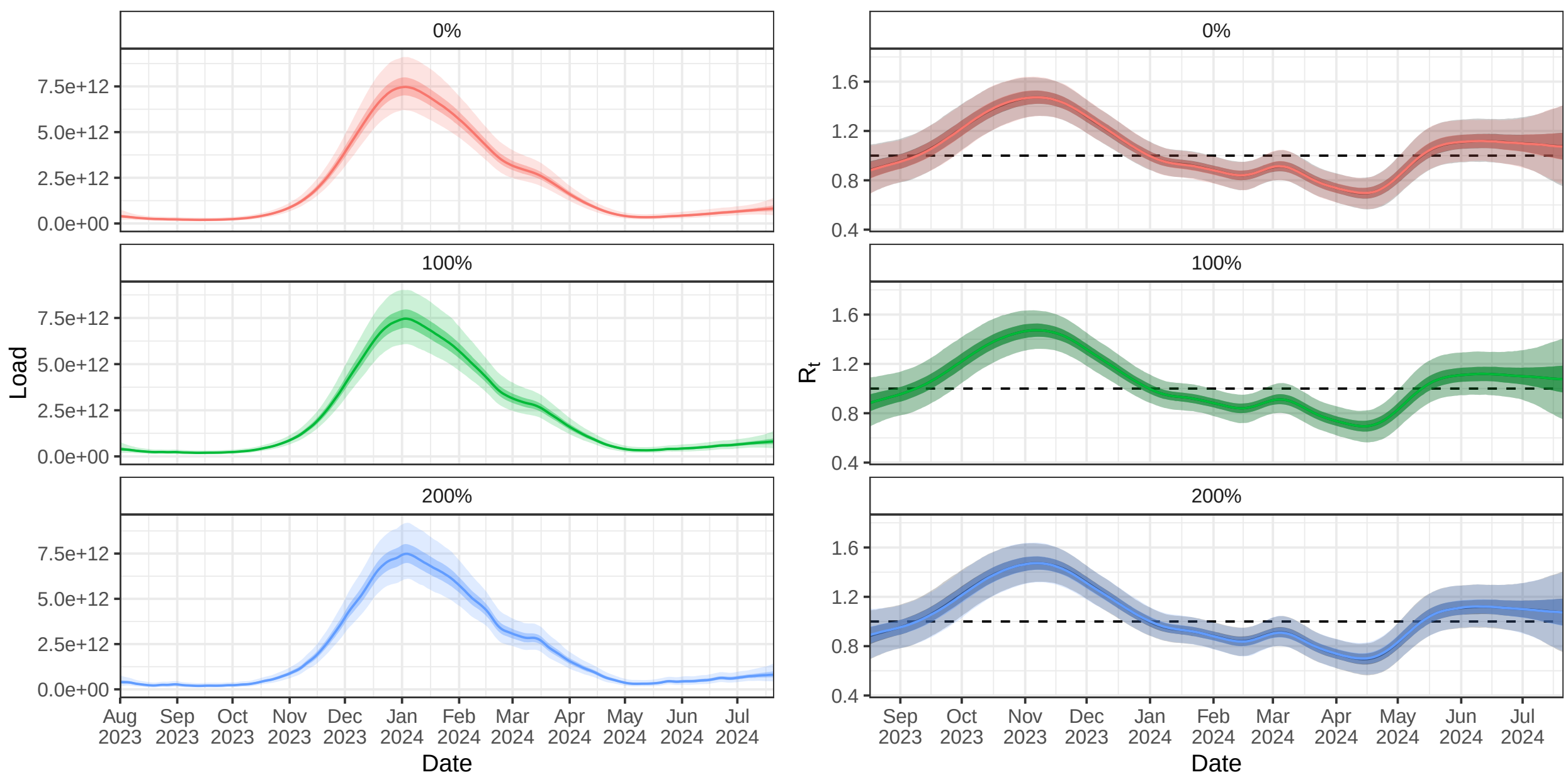

Supplementary Fig. 20. Sensitivity of estimated transmission dynamics to the assumed individual-level variation in shedding loads. Shown are estimated total daily loads in wastewater and effective reproduction number (R_t) of respiratory syncytial virus (RSV) in Zurich during the winter season 2023/24, under different assumptions about variation in total load shed by different individuals. In the main analysis, a coefficient of load variation of 100 % was assumed. For comparison, the corresponding R_t estimate from the main analysis is shown in grey in all panels. Lines indicate the posterior median and dark and light bands the 50% and 95% credible intervals of the marginal posterior distribution, respectively. Source data are provided as a Source Data file.

E.4 Subsampling

Supplementary Figs. 21 and 22 show the accuracy and uncertainty of estimated R_t and the variation in trajectories when subsampling our data to different combinations of weekdays. We estimated

R_t for all possible weekday combinations of each frequency (3 days per week, 1 day per week, and 1 day per 2 weeks). For a frequency of 3 days per week, R_t estimates deviated only slightly from the baseline (5 days per week) and had comparable uncertainty, with practically no dependence on the specific weekdays sampled. At lower frequencies, median R_t estimates deviated more from the baseline and credible intervals became increasingly wide. We also observed a dependence on the sampled weekdays, especially at the start of the IAV and RSV seasons. During this time period, a high proportion of non-detects means that, depending on the subsampled weekday, no positive measurement may be observed for several weeks. R_t estimates during this time period will then reflect our prior assumption of constant transmission.

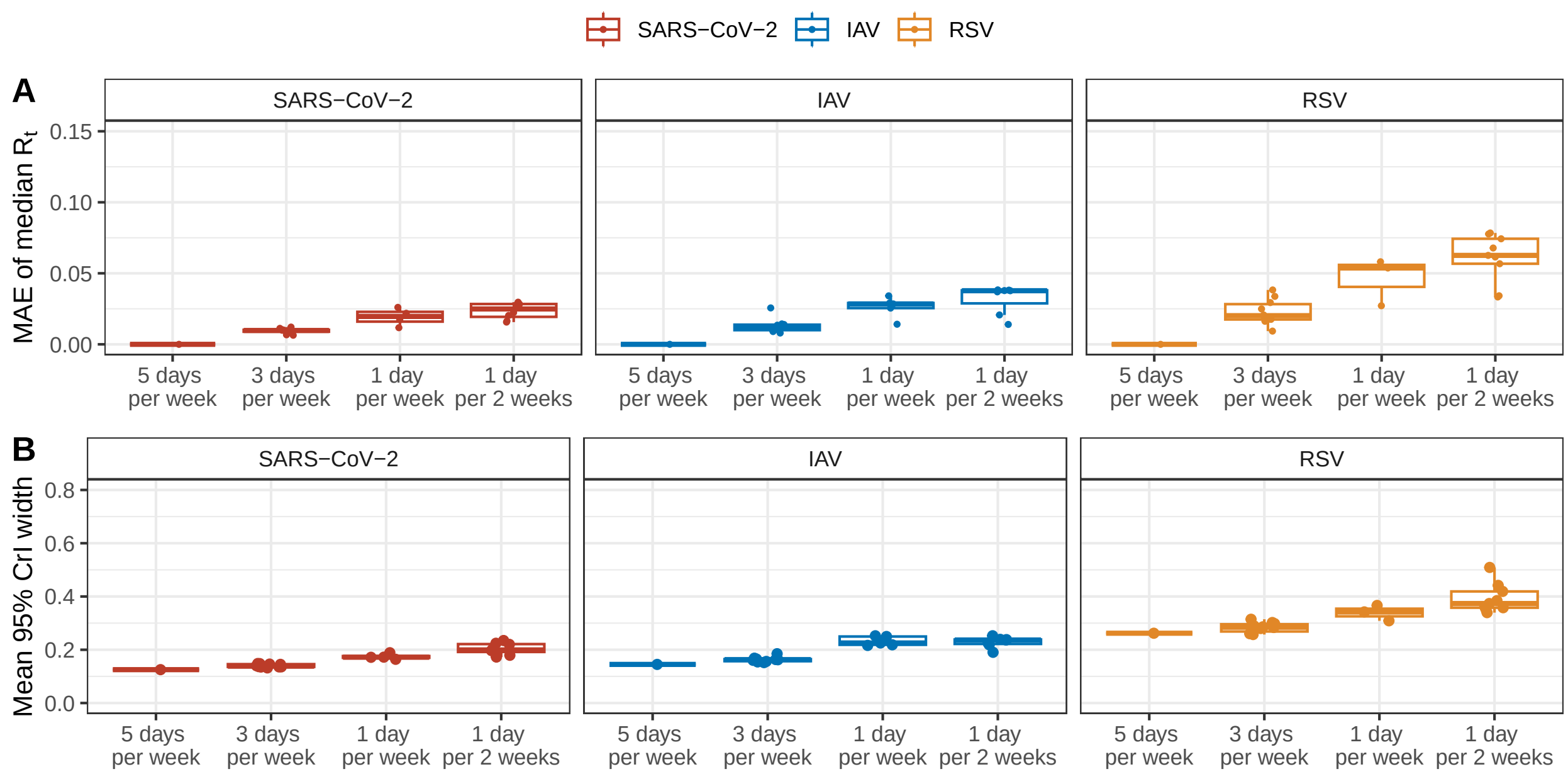

Supplementary Fig. 21. Accuracy and uncertainty of effective reproduction number (R_t) estimates under sparse measurement. (A) Mean absolute error (MAE) of median R_t estimates from subsampled measurements compared to the baseline using all measurements (5 days per week), for all possible weekday combinations, respectively. (B) Mean width of the 95% credible intervals for R_t estimates from subsampled measurements, for all possible weekday combinations, respectively. Box plots show the median (centre line), 25th and 75th percentiles (box bounds), and 1.5x the interquartile range (whiskers), across all possible weekday combinations, respectively. Estimates used data for the catchment of Zurich until May 31, 2024, subsampled to a frequency of 3 days per week, 1 day per week, and 1 day per 2 weeks. Source data are provided as a Source Data file.

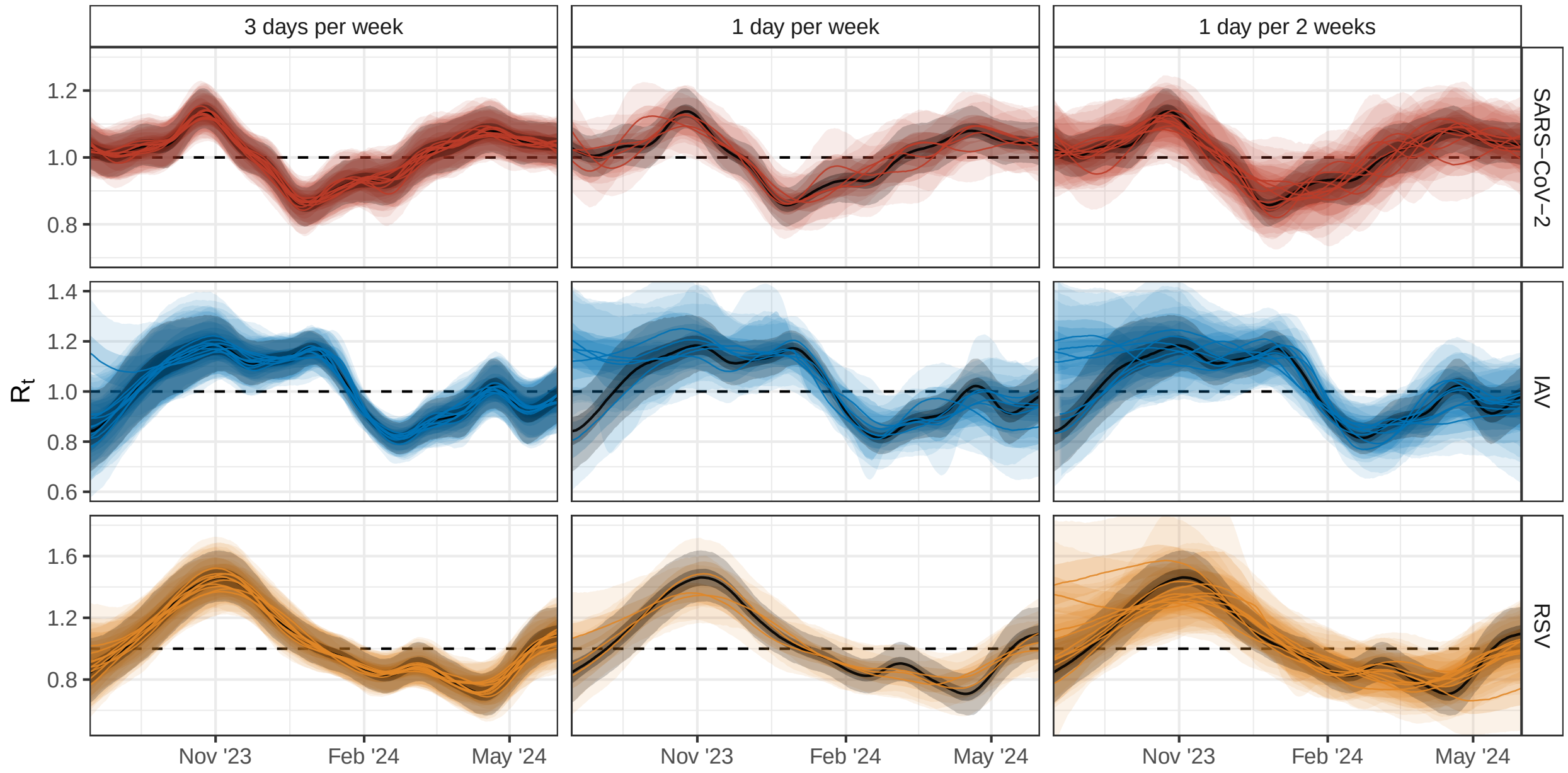

Supplementary Fig. 22. Sensitivity of effective reproduction number (R_t) estimates to sampled weekdays under sparse measurement. Retrospective R_t estimates on May 31, 2024 at the catchment of Zurich, based on samples on 3 days per week, 1 day per week, and 1 day per 2 weeks, overlayed for all possible weekday combinations, respectively. The baseline using all available measurements (5 days per week) is shown in grey. Lines indicate the posterior median and dark and light bands the 50% and 95% credible intervals of the marginal posterior distribution, respectively. Source data are provided as a Source Data file.

E.5 Catchment population size

To test the potential effect of commuting patterns that influence the size of the catchment population on transmission dynamic estimates, we simulated a reduction of the total load shed on weekends and holidays for the example of SARS-CoV-2 in Zurich during the 2023/24 winter season. Based on commuting statistics for the city of Zurich [35], we estimated a peak net increase of 156 000 persons ($\approx 35\%$ of the residential population) during working days. Assuming independence between infection and commuting behaviour, and shedding at the place of work, this implies $\approx 35\%$ higher loads during working days compared to the weekend, or respectively, $\approx 25\%$ lower loads during the weekend compared to the working week. Thus, we simulated a 25% reduction in the pathogen load shed during weekends and public holidays for the catchment of Zurich by scaling down the measured concentrations accordingly. When fitting our model to these modified measurements, we obtained highly similar R_t estimates (Supplementary Fig. 23), suggesting that regular weekly patterns in the shedding population have a limited effect on transmission dynamic estimates.

We note that our simulation did not account for higher measurement noise due to reduced

concentrations on weekends, which could in principle increase the uncertainty of estimates [36]. Moreover, we did not account for population structure and the effects of commuting on the transmission process. In addition, further mobility patterns such as from shopping, leisure activities, and tourism that could impact the time-varying catchment population have not been included. At the same time, we remark that the effective load shed in the catchment could also be more stable than the population size, since shedding may be predominantly residential (e. g. as individuals may shed primarily outside of working hours or stay at home when symptomatic).

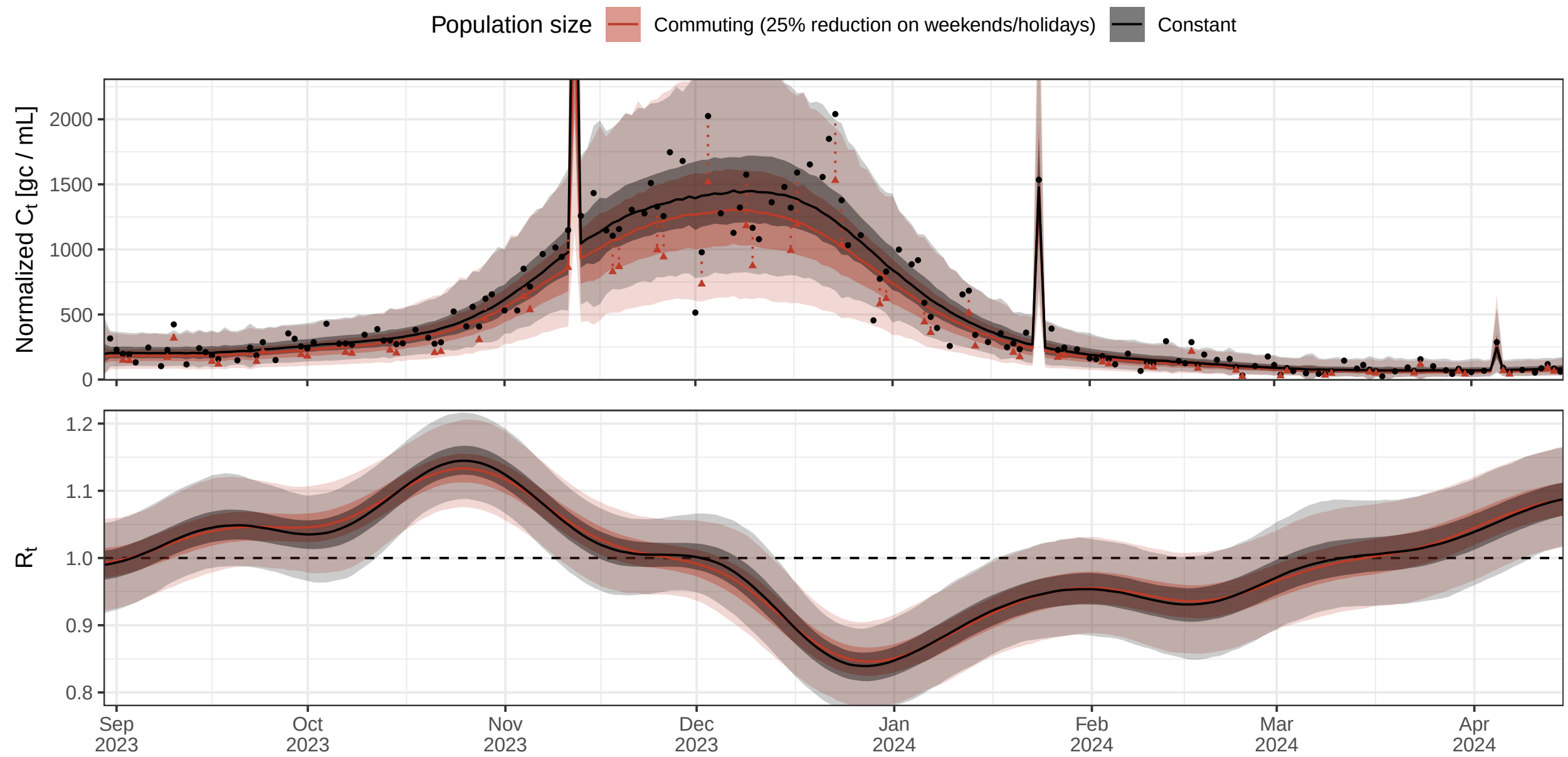

Supplementary Fig. 23. Sensitivity of effective reproduction number (R_t) estimates to simulated weekly seasonality in catchment population size. We modified observed concentration measurements of SARS-CoV-2 for the catchment of Zurich during the 2023/24 winter season (black) to simulate a commuting-induced weekly seasonality in the load shed in the catchment (red). Shown are flow-adjusted (i. e. rescaled to the median flow) observed and predicted concentration measurements (top) and corresponding R_t estimates (bottom). Lines indicate the posterior median and dark and light bands the 50% and 95% credible intervals of the marginal posterior distribution for concentrations and R_t, respectively. Source data are provided as a Source Data file.

F Additional results

In the main text, we showed the estimated reproduction number R_t for the catchments of Zurich, Lugano, and Chur during the winter season 2023/24. R_t estimates for these catchments during the winter seasons 2022/23 and 2024/25 are shown in Supplementary Figs. 24 and 25. The real-time performance of R_t estimates and short-term concentration forecasts for these seasons is assessed in Supplementary Figs. 26 and 27. Moreover, Supplementary Figs. 28 to 30 show the real-time mean absolute error (MAE) of median R_t estimates for the winter seasons 2022/23, 2023/24 and 2024/25, respectively. Estimates of the epidemic growth rate r_t for all three winter seasons are shown in Supplementary Figs. 31 to 33. Real-time results are quantitatively summarized in Supplementary Tables 4 and 5. Finally, R_t estimates for all other sampled catchments during the seasons 2022/23, 2023/24, and 2024/25 are shown in Supplementary Figs. 34 to 41.

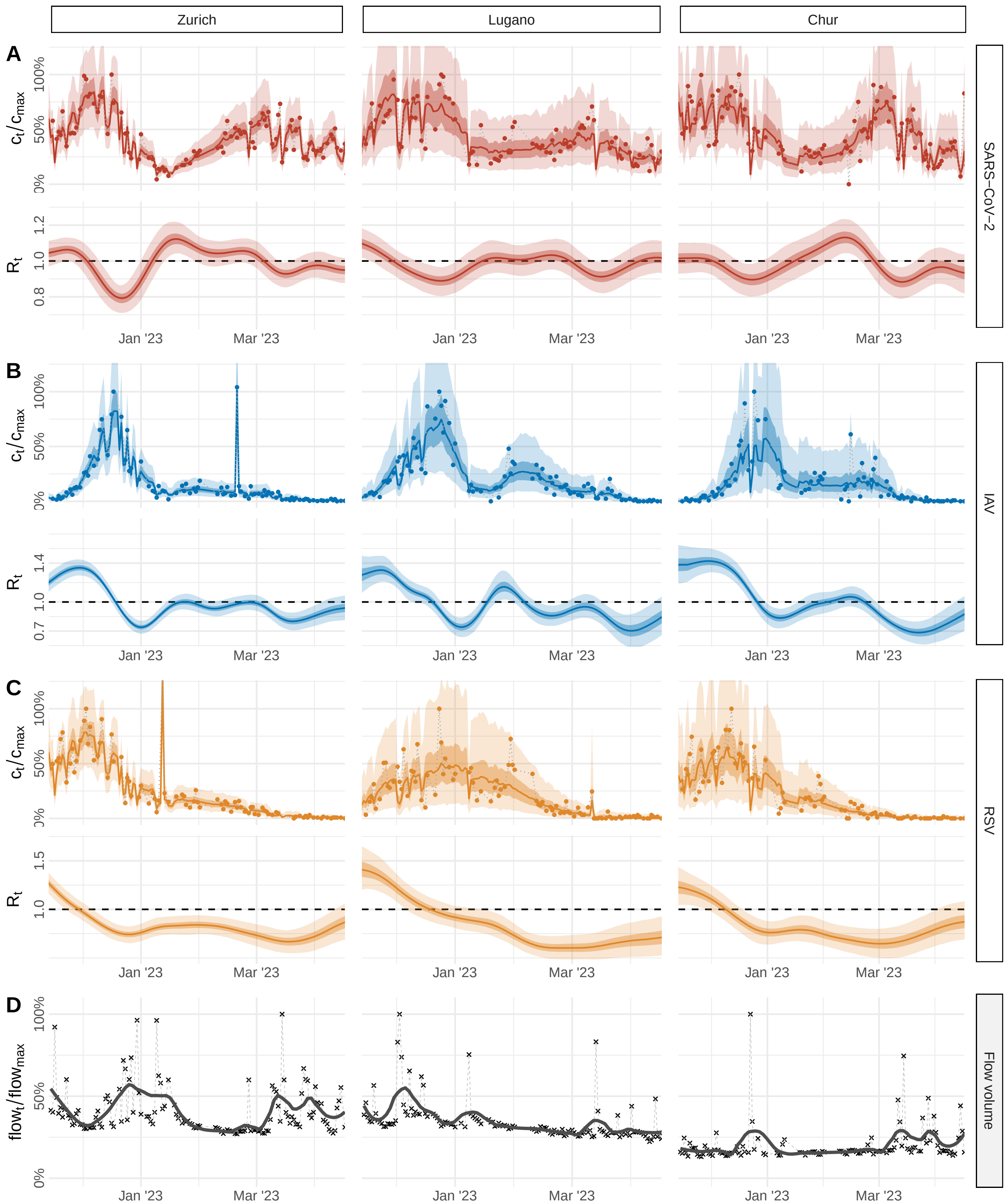

Supplementary Fig. 24. Viral transmission dynamics in Switzerland quantified from wastewater at treatment plants in Zurich, Chur, and Lugano, 2022/23 winter season. (A-C) Relative pathogen concentrations (top) and estimates of the effective reproduction number R_t (bottom) for SARS-CoV-2, influenza A virus (IAV), and respiratory syncytial virus (RSV) based on longitudinal samples from a large (Zurich, 471 000 persons), medium-size (Lugano, 124 000 persons), and small (Chur, 55 000 persons) wastewater catchment in Switzerland during the winter season 2022/23. Shown are measured pathogen concentrations (dots), and the posterior median (lines) and 50% and 95% credible intervals (dark and light bands) of predicted concentrations and estimated R_t, respectively. (D) Relative daily flow at the sampled wastewater treatment plant of each catchment. Lines show a smooth trend estimated using LOESS with a 4-week window. Source data are provided as a Source Data file.

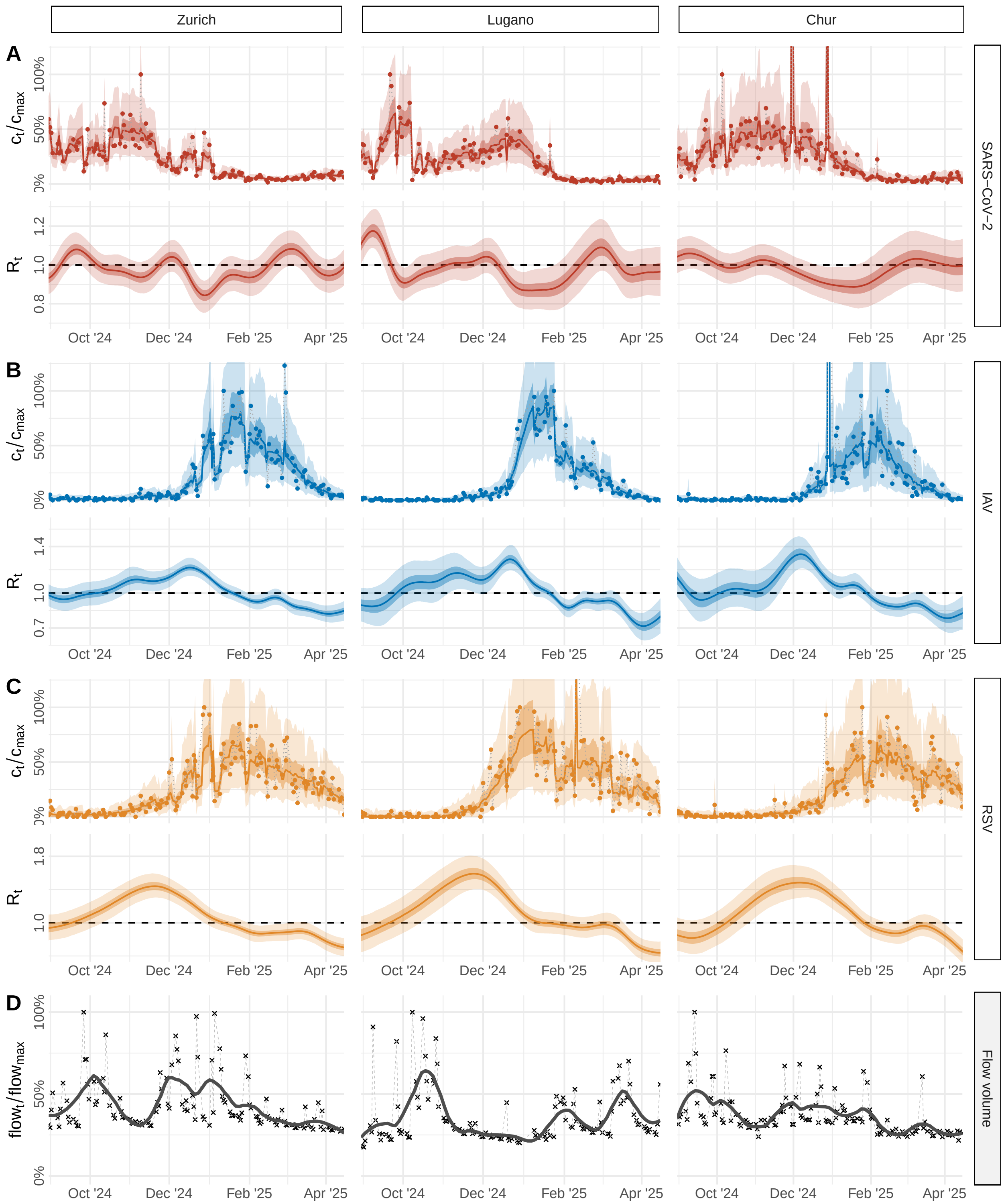

Supplementary Fig. 25. Viral transmission dynamics in Switzerland quantified from wastewater at treatment plants in Zurich, Chur, and Lugano, 2024/25 winter season. (A-C) Relative pathogen concentrations (top) and estimates of the effective reproduction number R_t (bottom) for SARS-CoV-2, influenza A virus (IAV), and respiratory syncytial virus (RSV) based on longitudinal samples from a large (Zurich, 471 000 persons), medium-size (Lugano, 124 000 persons), and small (Chur, 55 000 persons) wastewater catchment in Switzerland during the winter season 2024/25. Shown are measured pathogen concentrations (dots), and the posterior median (lines) and 50% and 95% credible intervals (dark and light bands) of predicted concentrations and estimated R_t, respectively. (D) Relative daily flow at the sampled wastewater treatment plant of each catchment. Lines show a smooth trend estimated using LOESS with a 4-week window. Source data are provided as a Source Data file.

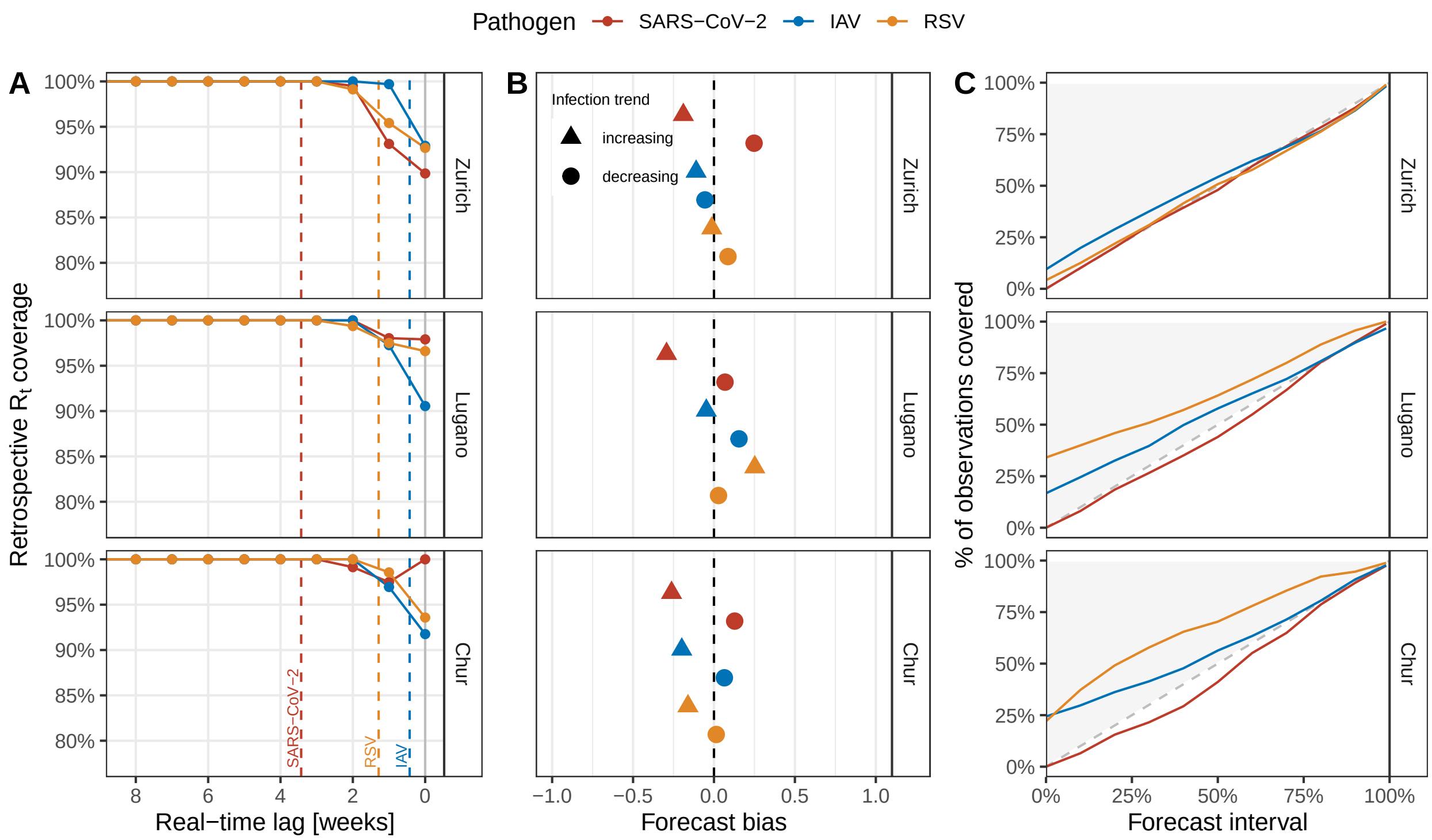

Supplementary Fig. 26. Real-time performance of wastewater-based reproduction number estimates and short-term concentration forecasts during the winter season 2022/23. Shown is the performance of real-time effective reproduction number (R_t) estimates and 1–14 day ahead forecasts of concentration measurements for the wastewater catchments of Zurich, Lugano, and Chur, Switzerland, during the 2022/23 seasonal wave of SARS-CoV-2 (red), IAV (blue), and RSV (orange). (A) Consistency of real-time R_t (estimated on each day with new data) with retrospective R_t (estimated at the end of the seasonal wave). For each real-time R_t estimate, we computed the retrospective coverage, i. e. the percentage of days on which the 95% credible interval (CrI) contained the retrospective median R_t. Dots show the coverage stratified by lags of 0–8 weeks from the date of estimation. Vertical dashed lines show the 90% quantile of the temporal shedding profile of each pathogen. (B) Bias of concentration measurement forecasts, stratified by increasing and decreasing infection trends. Scores range between -1 (maximal underprediction) and 1 (maximal overprediction); 0 indicates no bias. (C) Calibration of concentration measurement forecasts. The diagonal line indicates a perfectly calibrated forecast, where all intervals cover exactly their nominal share of observations. Source data are provided as a Source Data file.

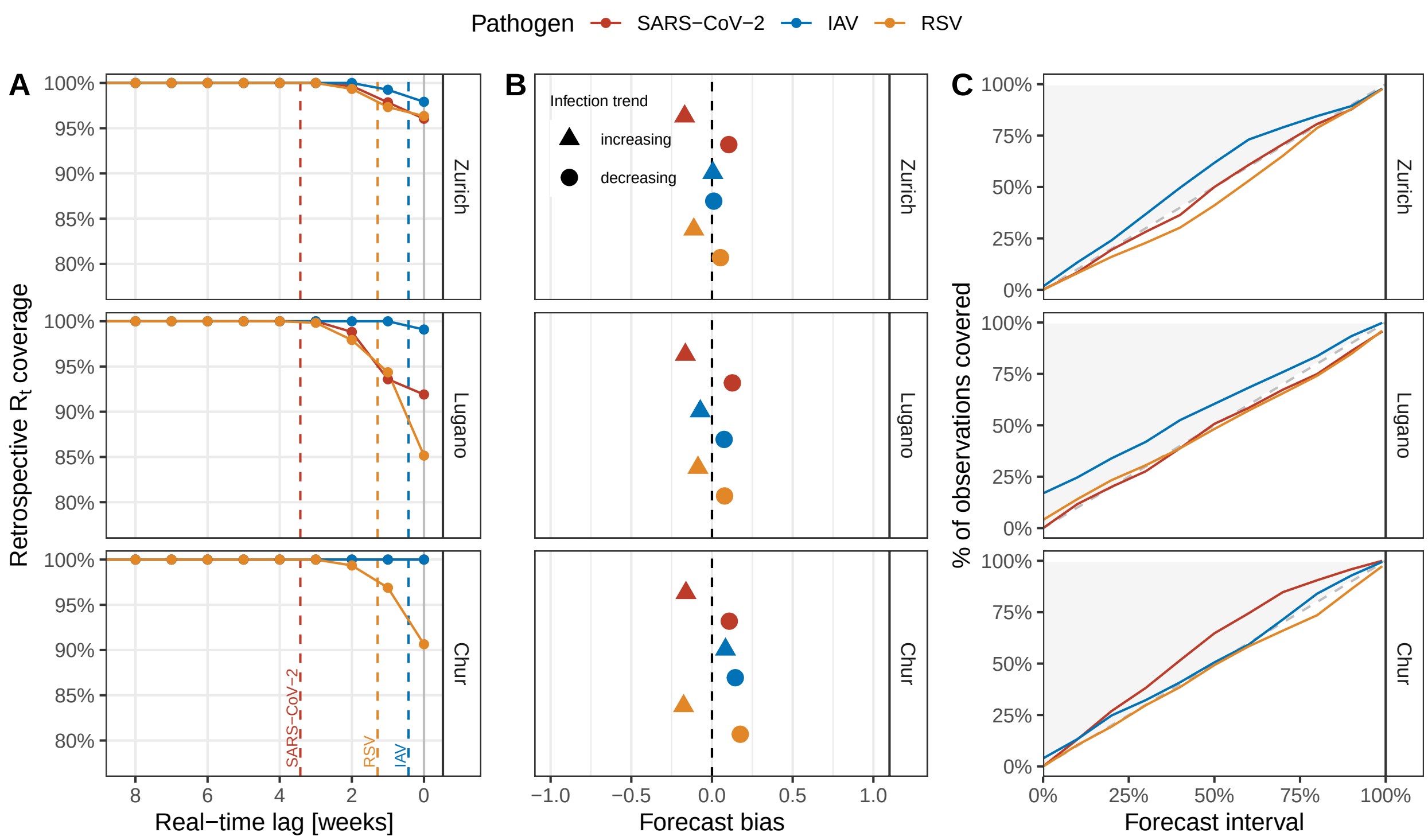

Supplementary Fig. 27. Real-time performance of wastewater-based reproduction number estimates and short-term concentration forecasts during the winter season 2024/25. Shown is the performance of real-time effective reproduction number (R_t) estimates and 1–14 day ahead forecasts of concentration measurements for the wastewater catchments of Zurich, Lugano, and Chur, Switzerland, during the 2024/25 seasonal wave of SARS-CoV-2 (red), IAV (blue), and RSV (orange). (A) Consistency of real-time R_t (estimated on each day with new data) with retrospective R_t (estimated at the end of the seasonal wave). For each real-time R_t estimate, we computed the retrospective coverage, i. e. the percentage of days on which the 95% credible interval (CrI) contained the retrospective median R_t. Dots show the coverage stratified by lags of 0–8 weeks from the date of estimation. Vertical dashed lines show the 90% quantile of the temporal shedding profile of each pathogen. Note that for Chur, coverage was 100% at all lags for both SARS-CoV-2 and IAV. (B) Bias of concentration measurement forecasts, stratified by increasing and decreasing infection trends. Scores range between -1 (maximal underprediction) and 1 (maximal overprediction); 0 indicates no bias. (C) Calibration of concentration measurement forecasts. The diagonal line indicates a perfectly calibrated forecast, where all intervals cover exactly their nominal share of observations. Source data are provided as a Source Data file.

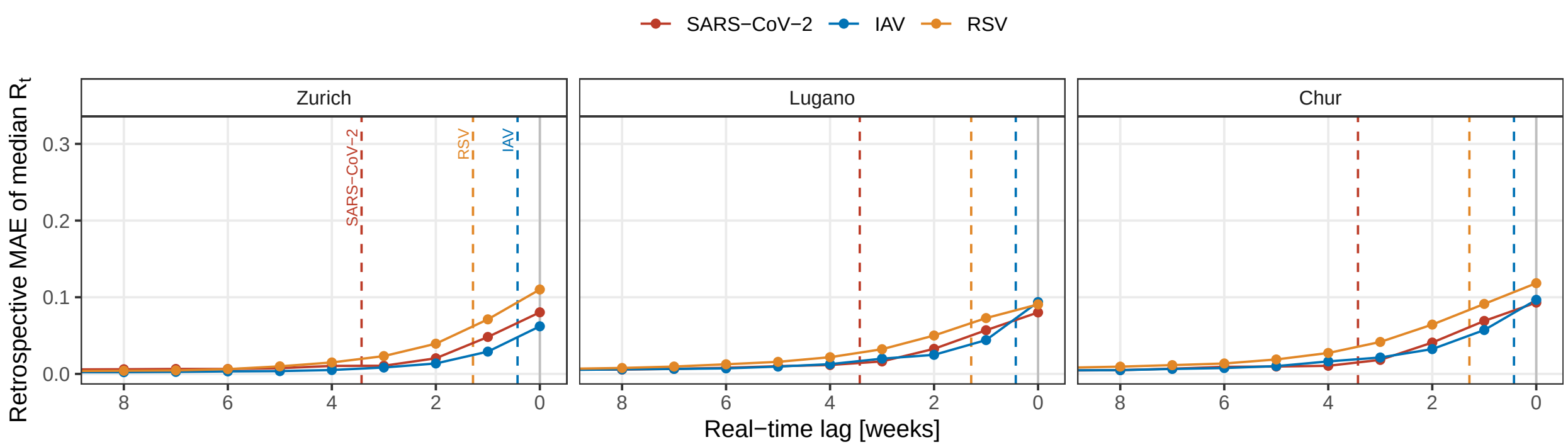

Supplementary Fig. 28. Real-time accuracy of wastewater-based effective reproduction number (R_t) estimates during the winter season 2022/23. Dots show the mean absolute error (MAE) of the real-time median R_t (estimated on each day with new data) compared to the retrospective median R_t (estimated at the end of the seasonal wave) for the wastewater catchments of Zurich, Lugano, and Chur, Switzerland, during the 2022/23 seasonal wave of SARS-CoV-2 (red), IAV (blue), and RSV (orange). Source data are provided as a Source Data file.

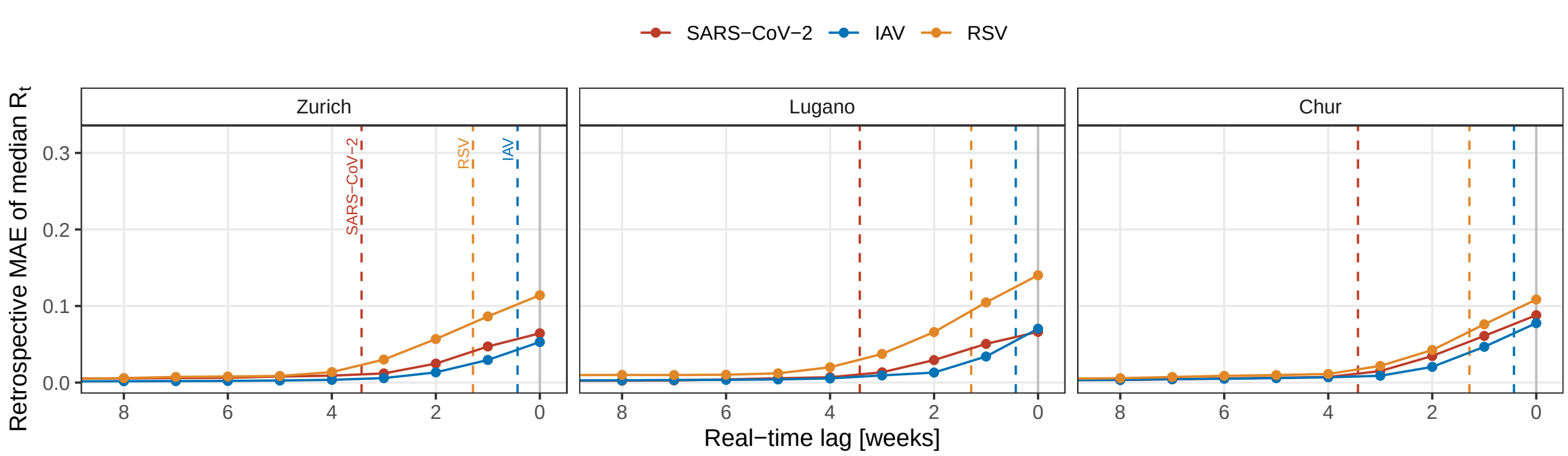

Supplementary Fig. 29. Real-time accuracy of wastewater-based effective reproduction number (R_t) estimates during the winter season 2023/24. Dots show the mean absolute error (MAE) of the real-time median R_t (estimated on each day with new data) compared to the retrospective median R_t (estimated at the end of the seasonal wave) for the wastewater catchments of Zurich, Lugano, and Chur, Switzerland, during the 2023/24 seasonal wave of SARS-CoV-2 (red), IAV (blue), and RSV (orange). Source data are provided as a Source Data file.

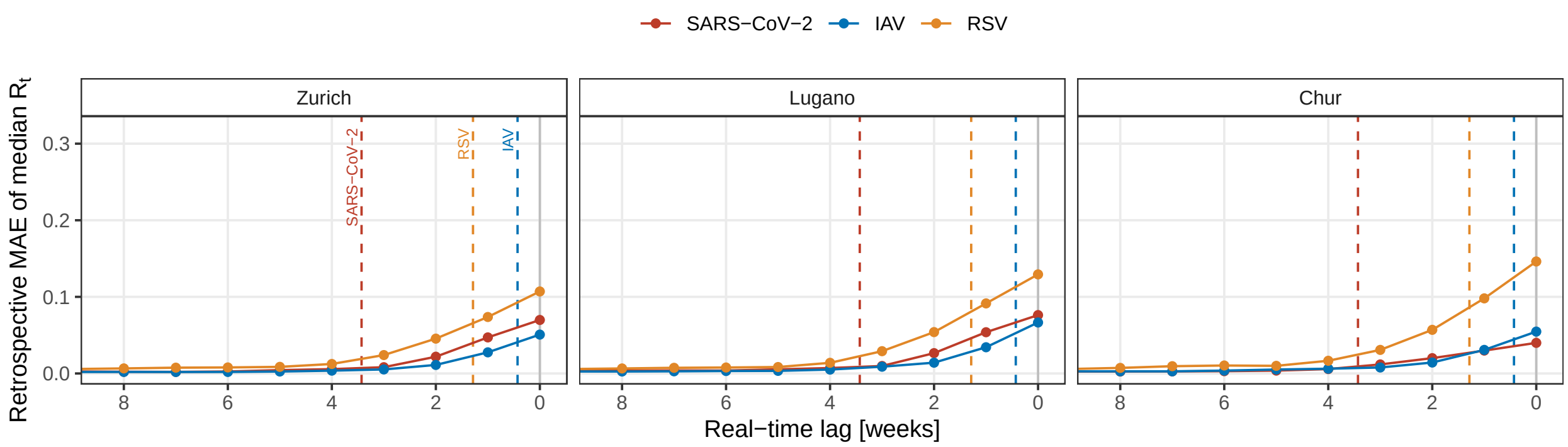

Supplementary Fig. 30. Real-time accuracy of wastewater-based effective reproduction number (R_t) estimates during the winter season 2024/25. Dots show the mean absolute error (MAE) of the real-time median R_t (estimated on each day with new data) compared to the retrospective median R_t (estimated at the end of the seasonal wave) for the wastewater catchments of Zurich, Lugano, and Chur, Switzerland, during the 2024/25 seasonal wave of SARS-CoV-2 (red), IAV (blue), and RSV (orange). Source data are provided as a Source Data file.

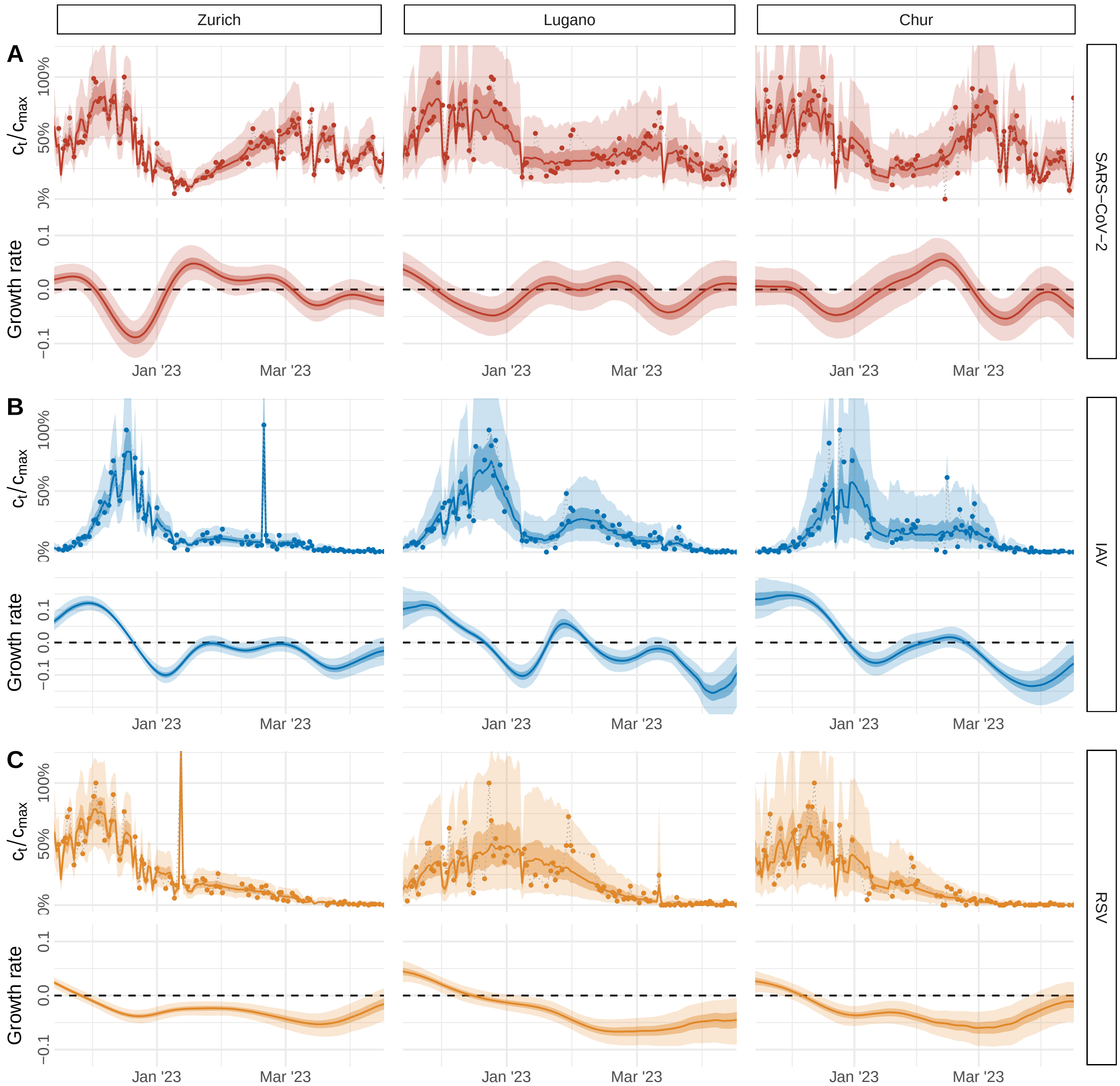

Supplementary Fig. 31. Estimated epidemic growth rates during the winter season 2022/23. (A-C) Relative pathogen concentrations (top) and estimates of the epidemic growth rate r_t (bottom) for SARS-CoV-2, influenza A virus (IAV), and respiratory syncytial virus (RSV) based on longitudinal samples from a large (Zurich, 471 000 persons), medium-size (Lugano, 124 000 persons), and small (Chur, 55 000 persons) wastewater catchment in Switzerland during the winter season 2022/23. Shown are measured pathogen concentrations (dots), and the posterior median (lines) and 50% and 95% credible intervals (dark and light bands) of predicted concentrations and estimated r_t, respectively. Source data are provided as a Source Data file.

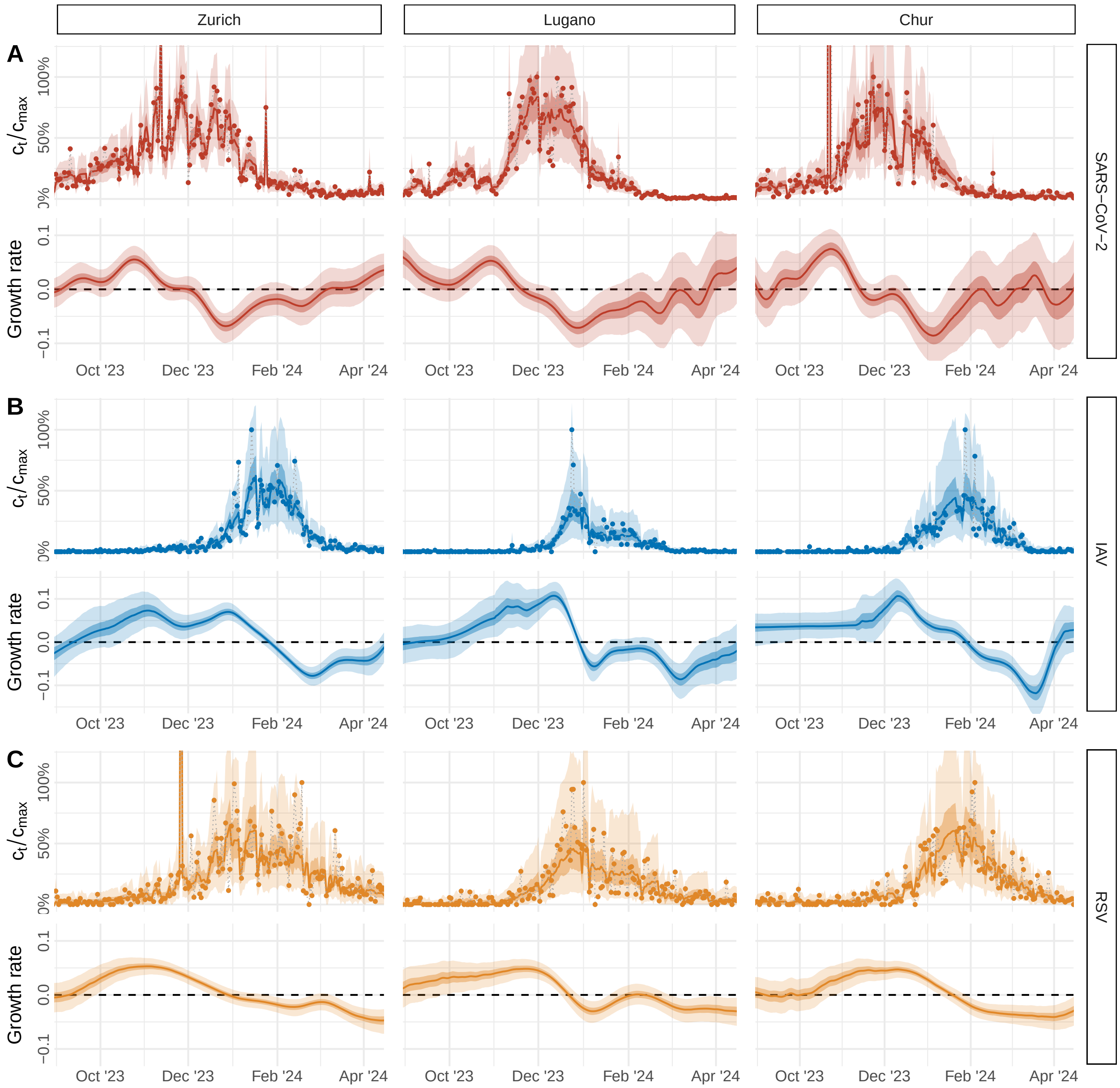

Supplementary Fig. 32. Estimated epidemic growth rates during the winter season 2023/24. (A-C) Relative pathogen concentrations (top) and estimates of the epidemic growth rate r_t (bottom) for SARS-CoV-2, influenza A virus (IAV), and respiratory syncytial virus (RSV) based on longitudinal samples from a large (Zurich, 471 000 persons), medium-size (Lugano, 124 000 persons), and small (Chur, 55 000 persons) wastewater catchment in Switzerland during the winter season 2023/24. Shown are measured pathogen concentrations (dots), and the posterior median (lines) and 50% and 95% credible intervals (dark and light bands) of predicted concentrations and estimated r_t, respectively. Source data are provided as a Source Data file.

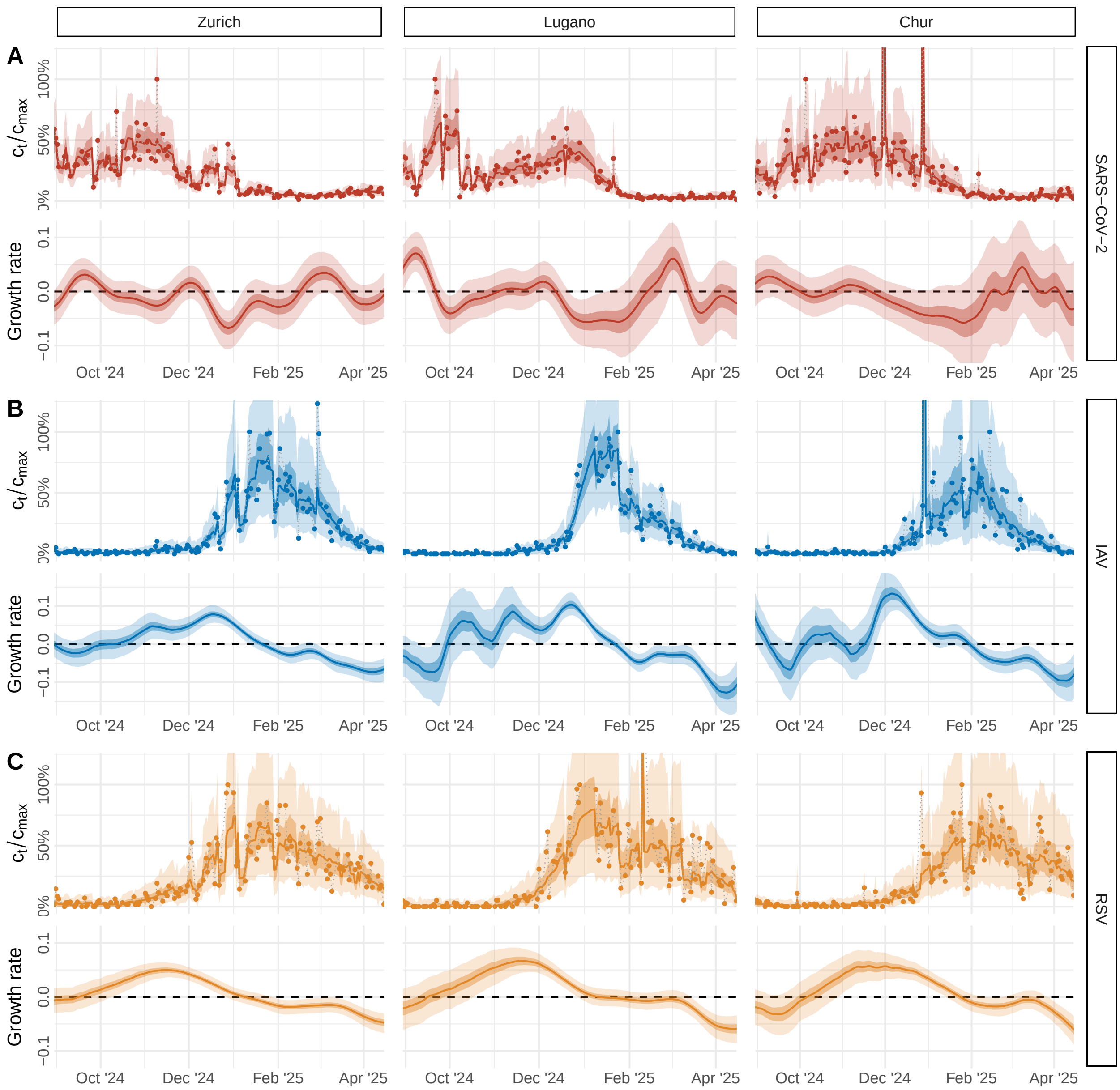

Supplementary Fig. 33. Estimated epidemic growth rates during the winter season 2024/25. (A-C) Relative pathogen concentrations (top) and estimates of the epidemic growth rate r_t (bottom) for SARS-CoV-2, influenza A virus (IAV), and respiratory syncytial virus (RSV) based on longitudinal samples from a large (Zurich, 471 000 persons), medium-size (Lugano, 124 000 persons), and small (Chur, 55 000 persons) wastewater catchment in Switzerland during the winter season 2024/25. Shown are measured pathogen concentrations (dots), and the posterior median (lines) and 50% and 95% credible intervals (dark and light bands) of predicted concentrations and estimated r_t, respectively. Source data are provided as a Source Data file.

Supplementary Table 4. Real-time consistency of R_t estimates across pathogens, seasons, and locations. Source data are provided as a Source Data file.

			Coverage (0-4 weeks lag)					MAE (0-4 weeks lag)				
Pathogen	Season	Location	0	1	2	3	4	0	1	2	3	4
SARS-CoV-2	2022/23	Zurich	90%	93%	100%	100%	100%	0.07	0.05	0.02	0.01	0.01
		Lugano	98%	98%	100%	100%	100%	0.08	0.05	0.03	0.01	0.01
		Chur	100%	97%	99%	100%	100%	0.04	0.03	0.02	0.01	0.01
	2023/24	Zurich	97%	97%	99%	100%	100%	0.07	0.05	0.02	0.01	0.01
		Lugano	100%	100%	100%	100%	100%	0.08	0.05	0.03	0.01	0.01
		Chur	100%	99%	100%	100%	100%	0.04	0.03	0.02	0.01	0.01
	2024/25	Zurich	96%	98%	100%	100%	100%	0.07	0.05	0.02	0.01	0.01
		Lugano	92%	94%	99%	100%	100%	0.08	0.05	0.03	0.01	0.01
		Chur	100%	100%	100%	100%	100%	0.04	0.03	0.02	0.01	0.01
IAV	2022/23	Zurich	93%	100%	100%	100%	100%	0.05	0.03	0.01	0.01	0.00
		Lugano	91%	97%	100%	100%	100%	0.07	0.03	0.01	0.01	0.01
		Chur	92%	97%	100%	100%	100%	0.05	0.03	0.01	0.01	0.01
	2023/24	Zurich	100%	100%	100%	100%	100%	0.05	0.03	0.01	0.01	0.00
		Lugano	88%	98%	100%	100%	100%	0.07	0.03	0.01	0.01	0.01
		Chur	94%	99%	100%	100%	100%	0.05	0.03	0.01	0.01	0.01
	2024/25	Zurich	98%	99%	100%	100%	100%	0.05	0.03	0.01	0.01	0.00
		Lugano	99%	100%	100%	100%	100%	0.07	0.03	0.01	0.01	0.01
		Chur	100%	100%	100%	100%	100%	0.05	0.03	0.01	0.01	0.01
RSV	2022/23	Zurich	93%	95%	99%	100%	100%	0.11	0.07	0.05	0.02	0.01
		Lugano	97%	98%	99%	100%	100%	0.13	0.09	0.05	0.03	0.01
		Chur	94%	99%	100%	100%	100%	0.15	0.10	0.06	0.03	0.02
	2023/24	Zurich	96%	94%	97%	100%	100%	0.11	0.07	0.05	0.02	0.01
		Lugano	87%	89%	96%	100%	100%	0.13	0.09	0.05	0.03	0.01
		Chur	96%	99%	100%	100%	100%	0.15	0.10	0.06	0.03	0.02
	2024/25	Zurich	96%	97%	99%	100%	100%	0.11	0.07	0.05	0.02	0.01
		Lugano	85%	94%	98%	100%	100%	0.13	0.09	0.05	0.03	0.01
		Chur	91%	97%	99%	100%	100%	0.15	0.10	0.06	0.03	0.02

Supplementary Table 5. Bias of concentration forecasts across pathogens, seasons, and locations. Source data are provided as a Source Data file.

			Bias	
Pathogen	Season	Location	Infections decreasing	Infections increasing
SARS-CoV-2	2022/23	Zurich	24.8%	-18.9%
		Lugano	6.8%	-29.3%
		Chur	12.8%	-26.2%
	2023/24	Zurich	3.8%	-3.9%
		Lugano	9.0%	-2.7%
		Chur	16.5%	-21.7%
	2024/25	Zurich	10.4%	-16.9%
		Lugano	12.6%	-16.5%
		Chur	10.7%	-16.1%
IAV	2022/23	Zurich	-5.6%	-11.0%
		Lugano	15.5%	-4.7%
		Chur	6.4%	-19.9%
	2023/24	Zurich	7.8%	-4.2%
		Lugano	-0.2%	-12.6%
		Chur	11.3%	-6.1%
	2024/25	Zurich	1.1%	0.5%
		Lugano	7.5%	-7.3%
		Chur	14.5%	8.4%
RSV	2022/23	Zurich	8.7%	-1.4%
		Lugano	2.8%	25.2%
		Chur	1.4%	-16.1%
	2023/24	Zurich	13.4%	-4.3%
		Lugano	9.4%	-24.6%
		Chur	13.5%	-10.0%
	2024/25	Zurich	5.3%	-11.3%
		Lugano	7.8%	-8.7%
		Chur	17.5%	-17.6%

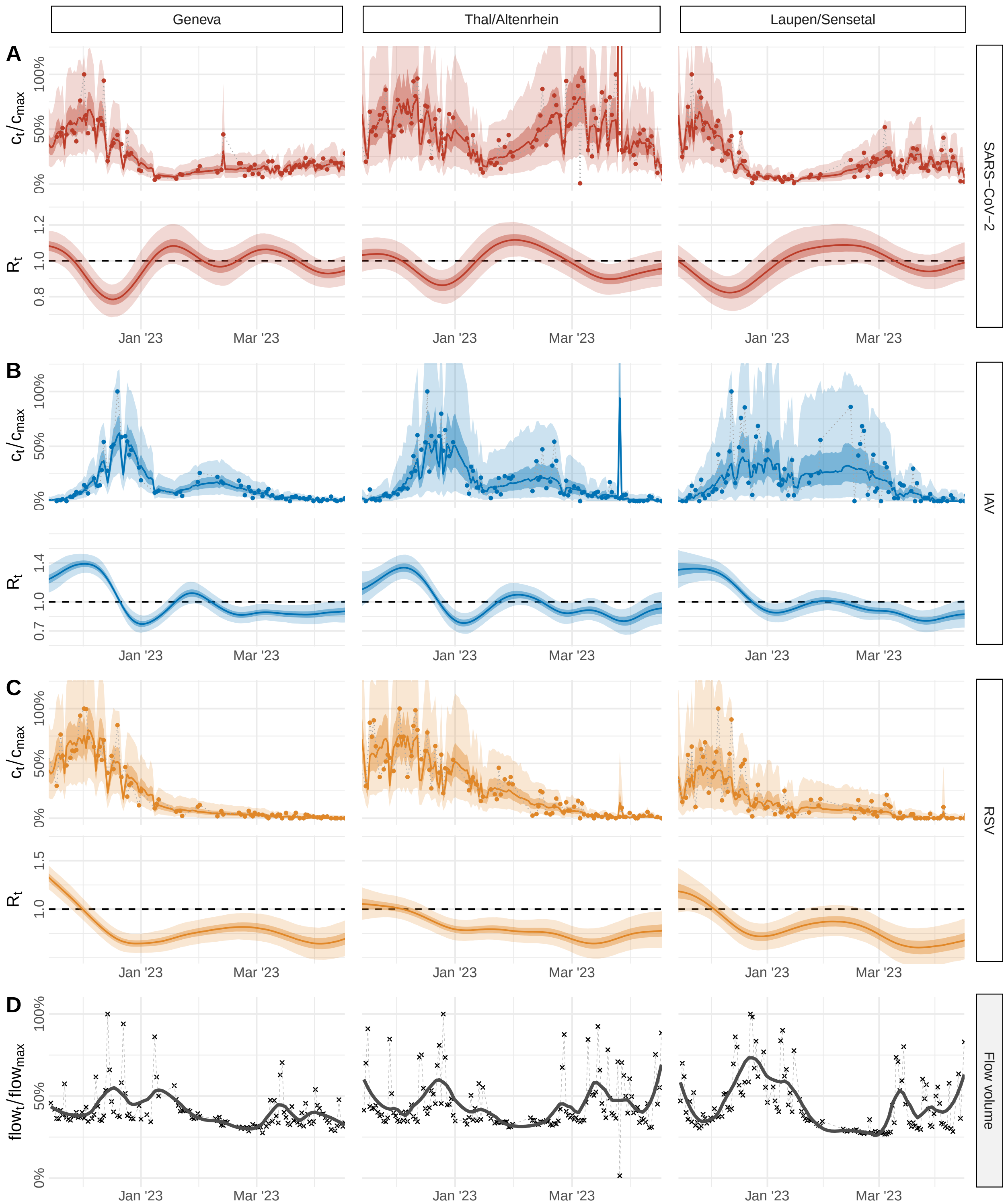

Supplementary Fig. 34. R_t **estimates for additional catchments: Geneva, Thal, and Laupen, winter season 2022/23.** (A-C) Relative pathogen concentrations (top) and estimates of the effective reproduction number R_t (bottom) for SARS-CoV-2, influenza A virus (IAV), and respiratory syncytial virus (RSV) based on longitudinal samples from the wastewater catchments of Geneva (454 000 persons), Thal / Altenrhein region (64 000 persons), and Laupen / Sensetal region (62 000 persons) during the winter season 2022/23. Shown are measured pathogen concentrations (dots), and the posterior median (lines) and 50% and 95% credible intervals (dark and light bands) of predicted concentrations and estimated R_t, respectively. (D) Relative daily flow at the sampled wastewater treatment plant of each catchment. Lines show a smooth trend estimated using LOESS with a 4-week window. Source data are provided as a Source Data file.

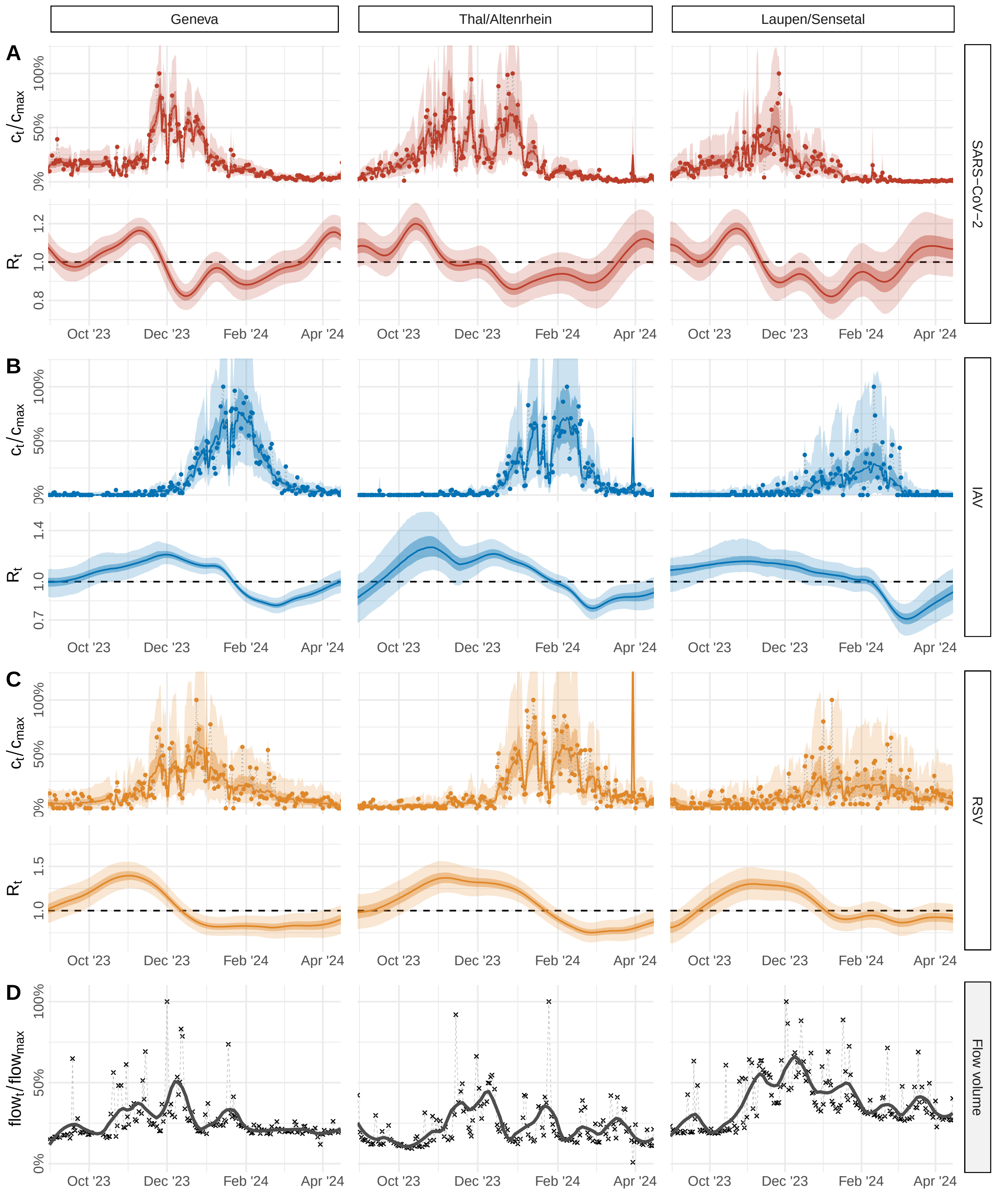

Supplementary Fig. 35. R_t **estimates for additional catchments: Geneva, Thal, and Laupen, winter season 2023/24.** (A-C) Relative pathogen concentrations (top) and estimates of the effective reproduction number R_t (bottom) for SARS-CoV-2, influenza A virus (IAV), and respiratory syncytial virus (RSV) based on longitudinal samples from the wastewater catchments of Geneva (454 000 persons), Thal / Altenrhein region (64 000 persons), and Laupen / Sensetal region (62 000 persons) during the winter season 2023/24. Shown are measured pathogen concentrations (dots), and the posterior median (lines) and 50% and 95% credible intervals (dark and light bands) of predicted concentrations and estimated R_t, respectively. (D) Relative daily flow at the sampled wastewater treatment plant of each catchment. Lines show a smooth trend estimated using LOESS with a 4-week window. Source data are provided as a Source Data file.

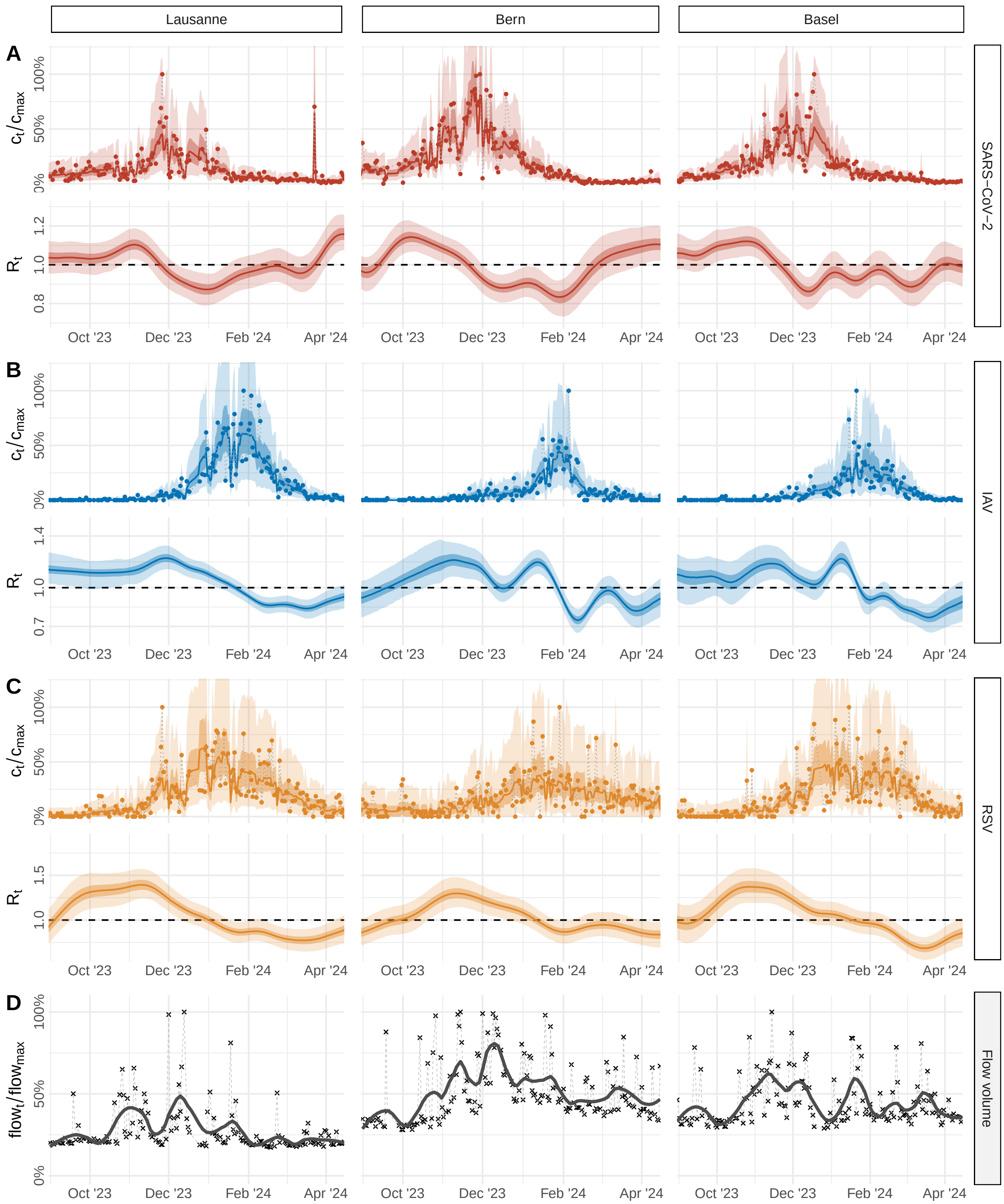

Supplementary Fig. 36. R_t **estimates for additional catchments: Lausanne, Bern, and Basel, winter season 2023/24.** (A-C) Relative pathogen concentrations (top) and estimates of the effective reproduction number R_t (bottom) for SARS-CoV-2, influenza A virus (IAV), and respiratory syncytial virus (RSV) based on longitudinal samples from the wastewater catchments of Lausanne (240 000 persons), Bern (225 000 persons), and Basel (268 000 persons) during the winter season 2023/24. Shown are measured pathogen concentrations (dots), and the posterior median (lines) and 50% and 95% credible intervals (dark and light bands) of predicted concentrations and estimated R_t, respectively. (D) Relative daily flow at the sampled wastewater treatment plant of each catchment. Lines show a smooth trend estimated using LOESS with a 4-week window. Source data are provided as a Source Data file.

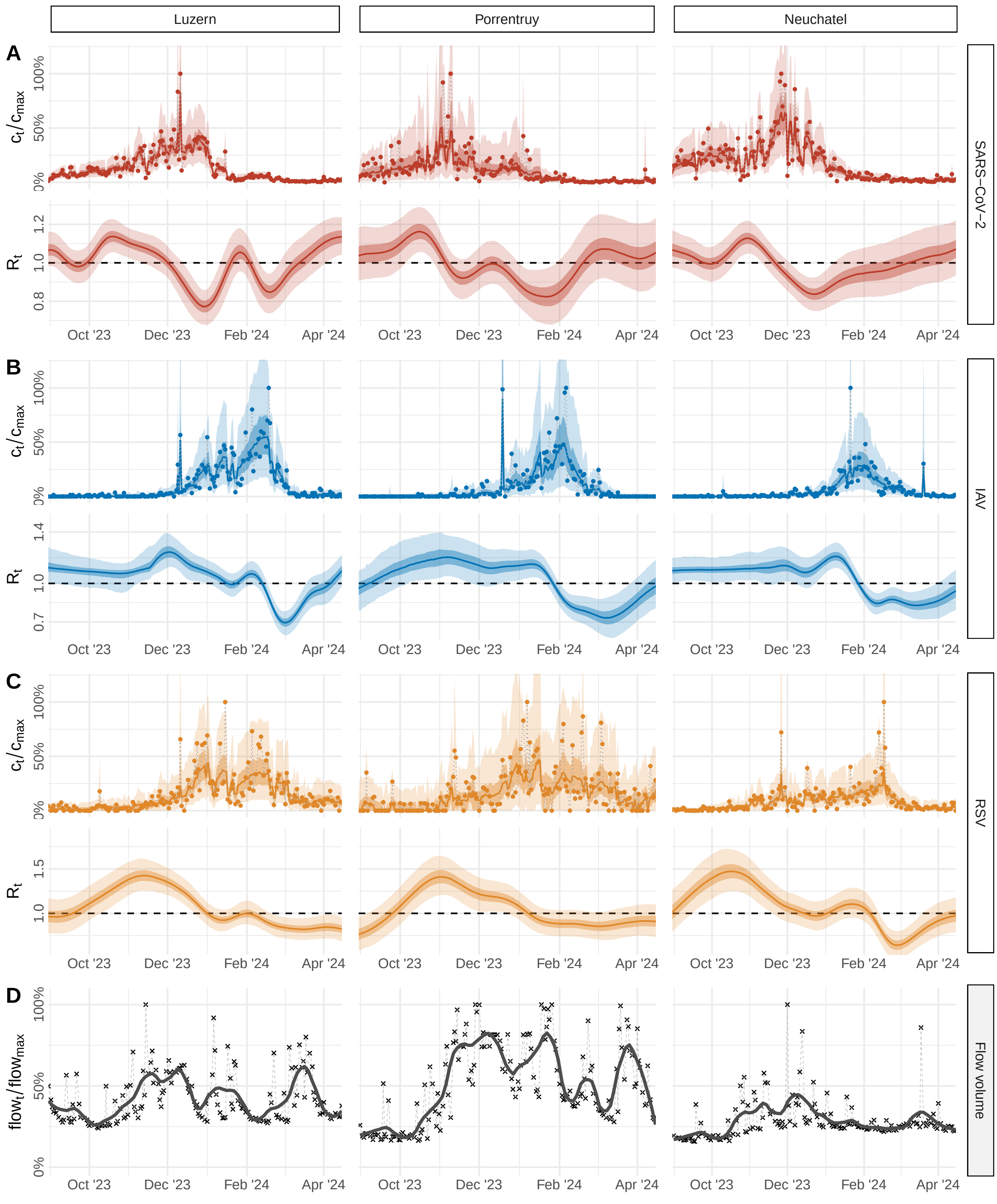

Supplementary Fig. 37. R_t **estimates for additional catchments: Luzern, Porrentruy, and Neuchatel, winter season 2023/24.** (A-C) Relative pathogen concentrations (top) and estimates of the effective reproduction number R_t (bottom) for SARS-CoV-2, influenza A virus (IAV), and respiratory syncytial virus (RSV) based on longitudinal samples from the wastewater catchments of Luzern (179 000 persons), Porrentruy (17 000 persons), and Neuchatel (41 000 persons) during the winter season 2023/24. Shown are measured pathogen concentrations (dots), and the posterior median (lines) and 50% and 95% credible intervals (dark and light bands) of predicted concentrations and estimated R_t, respectively. (D) Relative daily flow at the sampled wastewater treatment plant of each catchment. Lines show a smooth trend estimated using LOESS with a 4-week window. Source data are provided as a Source Data file.

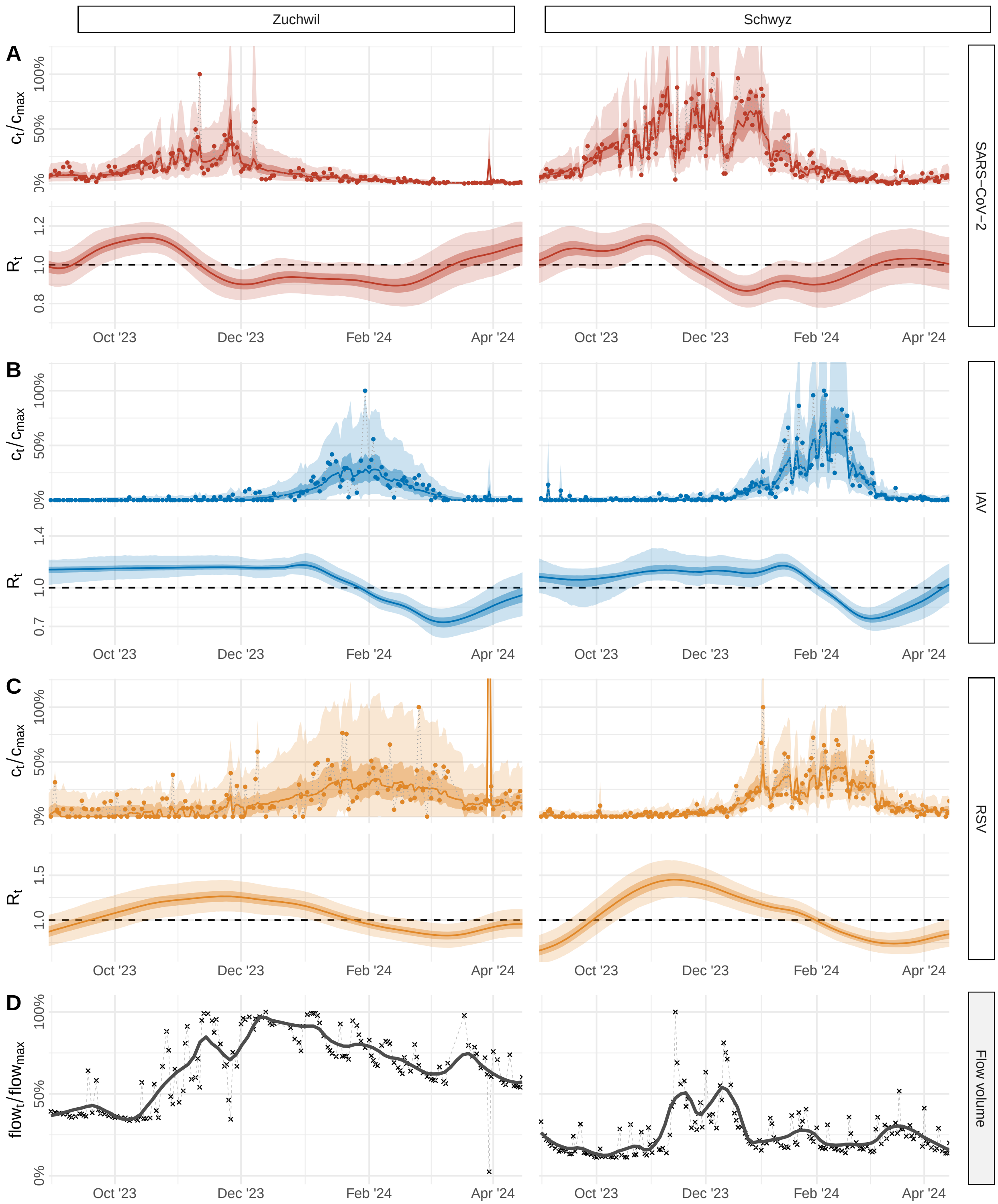

Supplementary Fig. 38. R_t estimates for additional catchments: Zuchwil and Schwyz, winter season 2023/24. (A-C) Relative pathogen concentrations (top) and estimates of the effective reproduction number R_t (bottom) for SARS-CoV-2, influenza A virus (IAV), and respiratory syncytial virus (RSV) based on longitudinal samples from the wastewater catchments of Zuchwil (96 000 persons) and Schwyz (31 000 persons) during the winter season 2023/24. Shown are measured pathogen concentrations (dots), and the posterior median (lines) and 50% and 95% credible intervals (dark and light bands) of predicted concentrations and estimated R_t, respectively. (D) Relative daily flow at the sampled wastewater treatment plant of each catchment. Lines show a smooth trend estimated using LOESS with a 4-week window. Source data are provided as a Source Data file.

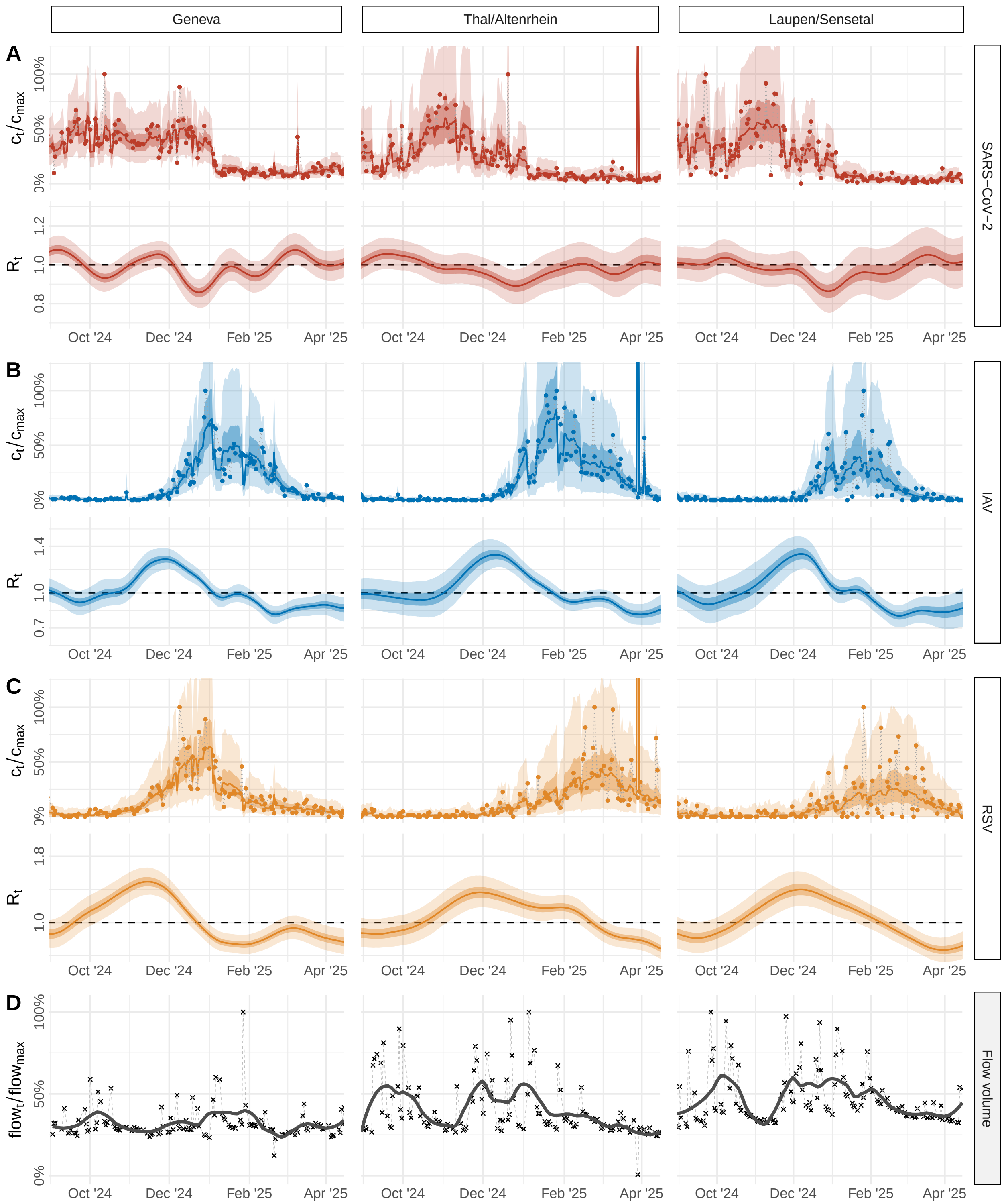

Supplementary Fig. 39. R_t estimates for additional catchments: Geneva, Thal, and Laupen, winter season 2024/25. (A-C) Relative pathogen concentrations (top) and estimates of the effective reproduction number R_t (bottom) for SARS-CoV-2, influenza A virus (IAV), and respiratory syncytial virus (RSV) based on longitudinal samples from the wastewater catchments of Geneva (454 000 persons), Thal / Altenrhein region (64 000 persons), and Laupen / Sensetal region (62 000 persons) during the winter season 2024/25. Shown are measured pathogen concentrations (dots), and the posterior median (lines) and 50% and 95% credible intervals (dark and light bands) of predicted concentrations and estimated R_t, respectively. (D) Relative daily flow at the sampled wastewater treatment plant of each catchment. Lines show a smooth trend estimated using LOESS with a 4-week window. Source data are provided as a Source Data file.

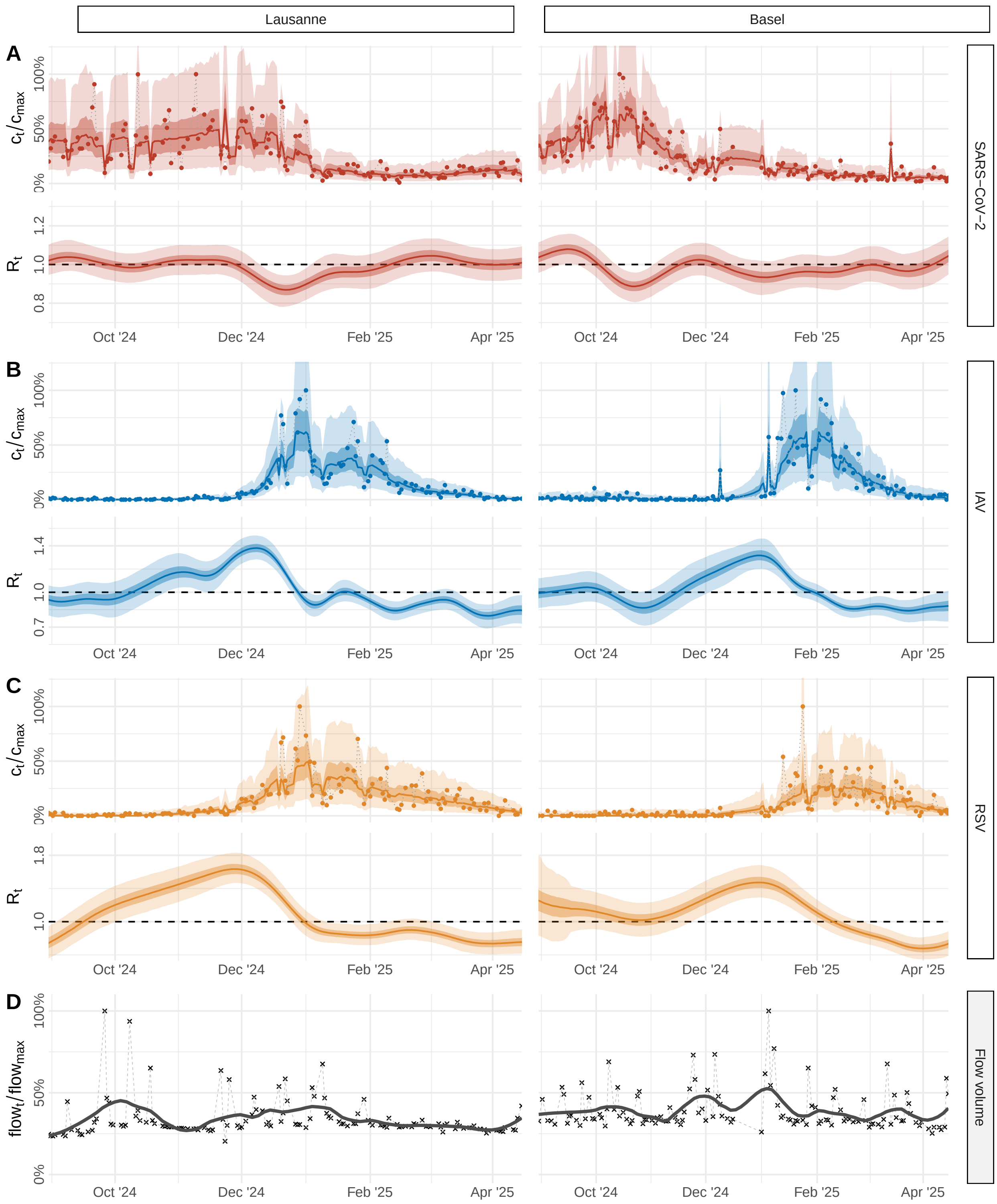

Supplementary Fig. 40. R_t **estimates for additional catchments: Lausanne and Basel, winter season 2024/25.** (A-C) Relative pathogen concentrations (top) and estimates of the effective reproduction number R_t (bottom) for SARS-CoV-2, influenza A virus (IAV), and respiratory syncytial virus (RSV) based on longitudinal samples from the wastewater catchments of Lausanne (240 000 persons) and Basel (268 000 persons) during the winter season 2024/25. Shown are measured pathogen concentrations (dots), and the posterior median (lines) and 50% and 95% credible intervals (dark and light bands) of predicted concentrations and estimated R_t, respectively. (D) Relative daily flow at the sampled wastewater treatment plant of each catchment. Lines show a smooth trend estimated using LOESS with a 4-week window. Source data are provided as a Source Data file.

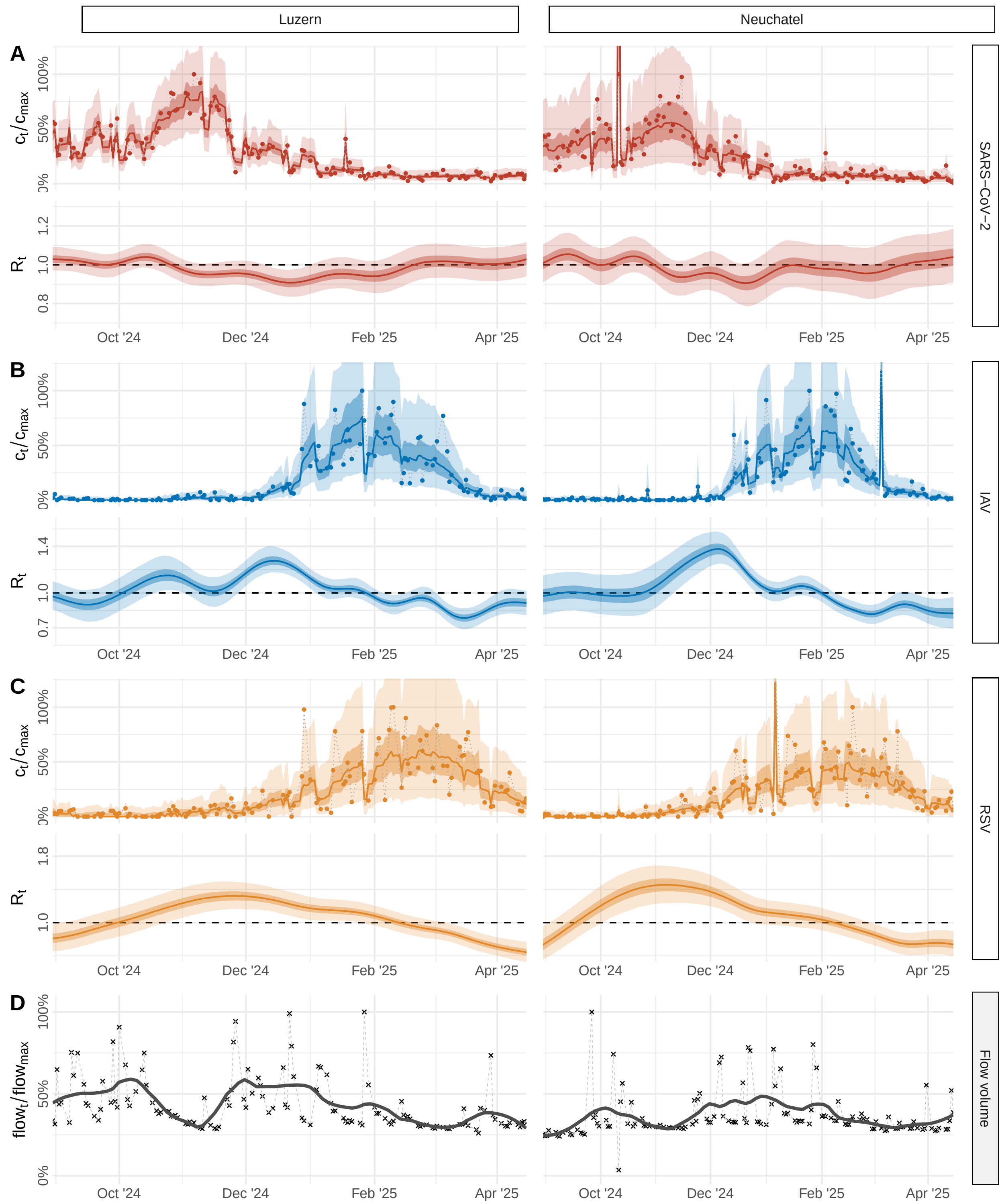

Supplementary Fig. 41. R_t **estimates for additional catchments: Luzern and Neuchatel, winter season 2024/25.** (A-C) Relative pathogen concentrations (top) and estimates of the effective reproduction number R_t (bottom) for SARS-CoV-2, influenza A virus (IAV), and respiratory syncytial virus (RSV) based on longitudinal samples from the wastewater catchments of Luzern (179 000 persons) and Neuchatel (41 000 persons) during the winter season 2024/25. Shown are measured pathogen concentrations (dots), and the posterior median (lines) and 50% and 95% credible intervals (dark and light bands) of predicted concentrations and estimated R_t, respectively. (D) Relative daily flow at the sampled wastewater treatment plant of each catchment. Lines show a smooth trend estimated using LOESS with a 4-week window. Source data are provided as a Source Data file.

Supplementary References

1. Huisman, J. S. *et al.* Wastewater-Based Estimation of the Effective Reproductive Number of SARS-CoV-2. *Environmental Health Perspectives* **130**, 057011 (2022).

2. Nadeau, S. *et al.* Influenza transmission dynamics quantified from RNA in wastewater in Switzerland. *Swiss Medical Weekly* **154**, 3503 (2024).

3. Lison, A., Julian, T. R. & Stadler, T. Improving inference from reported concentrations in environmental surveillance by modeling the statistical features of digital pcr. *ACS ES&T Water* (2026).

4. Asmussen, S., Jensen, J. L. & Rojas-Nandayapa, L. On the Laplace Transform of the Lognormal Distribution. *Methodology and Computing in Applied Probability* **18**, 441–458 (2016).

5. Corless, R. M., Gonnet, G. H., Hare, D. E. G., Jeffrey, D. J. & Knuth, D. E. On the LambertW function. *Advances in Computational Mathematics* **5**, 329–359 (1996).

6. Cori, A., Ferguson, N. M., Fraser, C. & Cauchemez, S. A new framework and software to estimate time-varying reproduction numbers during epidemics. *American Journal of Epidemiology* **178**, 1505–1512 (2013).

7. Cavany, S. *et al.* Inferring SARS-CoV-2 RNA shedding into wastewater relative to the time of infection. *Epidemiology & Infection* **150**, e21 (2022).

8. Wannigama, D. L. *et al.* Increased faecal shedding in SARS-CoV-2 variants BA.2.86 and JN.1. *The Lancet Infectious Diseases* **24**, e348–e350 (2024).

9. Xu, X. *et al.* Assessing changes in incubation period, serial interval, and generation time of SARS-CoV-2 variants of concern: A systematic review and meta-analysis. *BMC Medicine* **21**, 374 (2023).

10. Carrat, F. *et al.* Time Lines of Infection and Disease in Human Influenza: A Review of Volunteer Challenge Studies. *American Journal of Epidemiology* **167**, 775–785 (2008).

11. Otomaru, H. *et al.* Risk of Transmission and Viral Shedding From the Time of Infection for Respiratory Syncytial Virus in Households. *American Journal of Epidemiology* **190**, 2536–2543 (2021).

12. Park, S. W. *et al.* Inferring the differences in incubation-period and generation-interval distributions of the Delta and Omicron variants of SARS-CoV-2. *Proceedings of the National Academy of Sciences* **120**, e2221887120 (2023).

13. te Beest, D. E., Wallinga, J., Donker, T. & van Boven, M. Estimating the Generation Interval of Influenza A (H1N1) in a Range of Social Settings. *Epidemiology* **24**, 244 (2013).

14. Vink, M. A., Bootsma, M. C. J. & Wallinga, J. Serial Intervals of Respiratory Infectious Diseases: A Systematic Review and Analysis. *American Journal of Epidemiology* **180**, 865–875 (2014).

15. Crowcroft, N. S. *et al.* Respiratory syncytial virus infection in infants admitted to paediatric intensive care units in London, and in their families. *European Journal of Pediatrics* **167**, 395–399 (2008).

16. Federal Office of Public Health FOPH. Infectious Diseases Dashboard (IDD). Website (2024). https://idd.bag.admin.ch. Accessed 6 August 2025.

17. Richard, A. *et al.* Grippenet: A New Tool for the Monitoring, Risk-Factor and Vaccination Coverage Analysis of Influenza-Like Illness in Switzerland. *Vaccines* **8**, 343 (2020).

18. McLeod, R. E. Outlier detection in digital PCR wastewater time series data (2024). Master thesis, ETH Zurich. https://doi.org/10.3929/ethz-b-000702298. Accessed 3 February 2026.

19. Bertels, X. *et al.* Factors influencing SARS-CoV-2 RNA concentrations in wastewater up to the sampling stage: A systematic review. *Science of The Total Environment* **820**, 153290 (2022).

20. Wade, M. J. *et al.* Understanding and managing uncertainty and variability for wastewater monitoring beyond the pandemic: Lessons learned from the United Kingdom national COVID-19 surveillance programmes. *Journal of Hazardous Materials* **424**, 127456 (2022).

21. Parag, K. V., Thompson, R. N. & Donnelly, C. A. Are epidemic growth rates more informative than reproduction numbers? *Journal of the Royal Statistical Society: Series A (Statistics in Society)* **185**, S5–S15 (2022).

22. Stan development team. Stan Modeling Language Users Guide and Reference Manual, Version 2.35 (2024). https://mc-stan.org/docs/2_34/reference-manual/index.html. Accessed 29 July 2024.

23. Banholzer, N. *et al.* Estimating the effects of non-pharmaceutical interventions on the number of new infections with COVID-19 during the first epidemic wave. *PLOS ONE* **16**, e0252827 (2021).

24. Flaxman, S. *et al.* Estimating the effects of non-pharmaceutical interventions on COVID-19 in Europe. *Nature* **584**, 257–261 (2020).

25. Lison, A., Abbott, S., Huisman, J. & Stadler, T. Generative Bayesian modeling to nowcast the effective reproduction number from line list data with missing symptom onset dates. *PLOS Computational Biology* **20**, e1012021 (2024).

26. Teh, Y. W. *et al.* Efficient Bayesian inference of Instantaneous Reproduction Numbers at Fine Spatial Scales, with an Application to Mapping and Nowcasting the Covid-19 Epidemic in British Local Authorities. *Journal of the Royal Statistical Society Series A: Statistics in Society* **185**, S65–S85 (2022).

27. Riutort-Mayol, G., Bürkner, P.-C., Andersen, M. R., Solin, A. & Vehtari, A. Practical Hilbert space approximate Bayesian Gaussian processes for probabilistic programming. *Statistics and Computing* **33**, 17 (2022).

28. Solin, A. & Särkkä, S. Hilbert space methods for reduced-rank Gaussian process regression. *Statistics and Computing* **30**, 419–446 (2020).

29. Abbott, S. *et al.* Estimating the time-varying reproduction number of SARS-CoV-2 using national and subnational case counts. *Wellcome Open Research* **5**, 112 (2020).

30. Betancourt, M. A Conceptual Introduction to Hamiltonian Monte Carlo. Preprint at https://doi.org/10.48550/arXiv.1701.02434 (2018).

31. Betancourt, M. Diagnosing Suboptimal Cotangent Disintegrations in Hamiltonian Monte Carlo. Preprint at https://doi.org/10.48550/arXiv.1604.00695 (2016).

32. Stan Development Team. Runtime warnings and convergence problems (2022). https://mc-stan.org/misc/warnings.html. Accessed 18 December 2024.

33. Gelman, A., Rubin, D. B. *et al.* Inference from iterative simulation using multiple sequences. *Statistical Science* **7**, 457–472 (1992).

34. Geyer, C. in *Introduction to Markov Chain Monte Carlo* 0 edn, (eds Brooks, S., Gelman, A., Jones, G. & Meng, X.-L.) *Handbook of Markov Chain Monte Carlo* 3–48 (Chapman and Hall/CRC, 2011).

35. Statistik Stadt Zürich. Pendler*innen nach wohnort und arbeitsort, 2023. Tech. Rep., Stadt Zürich [Website] (2023). Available from https://www.stadt-zuerich.ch/de/politik-und-verwaltung/statistik-und-daten/daten/mobilitaet/pendelnde.html#publikationen.

36. Munday, J. D. *et al.* Post-sampling degradation of viral RNA in wastewater impacts the quality of PCR-based concentration estimates. Preprint at https://doi.org/10.1101/2025.06.06.658243 (2025).
