# Supplementary material for: Real-time estimation of pathogen transmission dynamics from wastewater
Source: Nat Commun. 2026 Jul 11;17:8563. doi: 10.1038/s41467-026-75380-3 (PMC13483917; doi:10.1038/s41467-026-75380-3)
Supplement: Supplementary file 2 [file 41467_2026_75380_MOESM2_ESM.pdf]

Description of Additional Supplementary Files

Supplementary Movie 1: Real-time Rt estimation and concentration forecasting for SARS-CoV-2 in Zurich, winter season 2023/24.
Description: Top panel shows concentration forecasts and observed concentrations (stars), rescaled to median flow. Bottom panel shows real-time estimates and 14-day-ahead projections of Rt, along with retrospective Rt (in gray). Lines indicate posterior medians; shaded bands represent 50% (dark) and 95% (light) credible intervals.

Supplementary Movie 2: Real-time Rt estimation and concentration forecasting for Influenza A in Zurich, winter season 2023/24.
Description: Top panel shows concentration forecasts and observed concentrations (stars), rescaled to median flow. Bottom panel shows real-time estimates and 14-day-ahead projections of Rt, along with retrospective Rt (in gray). Lines indicate posterior medians; shaded bands represent 50% (dark) and 95% (light) credible intervals.

Supplementary Movie 3: Real-time Rt estimation and concentration forecasting for RSV in Zurich, winter season 2023/24.
Description: Top panel shows concentration forecasts and observed concentrations (stars), rescaled to median flow. Bottom panel shows real-time estimates and 14-day-ahead projections of Rt, along with retrospective Rt (in gray). Lines indicate posterior medians; shaded bands represent 50% (dark) and 95% (light) credible intervals.
