# Supplementary material for: Real-time estimation of pathogen transmission dynamics from wastewater
Source: Nat Commun. 2026 Jul 11;17:8563. doi: 10.1038/s41467-026-75380-3 (PMC13483917; doi:10.1038/s41467-026-75380-3)
Supplement: Supplementary file 3 [file 41467_2026_75380_MOESM6_ESM.pdf]

nature portfolio

Corresponding author(s): Adrian Lison

Last updated by author(s): 18.06.2026

# Reporting Summary

Nature Portfolio wishes to improve the reproducibility of the work that we publish. This form provides structure for consistency and transparency in reporting. For further information on Nature Portfolio policies, see our Editorial Policies and the Editorial Policy Checklist.

## Statistics

For all statistical analyses, confirm that the following items are present in the figure legend, table legend, main text, or Methods section.

| n/a | Confirmed | |
|---|---|---|
| ☐ | ☒ | The exact sample size (*n*) for each experimental group/condition, given as a discrete number and unit of measurement |
| ☐ | ☒ | A statement on whether measurements were taken from distinct samples or whether the same sample was measured repeatedly |
| ☒ | ☐ | The statistical test(s) used AND whether they are one- or two-sided<br>*Only common tests should be described solely by name; describe more complex techniques in the Methods section.* |
| ☐ | ☒ | A description of all covariates tested |
| ☐ | ☒ | A description of any assumptions or corrections, such as tests of normality and adjustment for multiple comparisons |
| ☐ | ☒ | A full description of the statistical parameters including central tendency (e.g. means) or other basic estimates (e.g. regression coefficient) AND variation (e.g. standard deviation) or associated estimates of uncertainty (e.g. confidence intervals) |
| ☒ | ☐ | For null hypothesis testing, the test statistic (e.g. *F*, *t*, *r*) with confidence intervals, effect sizes, degrees of freedom and *P* value noted<br>*Give P values as exact values whenever suitable.* |
| ☐ | ☒ | For Bayesian analysis, information on the choice of priors and Markov chain Monte Carlo settings |
| ☐ | ☒ | For hierarchical and complex designs, identification of the appropriate level for tests and full reporting of outcomes |
| ☒ | ☐ | Estimates of effect sizes (e.g. Cohen's *d*, Pearson's *r*), indicating how they were calculated |

*Our web collection on statistics for biologists contains articles on many of the points above.*

## Software and code

Policy information about availability of computer code

| | |
|---|---|
| Data collection | This study used existing open data, no specialized software was used for data collection. |
| Data analysis | All analyses were conducted in R v4.5.2. Statistical models were implemented in stan and fitted using cmdstan v2.34.1 via cmdstanr v0.9.0. The EpiSewer stan code and modeling interface are available as an open-source R package at https://github.com/adrian-lison/EpiSewer (v0.0.4). R packages dplyr v1.1.4, tidyr v1.3.1 and data.table v1.17.8 were used for data processing, ggplot2 v4.0.0 for plotting, and targets v1.11.4 for pipeline orchestration. All R packages with exact versions are documented in the study repository at https://github.com/adrian-lison/EpiSewer-study and can be installed via the renv package. |

For manuscripts utilizing custom algorithms or software that are central to the research but not yet described in published literature, software must be made available to editors and reviewers. We strongly encourage code deposition in a community repository (e.g. GitHub). See the Nature Portfolio guidelines for submitting code & software for further information.

## Data

Policy information about availability of data

All manuscripts must include a data availability statement. This statement should provide the following information, where applicable:

- Accession codes, unique identifiers, or web links for publicly available datasets
- A description of any restrictions on data availability
- For clinical datasets or third party data, please ensure that the statement adheres to our policy

This work is based on a secondary analysis of open wastewater data provided by Eawag, Swiss Federal Institute of Aquatic Science and Technology, under a CC BY 4.0 license. The data are publicly available at https://github.com/adrian-lison/EpiSewer-study and from the public wastewater dashboard at https://wise.ethz.ch. Source data are provided with this paper.

## Research involving human participants, their data, or biological material

Policy information about studies with human participants or human data. See also policy information about sex, gender (identity/presentation), and sexual orientation and race, ethnicity and racism.

| | |
|---|---|
| Reporting on sex and gender | n/a |
| Reporting on race, ethnicity, or other socially relevant groupings | n/a |
| Population characteristics | n/a |
| Recruitment | n/a |
| Ethics oversight | n/a |

Note that full information on the approval of the study protocol must also be provided in the manuscript.

# Field-specific reporting

Please select the one below that is the best fit for your research. If you are not sure, read the appropriate sections before making your selection.

☒ Life sciences ☐ Behavioural & social sciences ☐ Ecological, evolutionary & environmental sciences

For a reference copy of the document with all sections, see nature.com/documents/nr-reporting-summary-flat.pdf

# Life sciences study design

All studies must disclose on these points even when the disclosure is negative.

| | |
|---|---|
| Sample size | This study analyzes data produced in the context of a national wastewater monitoring program across three respiratory virus seasons in Switzerland, i.e. from the beginning of November 2022 to the end of May 2025. The selection of sampling sites and number of wastewater samples processed at each site was determined in coordination with public health authorities based on both representativeness and cost considerations. Generally, the program aimed for a sufficient geographical spread of sampling sites across the country and, based on previous subsampling experiments of daily measurement data, a minimum sampling frequency of three samples per week. During the respiratory virus season 2022/23, samples were collected since November 2022 from 6 wastewater treatment plants distributed across major cities in Switzerland and analyzed with an average frequency of 5 days per week, i. e. all weekdays except for Wednesdays and Fridays. During the season 2023/24, samples were collected from 14 wastewater treatment plants and analyzed with an average frequency of 5 days per week, using a biweekly sampling scheme that varied between batch groups. During the season 2024/2025, sampling had to be slightly down-scaled due to cost constraints, i.e. samples were collected from 10 wastewater treatment plants and analyzed with an average frequency of 4 days per week (since June 2024), again using a biweekly sampling scheme that varied between batch groups. Detailed reports on the sampling scheme for each individual treatment plant are provided in the Supporting Information. |
| Data exclusions | All measurements that passed laboratory quality control were used in the study. Quality control criteria are described in detail in Nadeau et al. 2024 and Pitton et al. 2024 (referenced in manuscript); in brief, assay positive controls must have signal, negative control must have fewer than three positive partitions, there must be a minimum of 15’000 analyzable droplets, and PCR inhibition must be below 40%. Highly inhibited samples were re-run at a higher dilution. |
| Replication | This study did not involve laboratory experiments. All analyses are based on previously published wastewater data, and replication of the PCR assays used to produce these data is described in the corresponding publications (Nadeau et al., Swiss Medical Weekly, 2024; Pitton et al., Nature Water, 2025), also cited in the Methods section. The reproducibility of the computational modeling approach developed in this work is ensured through open-source code, full documentation of model parameters and priors, and out-of-sample forecast validation across multiple pathogens (SARS-CoV-2, IAV, RSV), multiple catchments (with small, medium, large population sizes), and three consecutive winter seasons, with consistent results across these settings. |

| | |
|---|---|
| Randomization | Wastewater samples were stratified by sampling location to estimate transmission dynamics for individual catchment populations across Switzerland. Within each location, samples were purely longitudinal and not further grouped. Randomization was not relevant to this observational, population-level study. |
| Blinding | Blinding was not relevant to this observational, population-level study. |

# Reporting for specific materials, systems and methods

We require information from authors about some types of materials, experimental systems and methods used in many studies. Here, indicate whether each material, system or method listed is relevant to your study. If you are not sure if a list item applies to your research, read the appropriate section before selecting a response.

## Materials & experimental systems

| n/a | Involved in the study |
|---|---|
| ☒ | ☐ Antibodies |
| ☒ | ☐ Eukaryotic cell lines |
| ☒ | ☐ Palaeontology and archaeology |
| ☒ | ☐ Animals and other organisms |
| ☒ | ☐ Clinical data |
| ☒ | ☐ Dual use research of concern |
| ☒ | ☐ Plants |

## Methods

| n/a | Involved in the study |
|---|---|
| ☒ | ☐ ChIP-seq |
| ☒ | ☐ Flow cytometry |
| ☒ | ☐ MRI-based neuroimaging |

## Plants

| | |
|---|---|
| Seed stocks | *Report on the source of all seed stocks or other plant material used. If applicable, state the seed stock centre and catalogue number. If plant specimens were collected from the field, describe the collection location, date and sampling procedures.* |
| Novel plant genotypes | *Describe the methods by which all novel plant genotypes were produced. This includes those generated by transgenic approaches, gene editing, chemical/radiation-based mutagenesis and hybridization. For transgenic lines, describe the transformation method, the number of independent lines analyzed and the generation upon which experiments were performed. For gene-edited lines, describe the editor used, the endogenous sequence targeted for editing, the targeting guide RNA sequence (if applicable) and how the editor was applied.* |
| Authentication | *Describe any authentication procedures for each seed stock used or novel genotype generated. Describe any experiments used to assess the effect of a mutation and, where applicable, how potential secondary effects (e.g. second site T-DNA insertions, mosiacism, off-target gene editing) were examined.* |
