# Supplementary material for: Real-time estimation of pathogen transmission dynamics from wastewater
Source: Nat Commun. 2026 Jul 11;17:8563. doi: 10.1038/s41467-026-75380-3 (PMC13483917; doi:10.1038/s41467-026-75380-3)
Supplement: Supplementary file 4 [file 41467_2026_75380_MOESM7_ESM.pdf]

nature portfolio

Peer Review File

Real-time estimation of pathogen transmission dynamics from wastewater

Corresponding Author: Dr Adrian Lison

This file contains all reviewer reports in order by version, followed by all author rebuttals in order by version.

Version 0:

Reviewer comments:

Reviewer #1

(Remarks to the Author)
The authors introduce a hierarchical Bayesian semi-mechanistic model to infer the time-varying reproduction number (along with other quantities) from wastewater signals. The model accounts for various elements affecting wastewater signals, like outlier spikes of high concentration and false non-detections. The approach is applied to 6–14 treatment plants in Switzerland, for SARS-CoV-2, Influenza, and RSV across three seasons. The authors perform short-term forecasts of concentration measurements to evaluate the method, showing robust coverage and low bias across pathogens, locations, and subsampling conditions.

This is an impressive piece of work. The paper is technically sound as far as I could tell, and the authors were very thorough in their analyses. The Bayesian inference framework is robust, deals effectively with outliers (and the overall noise of wastewater signals), is grounded in the literature, and built from principled mechanisms at play influencing wastewater signals, from disease transmission to dPCR measurement. This study provides a blueprint for the usage of wastewater signals in statistical inference and forecasts.

I particularly appreciated the out-of-sample validation results for Rt estimates based on short-term forecasts: the fact that we don't have access to ground truth for Rt estimates makes it especially challenging to advocate for a particular method over another; this type of validation grounds Rt estimates in rigorous and quantifiable metrics, through which models can be compared and thoroughly evaluated.

I also appreciated the extensive sensitivity analyses performed in the SI. As I was reading about the model, I was getting concerned about the increasing complexity of the hierarchical Bayesian framework, as the authors aim to model every mechanism from transmission, to shedding, to data measurement, with detailed priors along the way. This makes one wonder how much the results shown in this paper hinge on the details and fine-tuning. But the sensitivity analyses in SI section E do reassure on the robustness of the results (E3 for instance showing a simpler model wouldn't hurt performance). I think as future work, coming up with a distilled version of this inference pipeline, one that verifies and encapsulates only the core, necessary components, would be useful and improve reusability by the community.

A few more specific comments and questions:

- In the methods section, when the authors introduce the posterior evaluation, I think it should be made clearer what other "parameters" are being inferred jointly with Rt (now included as ...). The idea is to help reproducibility and also give a sense of the computational cost of such inference (any runtime guidelines for these estimates?).
- When calibrating the distribution capturing outliers, they used a fixed heavy-tailed Fréchet distribution. But the catchment areas vary in size from 64,000 to 471,000; have the authors found that this variation does not significantly affect the outlier distribution?
- Is there a rationale for why the outlier term is only positive and additive? When looking at concentration signals, it seems that low-value data points far from the main trend are prevalent as well, unless those are correctly captured by the base model. Is there a particular reason also for preferring an additive model for outliers instead of a multiplicative one?
- Table S1: I'm assuming Normal+ refers to a positive truncated normal distribution? Please specify.

(Remarks on code availability)

Reviewer #2

(Remarks to the Author)
In this work, the authors developed a framework Episewer to infer the pathogen transmission dynamics, specifically the effective reproduction number from wastewater testing data. The authors showed this Bayesian based framework can successfully estimate the real time Rt based on measured viral concentrations in wastewater with quantified uncertainty for SARS-CoV-2, influenza A, and RSV. They tested the framework using data from 3 wastewater treatment plants during the 2023-2024. My major comments are in the method section, some of which are incorrect based on outdated data, and need extra details to justify. For the inferred Rt, the work also lack evidence to validate the effectiveness of the approach.

Specific comments:

1. Viral shedding load modeling issues: One important player in the wastewater data is the viral shedding load. However, the approach for shedding load modeling is problematic. For example, for IAV, the authors used a gamma distribution with normal priors, μ^load and variation νζ based on literature Nadeau et al., 1998. Several issues: First, the paper Nadeau et al., 1998 measured viral titers, not the viral genome copies, which is typically orders of magnitude larger than viral titers, and this will certainly make the shedding load per person significantly lower, and thus impact the estimation of infectious population size and estimated effective reproduction number.
Second, in equation 3 (line 345), the μ is the average detectable total shedding load per person, but the literature data measured infectious viral concentration, so one missing component is the material load (here can be feces or materials contributed to the sewer system per person per day), and that varies in the population, and the authors need to mention how they incorporate that in to the model. Third, the data are based on nasal swab samples, it may be fine to make assumptions to connect nasal viral load vs stool viral load, but a reasonable justification is needed.

Fourth, the viral shedding can be very different from different variants or strains. Like for influenza, the H1N1 usually have higher shedding in stool, but other influenza types are barely detectable in feces. This applies to SARS-CoV-2, like Omicron's shedding seems much larger than the wild type. The authors need to consider this for a temporal estimation of Rt, especially for these fast evolving virus.

Similar issues apply to the SARS-CoV-2 and RSV shedding load modeling.

2. missing environmental and demographic variation factors. The authors nicely considered the potential variation to the detection results in wastewater. but i think two important ones were missed. First, transit and viral genetic loss in the sewer. For example, in equation 2 (line 332), the expected viral concentration equals the total load divided by the flow. Other factors like retention time, viral degradation, viral loss from biofilms, viral stability…etc. those factors all impact the expected concentration, and can be quite large for different wwtps, and also sewer specific especially for some old ones. Second, the moving population in each sewershed. This variation has not even been considered in the total load shed, but their differences can be very large depends on the location/business of the community, like some places have high outside visitors/travellers.

3. In Line 348, 'I' is described as the number of infected individuals, weighted by overall shedding intensity. But if it is 'infected individuals', then in equation 4 (line 359), it would be incorrect to use the total infected individuals multiplied by the instantaneous effective reproduction number. From equation 5, if I follows a Poisson distribution, I guess it shall be ‘incidence’, not prevalence (infected individuals). This needs clarification.

4. Line 380 assumed 8-17.7 days for the shedding duration for SARS-CoV-2, but that reference is for data in August to November 2020, during the wild type period. But the measured data actually covers 2023 to 2024, during the Omicron period. The authors need to update based on variant data.
Also, in lines 375-385, those descriptions for SARS-CoV-2, RSV, Influenza are not 'shedding load distribution' as described in the paper, it more seems 'shedding duration'.

5. line 47-48, the statement is incorrect. The two literatures cited did not talk about the 'inherent delays between infection and shedding'. Both talked about the delay between infection and testing.

6. There are a dozen papers that reported the Rt estimation from wastewater data as well as R packages developed, as the authors mentioned some in the paper. There are some more like PMID: 39566444, PMID: 39729960. It would be greatly helpful to have a benchmark and the unique strengths for this work shall be discussed.

(Remarks on code availability)

Reviewer #3

(Remarks to the Author)
This is an interesting and elegant model. My primary concern is with the calibration of the shedding per individual. The authors have calibrated this to case incidence data, which raises a question about how these case data were interpreted

and why they weren't included explicitly in the time series analysis.

Figure S16 indicates that Rt is relatively robust to assumptions about shedding, but the absolute incidence is certainly not. As expected, the inferred incidence (which many would consider a more important quantity than Rt to get right in absolute value) is negatively correlated with the assumed shedding. The estimated incidence in Figure S16 varies over orders of magnitude.

The authors are heavily focussed on the robustness of Rt, which is impressive. But the indeterminate estimates of incidence require more attention here. If the answer is that incidence cannot be reliably estimated without very explicit quantification of individual-level shedding, then that needs to be better explained. If there is bias (e.g. due to under-reporting) in the reported incidence data that the authors use to calibrate their shedding rate per individual, then Figure S16 would indicate that the estimates of absolute incidence cannot be trusted. If the reported incidence values used ARE unbiased, then they could be used to constrain the model. An even better thing to do would be to estimate total incidence and Rt from the reported incidence time series alone and compare those estimates to the waste water-based estimates. The authors indicate in the text (starting in L225) that they don't favor that approach to calibration. Instead they validate based on forecasts of wastewater concentration and Rt; the former works impressively, but forecasts a quantity that isn't operationally useful without translation into cases, or similar.

I would like to see the authors do some validation either to observed time series of cases (illustrating whether incidence is estimable in if shedding is mis-specified) or to simulated data where all values are known. The latter would be tractable if the authors don't feel confident in the application of standard methods to the likely underreported time series are valid. Finally, if this methods works for Rt, but not for incidence, then that needs to be more clearly disclosed in the limitations of the study.

(Remarks on code availability)

Version 1:

Reviewer comments:

Reviewer #1

(Remarks to the Author)
The authors have addressed all my comments, providing further details in the supporting information document about parameter estimation, outlier modeling, and other minor remarks. I recommend publication without delay.

(Remarks on code availability)

Reviewer #2

(Remarks to the Author)
I am thankful for the authors to address most of my comments. While many are just acknowledging the limitations in the discussion, but I understand those are hard questions in the field with complications and unknown parameters that we are not very clear so far.

One point I don't agree with the authors about the comment 2.10, the authors changed their earlier statement to 'Because fecal shedding is distributed over time after infection, wastewater concentrations tend to lag transmission dynamics in the community [34,35]'. I think this is a very strong statement, and not well studied and supported by the two cited papers. The paper by Arts et al. reported time-varying and heterogeneous fecal viral shedding among individuals, and. The paper by Cavany et al. estimated that individual shedding into wastewater peaks on average about 6 days after infection, with a relatively strong overdisperation. Either of the paper suggests that wastewater concentrations tend to lag community infection dynamics.

moreover, the logical link between time-distributed shedding and a community-level lag is weak. In the early phase of an outbreak, wastewater can reflect increases in transmission well and in later phases, wastewater data may not reflect well, but that is likely driven by the aggregation of viral shedding across infected individuals rather than by a true lag in transmission dynamics (see paper https://doi.org/10.1038/s43856-025-00908-5 for reference). So I think wastewater data reflect community infection dynamics in general, unless the virus infection and excretion have an obvious lag or the wastewater testing is not sensitive enough to capture those early sheddings, at least not caused by the time distributed shedding.
fuqing

(Remarks on code availability)

Reviewer #3

(Remarks to the Author)
In general I am satisfied with the authors' response to the reviewers. I still believe some of the language overstates the outcomes.

L 84-97 states "produce Bayesian estimates of ... latent parameters useful for model assessment, e.g. implied infection numbers ...", while it is true these estimate are produced they are not reliable. A more honest statement is that this methods estimates Rt and rt. It also produces estimates and projections of total catchment load. But lumping incidence in to this discussion implies that it is also estimated well, which it is not.

I would encourage the authors to consider changing the wording "transmission dynamics", which is used as a catch-all here. It seems that phrase could be replaced with "effective reproduction number" without affecting the interpretation or value of the work. Of the estimated parameters in table S3, most are not parameters with a meaningful biological interpretation. Those that are relate to rt (from which Rt is derived), It (which the authors indicate is not well estimated), or shedding, which the appears to be strongly constrained by assumptions. The authors don't appear to be presenting results on what was learned about shedding; rather they are using the hierarchical structure of the model to account for uncertainty in shedding dynamics, but not estimating these dynamics, per se. Thus, from the title onwards, "robust real-time estimation of pathogen effective reproduction number form wastewater" would be a more honest accounting of this work. This is not a criticism of the work or the model, which is impressive. If the editor does not agree with this assessment, I will not pursue it further and will support publication pending the small change to the text mentioned above.

(Remarks on code availability)

Open Access This Peer Review File is licensed under a Creative Commons Attribution 4.0 International License, which permits use, sharing, adaptation, distribution and reproduction in any medium or format, as long as you give appropriate credit to the original author(s) and the source, provide a link to the Creative Commons license, and indicate if changes were made.
In cases where reviewers are anonymous, credit should be given to 'Anonymous Referee' and the source.
The images or other third party material in this Peer Review File are included in the article’s Creative Commons license, unless indicated otherwise in a credit line to the material. If material is not included in the article’s Creative Commons license and your intended use is not permitted by statutory regulation or exceeds the permitted use, you will need to obtain permission directly from the copyright holder.
To view a copy of this license, visit https://creativecommons.org/licenses/by/4.0/

Point-by-point response to reviewers

We thank the editor and reviewers for their constructive feedback. Below we address all comments in detail and indicate where changes appear in the revised manuscript.

Additional adjustments

We note two adjustments in addition to the changes requested by the reviewers. First, we improved the parameterization of the dPCR measurement model (see Supplementary Information C.1) to make prior specification more intuitive (e.g., allowing users to specify the maximum number of partitions on a dPCR chip, as reported by manufacturers). Second, we corrected a minor implementation error in the Gaussian process (GP) component that affected the interpretation of the prior we placed on the GP magnitude (see explanation in Supplementary Information D.2). The changes had only minimal effect on inference; we reran all analyses, and all results and conclusions remain qualitatively unchanged.

Reviewer #1

Comment 1.0: The authors introduce a hierarchical Bayesian semi-mechanistic model to infer the time-varying reproduction number (along with other quantities) from wastewater signals. The model accounts for various elements affecting wastewater signals, like outlier spikes of high concentration and false non-detections. The approach is applied to 6–14 treatment plants in Switzerland, for SARS-CoV-2, Influenza, and RSV across three seasons. The authors perform short-term forecasts of concentration measurements to evaluate the method, showing robust coverage and low bias across pathogens, locations, and subsampling conditions.

This is an impressive piece of work. The paper is technically sound as far as I could tell, and the authors were very thorough in their analyses. The Bayesian inference framework is robust, deals effectively with outliers (and the overall noise of wastewater signals), is grounded in the literature, and built from principled mechanisms at play influencing wastewater signals, from disease transmission to dPCR measurement. This study provides a blueprint for the usage of wastewater signals in statistical inference and forecasts.

Response 1.0: Thank you for this positive summary. We highly appreciate your feedback on our work. We have addressed your detailed comments and questions below.

Comment 1.1: I particularly appreciated the out-of-sample validation results for Rt estimates based on short-term forecasts: the fact that we don't have access to ground truth for Rt estimates makes it especially challenging to advocate for a particular method over another; this type of validation grounds Rt estimates in rigorous and quantifiable metrics, through which models can be compared and thoroughly evaluated.

Response 1.1: Thank you very much. We fully agree that out-of-sample validation, as demonstrated in our study, provides a rigorous approach for model evaluation and comparison, particularly when no ground truth for epidemiological parameters is available. As we have argued in our discussion, this type of evaluation is especially meaningful for generative models like EpiSewer, which generate out-of-sample predictions under the same assumptions used for inference, thereby creating a close link between real-time transmission dynamics estimates and short-term forecasts of observations.

Comment 1.2: I also appreciated the extensive sensitivity analyses performed in the SI. As I was reading about the model, I was getting concerned about the increasing complexity of the hierarchical Bayesian framework, as the authors aim to model every mechanism from transmission, to shedding, to data measurement, with detailed priors along the way. This makes one wonder how much the results shown in this paper hinge on the details and fine-tuning. But the sensitivity analyses in SI section E do reassure on the robustness of the results (E3 for instance showing a simpler model wouldn't hurt performance). I think as future work, coming up with a distilled version of this inference pipeline, one that verifies and encapsulates only the core, necessary components, would be useful and improve reusability by the community.

Response 1.2: Thank you for your kind appreciation. We have invested significant effort in assessing the robustness of our model, which we believe is especially important with respect to the modeling of shedding loads, due to the limited clinical data available for many pathogens. We indeed find that transmission dynamic estimates from our model are remarkably robust to different shedding-related assumptions. We also agree that certain components of our shedding model may prove unessential for robust inference of R_t in most settings and could thus be discarded to reduce model complexity. A good example of this is our modeling of individual-level variation in total shedding loads. We have included this component due to evidence of substantial variation in shedding intensity across individuals, but found it to have a negligible impact on transmission dynamic estimates. This suggests that individual-level variation in shedding might also be sufficiently captured by our model of measurement noise, leading to essentially identical R_t estimates (Supplementary Information E.3). However, to discard certain model components confidently, we believe that more extensive testing across various settings and pathogens should be done in future studies. Ideally, this will enable

practical guidelines on when specific components can be omitted from the model without compromising inference. It is worth noting that our R package *EpiSewer* already supports simpler versions of the model through a user-friendly interface; for instance, the user can specify to omit the modeling of individual-level load variation. This empowers users to tailor the model's complexity to their own application and to assess the impact of adding or removing individual components on estimation.

Comment 1.3: A few more specific comments and questions:

- In the methods section, when the authors introduce the posterior evaluation, I think it should be made clearer what other "parameters" are being inferred jointly with Rt (now included as ...). The idea is to help reproducibility and also give a sense of the computational cost of such inference (any runtime guidelines for these estimates?).

Response 1.3: This is a good suggestion. We have updated the formula and manuscript to provide a better overview of the estimated parameters. Note that to limit the number of symbols in the equation, we grouped the parameters into three categories, i.e. measurement parameters (including outliers), shedding parameters, and infection parameters. We describe the specific parameters of each group in detail in the **new Supplementary Table S3**.

Moreover, we added detailed runtime statistics to Supplementary Information D.3 to provide insight into the computational cost of inference (**new Supplementary Fig. 10**). In short, per-chain runtimes ranged from an average of 12.6 min for 4 weeks of data to 85 min for 26 weeks of data (chains can be run in parallel). Importantly, the sampling runtime of EpiSewer largely depends on the amount of data and the infection trajectory implied under our model, which naturally introduces substantial correlation between model parameters (e.g. consecutive R_t values). Runtimes are less influenced by the addition or removal of model components for shedding and measurement. This is a typical phenomenon in Hamiltonian Monte Carlo sampling, where the complexity of the likelihood landscape often plays a larger role for sampling efficiency than the number of parameters [1].

References

[1] Betancourt M. A Conceptual Introduction to Hamiltonian Monte Carlo. arXiv [Preprint]; 2018. Available from https://doi.org/10.48550/arXiv.1701.02434.

Comment 1.4: - When calibrating the distribution capturing outliers, they used a fixed heavy-tailed Fréchet distribution. But the catchment areas vary in size from 64,000 to 471,000; have the authors found that this variation does not significantly affect the outlier distribution?

Response 1.4: Thank you for your question. Based on over three years of expert-labeled outlier data [2], we have found no significant correlation between catchment population size and outlier frequency (Pearson's r = -0.22, p-value: 0.16). To further demonstrate the relationship between catchment population size and outlier intensity, we have added **new Supplementary Fig. 4** (also shown below), suggesting that for the monitored catchments, population size is not a strong predictor of the outlier distribution. In principle, there could be other relevant factors influencing the frequency and intensity of outliers; however, such relationships are generally difficult to assess from observational data due to the sparsity of outliers. We therefore chose a fixed but broad outlier distribution that provides sufficient flexibility to accommodate outliers across multiple scales. Indeed, the outliers in our dataset (ranging from concentrations corresponding to less than ten to several thousand infections) generally fall between the 98.9% and 99.9% quantile of this distribution. We argue that a phenomenological outlier model as used here specifically works well because we otherwise use accurate, mechanistic components to represent the measurement noise from PCR quantification.

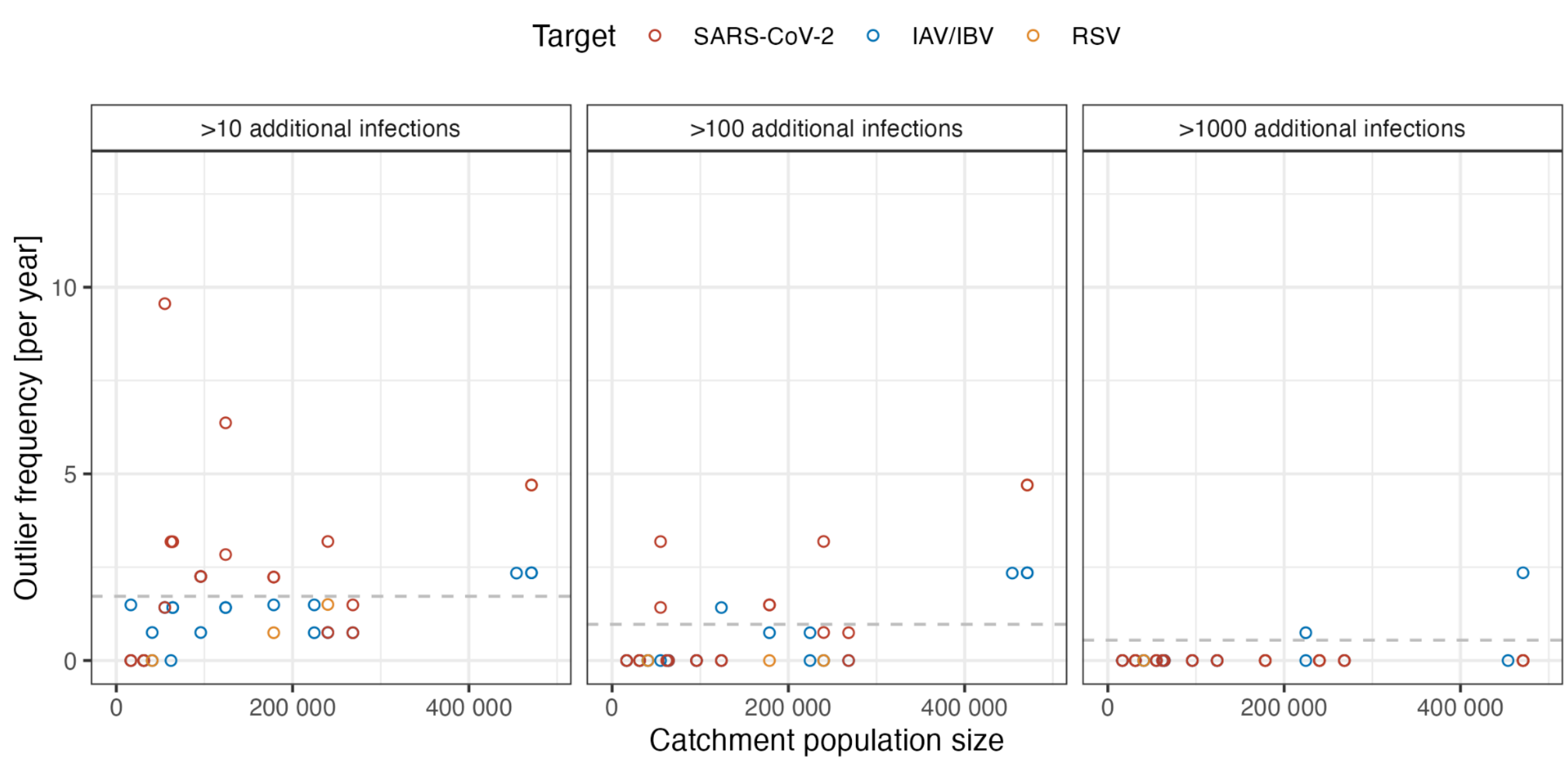

Supplementary Fig. 4. Frequency and size of observed outliers. Shown are observed yearly frequencies of outliers across pathogen targets and locations by size of the catchment population. Outliers were labeled by human experts and grouped by size, corresponding to the number of additional infections implied. Additional infections were estimated using the calibrated detectable load per infection and the deviation of the outlier concentration from a robust, retrospective trend estimate [2]. Dashed horizontal lines show the expected outlier frequency implied by our extreme value distribution prior.

Based on your comment, **we clarified our rationale for using a fixed outlier distribution** (Supplementary Information C.4):

"While there are various potential explanations for such spikes, including unrepresentative sampling and sewer-level effects, evidence on the exact mechanisms leading to outlier observations is still limited. Moreover, the sparsity of outliers renders

identification of predictors for outlier frequency and intensity highly challenging. We thus employ a phenomenological approach in which we parameterize ε_t using a fixed but broad outlier distribution that accommodates outliers across multiple scales.”

References

[2] McLeod RE. Outlier Detection in Digital PCR Wastewater Time Series Data; 2024. Master thesis, ETH Zurich. https://doi.org/10.3929/ethz-b-000702298. Accessed 3 February 2026.

Comment 1.5: - Is there a rationale for why the outlier term is only positive and additive? When looking at concentration signals, it seems that low-value data points far from the main trend are prevalent as well, unless those are correctly captured by the base model. Is there a particular reason also for preferring an additive model for outliers instead of a multiplicative one?

Response 1.5: These are great questions that touch upon some important considerations for modeling outliers in wastewater data. First, several mechanisms that have been suggested as possible explanations for extreme concentration measurements from wastewater likely have asymmetric effects on measured concentrations. For example, settling-resuspension phenomena may lead to highly non-linear changes in sample concentrations (i.e. gradual settling, rapid resuspension caused by an external event), potentially favoring positive outliers [3]. While evidence on these mechanisms is still limited, it is prudent to treat positive and negative outliers as potentially separate phenomena. Second, we indeed find that observed lower concentrations are already well captured by our base model, which covers two major factors leading to large negative deviations from the main trend: i) dilution of genetic material at high flow rates (e.g. due to extreme rainfall) and ii) large absolute measurement error in digital PCR at high concentrations (due to a near-constant coefficient of variation at high sample concentrations). By accounting for these factors, our model achieved well-calibrated coverage of observations across pathogens. We thus believe that additional modeling of negative outliers is not required. Finally, we have proposed an additive rather than a multiplicative component for modeling outliers due to reasons of identifiability. As sample concentrations in digital PCR approach zero, the coefficient of variation of measurements grows without bound. At low concentrations, this increased relative measurement error becomes practically indistinguishable from a multiplicative outlier component. For example, a 100% deviation from the expected concentration would qualify as an outlier at high concentrations, but is consistent with typical dPCR measurement error at very low concentrations. In contrast, by modeling outliers as additive deviations from the expected concentration, we can apply a consistent prior across epidemic phases that matches the calibrated load per infection (i.e. by choosing parameters of the extreme

value distribution such that 99% of the probability mass is below a concentration corresponding to one infection in the catchment). Thus, while current theories about the causes of outlier measurements do not clearly favor an additive or multiplicative mechanism, we have found additive modeling to be a practical approach that gives sufficient flexibility to the model as to remain robust against extreme concentration measurements. We agree that these detailed considerations are valuable to discuss and have integrated them in the corresponding section in **Supplementary Information C.4** for the interested reader.

References

[3] Wade MJ, Lo Jacomo A, Armenise E, Brown MR, Bunce JT, Cameron GJ, et al. Understanding and Managing Uncertainty and Variability for Wastewater Monitoring beyond the Pandemic: Lessons Learned from the United Kingdom National COVID-19 Surveillance Programmes. Journal of Hazardous Materials. 2022;424:127456.

Comment 1.6: - Table S1: I'm assuming Normal+ refers to a positive truncated normal distribution? Please specify.

Response 1.6: Exactly. We used Normal^{+} to refer to the positive truncated normal distribution. We have added this clarification to the relevant places in the main text and Supplementary Information.

Reviewer #2

Comment 2.0: In this work, the authors developed a framework Episewer to infer the pathogen transmission dynamics, specifically the effective reproduction number from wastewater testing data. The authors showed this Bayesian based framework can successfully estimate the real time Rt based on measured viral concentrations in wastewater with quantified uncertainty for SARS-CoV-2, influenza A, and RSV. They tested the framework using data from 3 wastewater treatment plants during the 2023-2024. My major comments are in the method section, some of which are incorrect based on outdated data, and need extra details to justify. For the inferred Rt, the work also lack evidence to validate the effectiveness of the approach.

Response 2.0: Thank you for your constructive feedback on our methodology. In our response below, we carefully address your comments related to our shedding load model and additional factors influencing gene concentrations. We also address your concerns regarding the evaluation of our approach.

Comment 2.1: Specific comments:

1. Viral shedding load modeling issues: One important player in the wastewater data is the viral shedding load. However, the approach for shedding load modeling is problematic. For example, for IAV, the authors used a gamma distribution with normal priors, μ^load and variation νζ based on literature Nadeau et al., 1998. Several issues: First, the paper Nadeau et al., 1998 measured viral titers, not the viral genome copies, which is typically orders of magnitude larger than viral titers, and this will certainly make the shedding load per person significantly lower, and thus impact the estimation of infectious population size and estimated effective reproduction number.

Response 2.1: Thank you for giving us the opportunity to clarify. In our manuscript, we have used the term "shedding load distribution" to denote τ^{shed}, the distribution of the total load shed by an individual over time. For example, when using a discretized gamma distribution for τ^{shed}, this can be interpreted as giving the *percentage* of the total load shed on day 0, 1, 2, … after infection. Importantly, this distribution does not carry any information on the total detectable shedding load per infected individual μ^{load} (note that the mean of the shedding load distribution is parameterized by μ^{shed}, not by μ^{load}). Accordingly, we used literature on fecal and oral shedding of pathogens (e.g. Nadeau et al. 2024 and Carrat et al. 2008 for IAV) **only to inform the relative distribution of shedding loads over time since infection**, not to define the total detectable shedding load per infected individual μ^{load}. The latter is calibrated independently for each wastewater treatment plant, as it not only depends on individual shedding but also sewage and sampling-related factors (see Methods, line 440 ff.). Moreover, in

Supplementary Information E.3, we show the robustness of R_t to miscalibration of μ^{load}, which primarily serves as a scaling factor to the incidence time series without affecting trends.

We recognise that the terminology used to describe shedding load models varies across the current literature and is potentially ambiguous. In response to your comment, **we therefore renamed τ^{shed} from "shedding load distribution" to "(temporal) shedding profile"** for better distinction from the absolute shedding load per individual μ^{load}.

Comment 2.2: Second, in equation 3 (line 345), the μ is the average detectable total shedding load per person, but the literature data measured infectious viral concentration, so one missing component is the material load (here can be feces or materials contributed to the sewer system per person per day), and that varies in the population, and the authors need to mention how they incorporate that in to the model.

Response 2.2: This is a great point. As discussed in Response 2.1, we use literature on shedding only to parameterize the relative distribution of the load over time (since infection). The average detectable total shedding load per infected individual (μ^{load}) is not based on literature; it is instead calibrated. Nonetheless, we agree that variation in material load is a potential factor to consider, since our parameterization is based on the assumption that the material load is constant over the course of an infection. For respiratory viruses such as SARS-CoV-2, IAV, and RSV, we regard this as a reasonable assumption at the population level. Under constant material shedding, the distribution over time (i.e. profile) of the load is identical to the distribution of the measured concentration in the sampled material (the material load only serves as a scaling factor that does not affect the relative shedding profile). Moreover, variation in the absolute material load between individuals is captured by our modeling of individual-level variation in μ^{load}. However, if the material load varies over the course of an infection, which is particularly plausible for gastrointestinal pathogens, the distribution of measured viral concentrations over time may not accurately reflect the actual load profile. In such a setting, we agree that changes in the material load throughout an infection would have to be incorporated when defining priors for the shedding profile. To reflect these considerations, **we now discuss the role of material load for parameterizing the shedding profile** in the Methods (line 432 ff.):

"For IAV and RSV, we used the relative distribution of viral concentration measurements from nasal wash or swab samples as a proxy for shedding into wastewater over time, assuming constant material loads over the course of infection. Note that for other pathogens, specifically gastrointestinal viruses, time-varying material loads might need to be accounted for."

We also provide a more detailed description in **Supplementary Information C.2**:

“Moreover, our shedding profiles for IAV and RSV reflect the temporal distribution of viral concentrations measured from patient samples. This is based on the assumption that the overall material load shed is constant over the course of an infection and thus only serves as a scaling factor that does not affect the relative shedding profile. For other pathogens, such as gastrointestinal viruses with variation in the amount of fecal excretion over the course of an infection, material loads might need to be incorporated when estimating the distribution of loads over time.”

Comment 2.3: Third, the data are based on nasal swab samples, it may be fine to make assumptions to connect nasal viral load vs stool viral load, but a reasonable justification is needed.

Response 2.3: Thank you for addressing this aspect. In principle, shedding of genetic material into the wastewater could occur through other activities than toilet use, including handwashing, showering, or tooth brushing. This implies various potential shedding sources (urine, feces, nasal secretions, saliva, etc.), and there is currently a lack of evidence on what sources are dominant for most pathogens. Specifically, for IAV and RSV, we did not find conclusive evidence on whether shedding into wastewater is primarily oral or fecal in origin. Our decision to use viral load measurements from nasal samples for these pathogens was therefore mostly based on the availability of quantitative estimates from these studies. We agree that the limited clinical evidence on wastewater-relevant shedding for most pathogens is an important challenge in interpreting wastewater data. In response to your comment, **we have explicitly highlighted the uncertainty around different sources of shedding** as a limitation in the Discussion (line 297 ff.):

“[...], we emphasize that clinical evidence on wastewater-related shedding remains limited. This includes uncertainty regarding the predominant sources of shedding into wastewater, e.g. urine, feces, nasal secretions, saliva, etc., for most pathogens. For IAV and RSV, we used available evidence from nasal samples to parameterize the shedding profile, noting that the relative contributions of fecal and oral shedding into wastewater remain largely unclear.”

Comment 2.4: Fourth, the viral shedding can be very different from different variants or strains. Like for influenza, the H1N1 usually have higher shedding in stool, but other influenza types are barely detectable in feces. This applies to SARS-CoV-2, like Omicron's shedding seems much larger than the wild type. The authors need to consider this for a temporal estimation of Rt, especially for these fast evolving virus.

Similar issues apply to the SARS-CoV-2 and RSV shedding load modeling.

Response 2.4: We agree that this is an important point. We have shown in earlier work that R_t can be temporarily biased when a dominant variant is replaced by another variant with substantially different shedding intensity [4]. Importantly, however, this bias is only transient, i.e. R_t will be biased for a brief transition phase until the new variant has become dominant in the population. Beyond such transitions, when variant proportions are roughly stable, transmission dynamic estimates remain unbiased due to the scale invariance of the R_t estimator. For the monitored time period (winter seasons 2022/23 to 2024/25) in Switzerland, circulation of SARS-CoV-2 has been consistently dominated by the Omicron variant, although some sublineages may exhibit increased fecal shedding [5]. For influenza A virus, both pandemic (H1N1pdm09) and seasonal (H3N2) lineages have been present in Switzerland over the monitored time period, however we did not find clear evidence for pronounced differences in fecal [6] or oral [7] shedding between H1N1pdm09 and H3N2. For RSV, we found no evidence for or against differences in shedding of RSV-A and RSV-B due to a general lack of quantitative shedding studies stratified by subtype.

The risk of temporary bias during variant transitions is an inherent limitation of any method used to estimate R_t at the population level. Adjusting R_t estimates during such a transition would require accurate estimates of the proportions of individuals infected with each variant over time and of their relative differences in shedding, which depend on case-based testing and clinical studies, and are typically not available at sufficient resolution and speed. However, as a more practical alternative, wastewater-based surveillance may also be used to estimate variant-specific R_t estimates, which are fairly robust to miscalibration of the assumed average load per infection (as shown in Supplementary Information E.3). These can be obtained using clade-specific PCR assays, which target one or several signature mutations of a variant to measure its absolute concentration in wastewater [8,9]. Given such concentration measurements, our method can be readily applied to produce variant-specific R_t estimates. An alternative approach would be to combine population-level concentration measurements with wastewater sequencing data to jointly model the total absolute and variant-specific relative loads in wastewater. A Bayesian generative modeling approach as used in this work seems well-suited for this purpose, and we plan to extend our wastewater model to integrate sequencing data in future work. In response to your comment, we have revised our manuscript as follows:

1. We **clarified the risk of differential shedding of variants**, pointing to previous work that studied its temporary impact on R_t estimates (lines 272-273, 280 ff.):

“[...], although our model is robust to miscalibration of the average shedding load per infection, it does not account for potential differences in shedding between variants of the same pathogen. [...] Generally, when a variant with substantially different shedding intensity replaces the dominant variant in a population, R_t estimates will be biased for a brief transition period. Importantly, however, this bias is only temporary and vanishes when the new variant has become dominant.“

2. We **discussed the potential for differential shedding in the pathogens monitored by our study** (line 273 ff.):

“For the time period monitored in this study, SARS-CoV-2 infections in Switzerland were consistently dominated by the Omicron variant; however, Wannigama et al. found evidence for increased fecal shedding in the later sublineages BA.2.86 and JN.1. For influenza A virus, both infections with pandemic (H1N1pdm09) and seasonal (H3N2) strains were common during the monitored time period, but we found no clear evidence for marked differences in shedding between these strains. For RSV, there is an overall lack of quantitative evidence on shedding stratified by RSV-A and RSV-B subtypes.”

3. We **outlined the potential of variant-specific R_t estimation** using specialised PCR assays or by incorporating wastewater sequencing data (line 283 ff.):

“This inherent limitation of population-level R_t estimation from wastewater may be overcome through variant-specific R_t estimates, which can be obtained by applying EpiSewer to concentration measurements from variant-specific PCR assays. Alternatively, our model could be extended by integrating wastewater sequencing data to jointly model the total absolute and variant-specific relative loads of a pathogen.”

References

[4] Dreifuss D, Huisman JS, Rusch JC, Caduff L, Ganesanandamoorthy P, Devaux AJ, et al. Estimated Transmission Dynamics of SARS-CoV-2 Variants from Wastewater Are Unbiased and Robust to Differential Shedding. Nature Communications. 2025;16(1):7456.

[5] Wannigama DL, Amarasiri M, Phattharapornjaroen P, Hurst C, Modchang C, Chadsuthi S, et al. Increased Faecal Shedding in SARS-CoV-2 Variants BA.2.86 and JN.1. The Lancet Infectious Diseases. 2024;0(0).

[6] Minodier L, Masse S, Capai L, Blanchon T, Ceccaldi PE, van der Werf S, et al. Risk Factors for Seasonal Influenza Virus Detection in Stools of Patients Consulting in General Practice for Acute Respiratory Infections in France, 2014-2016. Influenza and Other Respiratory Viruses. 2019;13(4):398–406.

[7] Suess T, Remschmidt C, Schink SB, Schweiger B, Heider A, Milde J, et al. Comparison of Shedding Characteristics of Seasonal Influenza Virus (Sub)Types and Influenza A(H1N1)Pdm09; Germany, 2007–2011. PLOS ONE. 2012;7(12):e51653.

[8] Caduff L, Dreifuss D, Schindler T, Devaux AJ, Ganesanandamoorthy P, Kull A, et al. Inferring Transmission Fitness Advantage of SARS-CoV-2 Variants of Concern from Wastewater Samples Using Digital PCR, Switzerland, December 2020 through March 2021. Eurosurveillance. 2022;27(10):2100806.
[9] Pitton M, Gan C, Schmidhalter P, Ort C, Julian T. Clade-Specific Multiplex Digital PCR Assay for Monkeypox Virus Detection in Wastewater. Access Microbiology. 2025; p. 001041–v1.

Comment 2.5: missing environmental and demographic variation factors. The authors nicely considered the potential variation to the detection results in wastewater. but i think two important ones were missed. First, transit and viral genetic loss in the sewer. For example, in equation 2 (line 332), the expected viral concentration equals the total load divided by the flow. Other factors like retention time, viral degradation, viral loss from biofilms, viral stability…etc. those factors all impact the expected concentration, and can be quite large for different wwtps, and also sewer specific especially for some old ones.

Response 2.5: Thank you for highlighting these factors. We agree that both environmental and demographic variation can in principle affect the estimation of transmission dynamics. There are indeed a multitude of environmental and sewer-specific factors that can influence the concentration measured in daily composite samples. However, for the estimation of transmission dynamics, it matters most whether these sewer conditions are approximately constant over time or time-varying. This is because our model uses the total detectable load per infected individual (μ^{load}) to summarize all time-constant factors influencing detectable loads. That is, μ^{load} does not merely represent the amounts of genetic material shed by infected hosts, but also the overall loss during in-sewer transport, sampling, and sample processing. We calibrate μ^{load} separately for each location to account for effects specific to the sewage system and sampling at the wastewater treatment plant (see Methods, line 440 ff.). Moreover, in Supplementary Information E.3, we demonstrated that R_t estimates are notably robust to miscalibration of μ^{load}.

Nevertheless, environmental factors can indeed affect R_t estimates when they are time-varying. A clearly time-varying factor is wastewater flow. As we show in Supplementary Information E.1, neglecting time-varying flow increases the uncertainty of R_t estimates due to increased noise and can lead to bias from systematic seasonal differences in flow rates. We have therefore included flow in our model, but find it plausible that there could be further, more nuanced time-varying factors (such as differences in viral degradation due to fluctuations in flow or temperature). Unfortunately, there is still a lack of generalizable estimates specifically on the degree to which such factors vary over time and the exact impact this has on target concentrations. Thus,

further research in controlled sewer environments is needed to inform the modeling of detectable pathogen loads.

In response to your comment, we revised our manuscript as follows:

1. We clarified that due to uncertain environmental and other factors, **our model can only provide reliable estimates of transmission dynamics, not of absolute incidence** (line 266 ff.):

"[...], our approach is designed to estimate relative changes in transmission over time rather than absolute numbers of infections in a catchment. The latter are generally difficult to infer from wastewater data alone, due to considerable uncertainty regarding the total amount of pathogen shedding and the loss of detectable genetic material during in-sewer transport and laboratory processing. Therefore, the latent parameter I_t in our model should only be used for model inspection and should not be interpreted in terms of absolute incidence."

2. **We added a discussion on the role of time-varying environmental factors beyond flow** to the Limitations (line 293 ff.):

"[...], our approach is robust to sewer-specific factors influencing detectable gene loads, such as viral degradation and adsorption onto biofilms, as long as these factors do not vary systematically over time. To reliably model time-varying loss of detectable RNA/DNA in the sewer, more research is needed to quantify the effect of fluctuating environmental variables on these phenomena."

Comment 2.6: Second, the moving population in each sewershed. This variation has not even been considered in the total load shed, but their differences can be very large depends on the location/business of the community, like some places have high outside visitors/travellers.

Response 2.6: We agree with this limitation. To account for demographic variation, detailed data on population movements are required, which are currently not available to us across the monitored time period. To address your concern, **we conducted a sensitivity analysis with simulated variations in population size** between working days and weekend days/holidays (**new Supplementary Information E.5**). This reflects a typical mobility pattern for larger cities in Switzerland, which experience significant commuter traffic from neighboring communities [10]. We find that differences in loads between working days and weekends induced by such commuting behaviour have a negligible effect on R_t estimates (**new Supplementary Fig. 23**), as they are on the scale of the generation time distribution and thus captured as unexplained variation.

Nonetheless, more irregular, longer-term differences in population size could have the potential to distort transmission dynamic estimates if unaccounted for, e.g. in areas with a pronounced tourist season or due to non-pharmaceutical interventions during public health emergencies [11]. More detailed modeling of population movements will necessitate a better understanding of mobility types and their relationship with wastewater-relevant pathogen shedding.

In response to your comment, **we expanded our discussion to better highlight the risk of bias from irregular mobility changes** (line 314 ff.):

"However, the population contributing to transmission may differ from the wastewater catchment population, and the latter may vary due to human mobility, with potential impacts on the total load shed in the catchment over time. While sensitivity analysis suggests that our approach is robust to regular weekly fluctuations in the population size, such as those caused by commuting (Supplementary Information E.5), more irregular changes in the catchment population, e.g. due to non-pharmaceutical interventions or tourism, could bias estimates of transmission dynamics if unaccounted for."

The details and assumptions of our sensitivity analysis are described in **Supplementary Information E.5**. There we also point out uncertainties regarding the relationship between mobility and wastewater loads:

"[..] we remark that the effective load shed in the catchment could also be more stable than the population size, since shedding may be predominantly residential (e.g. as individuals may shed primarily outside of working hours or stay at home when symptomatic)."

References

[10] Statistik Stadt Zürich. Pendler*innen nach Wohnort und Arbeitsort, 2023. Stadt Zürich [Website]; 2023. Available from https://www.stadt-zuerich.ch/de/politik-und-verwaltung/statistik-und-daten/daten/mobilitaet/pendelnde.html#publikationen.

[11] Pullano G, Valdano E, Scarpa N, Rubrichi S, Colizza V. Evaluating the Effect of Demographic Factors, Socioeconomic Factors, and Risk Aversion on Mobility during the COVID-19 Epidemic in France under Lockdown: A Population-Based Study. The Lancet Digital Health. 2020;2(12):638–649.

Comment 2.7: 3. In Line 348, 'I' is described as the number of infected individuals, weighted by overall shedding intensity. But if it is 'infected individuals', then in equation 4 (line 359), it would be incorrect to use the total infected individuals multiplied by the

instantaneous effective reproduction number. From equation 5, if I follows a Poisson distribution, I guess it shall be ‘incidence’, not prevalence (infected individuals). This needs clarification.

Response 2.7: Thank you for pointing this out. You are perfectly right that I_t denotes the number of new infections at time t rather than the total number of infected individuals. **We ensured that I_t is correctly described as the number of new infections** throughout the manuscript.

Comment 2.8: 4. Line 380 assumed 8-17.7 days for the shedding duration for SARS-CoV-2, but that reference is for data in August to November 2020, during the wild type period. But the measured data actually covers 2023 to 2024, during the Omicron period. The authors need to update based on variant data.

Response 2.8: Indeed, the study used to inform our shedding profile prior for SARS-CoV-2 is based on measurements from the wild type period. Unfortunately, we were not able to find exact quantitative estimates for the temporal shedding profile of the Omicron variant; however, clinical evidence on the fecal shedding of several Omicron sub-variants suggests a peak in shedding around 7-9 days after symptom onset, with the majority of patients shedding for at least 21 days after symptom onset [12]. Accounting for an average incubation period of 3-4 days for the Omicron variant [13], this time window is compatible with our broad prior for the shedding profile (mean of 8.0-17.7 days, 90% quantile of 17-29 days). Based on your comment, **we clarified that the temporal shedding profile was parameterized using estimates based on the wild type of SARS-CoV-2**, and that updated evidence on the shedding of the Omicron variant is **compatible with this prior** (line 427 ff.):

“For SARS-CoV-2, we used a gamma distribution with a mean of 8.0--17.7 days, based on an observational study that combined wastewater measurements with case surveillance data collected between August and December 2020. Although this study characterized shedding for the SARS-CoV-2 wild type, its estimates are consistent with later clinical evidence on shedding kinetics for several Omicron subvariants.”

References

[12] Wannigama DL, Amarasiri M, Phattharapornjaroen P, Hurst C, Modchang C, Chadsuthi S, et al. Increased Faecal Shedding in SARS-CoV-2 Variants BA.2.86 and JN.1. The Lancet Infectious Diseases. 2024;0(0).

[13] Xu X, Wu Y, Kummer AG, Zhao Y, Hu Z, Wang Y, et al. Assessing Changes in Incubation Period, Serial Interval, and Generation Time of SARS-CoV-2 Variants of Concern: A Systematic Review and Meta-Analysis. BMC Medicine. 2023;21:374.

Comment 2.9: Also, in lines 375-385, those descriptions for SARS-CoV-2, RSV, Influenza are not 'shedding load distribution' as described in the paper, it more seems 'shedding duration'.

Response 2.9: We have revised the manuscript and **now use the term "(temporal) shedding profile"** instead, which is less ambiguous and has been previously used in the literature to describe the time distribution of the shedding load [14].

References

[14] Hoffmann T, Alsing J. Faecal Shedding Models for SARS-CoV-2 RNA among Hospitalised Patients and Implications for Wastewater-Based Epidemiology. Journal of the Royal Statistical Society Series C: Applied Statistics. 2023;72(2):330–345.

Comment 2.10: 5. line 47-48, the statement is incorrect. The two literatures cited did not talk about the 'inherent delays between infection and shedding'. Both talked about the delay between infection and testing.

Response 2.10: The two cited studies show that SARS-CoV-2 fecal RNA shedding occurs over a window relative to infection (and symptom onset), rather than being instantaneous. Cavany et al. (2022) explicitly infer the shedding-time distribution relative to the time of infection, and Arts et al. (2023) quantify fecal shedding over time relative to symptom onset using fecal stool samples. They both support our claim that wastewater signals can lag behind transmission dynamics in the community, as shedding is distributed over a longer period of time and typically peaks only several days after infection. To avoid potential confusion with other types of delay, such as from testing, **we revised the sentence** to be more explicit (line 47 f.):

"Because fecal shedding is distributed over time after infection, wastewater concentrations tend to lag transmission dynamics in the community."

Comment 2.11: 6. There are a dozen papers that reported the Rt estimation from wastewater data as well as R packages developed, as the authors mentioned some in the paper. There are some more like PMID: 39566444, PMID: 39729960. It would be greatly helpful to have a benchmark and the unique strengths for this work shall be discussed.

Response 2.11: Thank you for pointing out these additional studies. **We have included them as references** in the Introduction (line 22 f., line 48 ff.). Moreover, we agree that benchmarking against other approaches would, in principle, be very helpful. However, in the absence of a ground truth for the true reproduction number, conducting an

informative and fair benchmark is non-trivial, and we see two main avenues, both with current limitations.

First, as illustrated in this paper, short-term forecasts of flow-normalized wastewater concentrations can be used to assess whether the model accurately predicts the trend of the total load shed in a catchment. This task is closely linked to estimating the real-time growth trend in infections (and, for a given generation time distribution, to estimating R_t). A strength of this approach is that it is fully data-driven without relying on clinical case reporting. However, a current limitation for broader benchmarking is that most existing approaches for wastewater-based R_t estimation (e.g. deconvolution-based methods) are not fully generative and therefore do not naturally provide concentration forecasts in a meaningful way. Some models instead forecast case numbers, which is a valuable application, but this introduces an additional data-generating process and inherits biases from case reporting, making such forecasts less suitable for assessing performance in estimating R_t. A wider forecast-based benchmark would therefore require the development of more models that explicitly generate probabilistic forecasts of wastewater concentrations.

Second, one could perform a simulation-based benchmarking study with simulated ground truth R_t. While straightforward simulations can be constructed, they inevitably favor the modeler's assumptions. A credible benchmark would likely require an independent, detailed mechanistic model of both transmission and wastewater processes, ideally developed collaboratively by researchers with diverse modeling perspectives. Since this represents a substantial project in its own right, we hope you understand that this is beyond the scope of the present study.

In response to your comment, **we added a discussion about the benchmarking of different models** using forecast- and simulation-based validation (line 248 ff.).

"[...], forecast-based metrics could be used to benchmark alternative wastewater models, however many existing methods rely on non-parametric smoothing or interpolation of the data and do not naturally yield probabilistic forecasts of measured concentrations. Simulations could also be used to assess real-time transmission dynamic estimates across models, but a fair simulation-based benchmark would require an independent mechanistic model of transmission and wastewater processes, which is beyond the scope of this study."

Reviewer #3

Comment 3.0: This is an interesting and elegant model. My primary concern is with the calibration of the shedding per individual. The authors have calibrated this to case incidence data, which raises a question about how these case data were interpreted and why they weren't included explicitly in the time series analysis.

Response 3.0: Thank you for your appreciation of our work. We address your concerns regarding the use of case incidence data in detail below.

Comment 3.1: Figure S16 indicates that Rt is relatively robust to assumptions about shedding, but the absolute incidence is certainly not. As expected, the inferred incidence (which many would consider a more important quantity than Rt to get right in absolute value) is negatively correlated with the assumed shedding. The estimated incidence in Figure S16 varies over orders of magnitude.

Response 3.1: We agree. Our shedding model implies that the estimated expected number of new infections $E[\mathbf{I}_t]$ scales proportionally with the expected total shedding load per infected individual and is therefore directly dependent on this assumption. In contrast, indicators of transmission dynamics such as R_t are essentially invariant to the assumed load per infected individual - except when the absolute incidence becomes so low that transmission is considerably influenced by stochastic events, leading to increased uncertainty in R_t. Thus, the main implication of our sensitivity analysis (former Supplementary Fig. 16, now 18) is that while $\mathbf{I}_t$ does not represent a reliable estimate of the absolute incidence, R_t (and growth rate) estimates remain robust even if the assumed shedding load per infected individual is misspecified by orders of magnitude. We think that this is an important finding, since R_t and growth rates track the timing and transmission intensity of seasonal waves of circulating pathogens, and have served as important indicators to guide public health policy during outbreaks [15, 16].

We apologize if this has not been sufficiently clear in the manuscript. **We have clarified that case data used for calibration of the total shedding load per infected individual are generally not unbiased** and that any bias in μ^{load} will affect estimates of I_t, while R_t remains largely robust (line 443 ff.):

"In our main analysis, we calibrated μ^{load} to catchment-specific infection numbers estimated using publicly available data from Sentinella reporting and participatory surveillance in Switzerland (Supplementary Information C.3). Generally, case data used for calibration cannot be assumed unbiased due to imperfect ascertainment and reporting, and any bias in the calibration of μ^{load} will affect the estimated number of new infections I_t in our model. However, as μ^{load} scales I_t only by a constant factor,

transmission dynamic estimates remain invariant to such miscalibration except when incidence is low enough for stochastic infection and shedding dynamics to dominate (Supplementary Fig. 18)."

Moreover, we highlighted our **sensitivity analysis of the calibration approach**, in which we calibrated μ^{load} without using any case data and obtained practically identical R_t estimates (line 450 ff.):

"Accordingly, we obtained practically identical R_t estimates for most catchments using a simpler calibration without case data, in which the initial load detected at the start of each season was equated to an incidence of 0.01% of the catchment population (Supplementary Figs. 15-17)."

References

[15] Gallien Y, Paireau J, Paty AC, Villegas-Ramirez B, Hamidouche M, Modenesi G, et al. Using the near Real-Time Effective Reproduction Number Rt as an Early-Warning Tool for Seasonal Bronchiolitis and Influenza-like Illness Epidemics. American Journal of Epidemiology. 2024;194(5):1332–1340.

[16] Sharma M, Mindermann S, Rogers-Smith C, Leech G, Snodin B, Ahuja J, Sandbrink JB, Monrad JT, Altman G, Dhaliwal G, Finnveden L. Understanding the effectiveness of government interventions against the resurgence of COVID-19 in Europe. Nature communications. 2021 Oct 5;12(1):5820.

Comment 3.2: The authors are heavily focussed on the robustness of Rt, which is impressive. But the indeterminate estimates of incidence require more attention here. If the answer is that incidence cannot be reliably estimated without very explicit quantification of individual-level shedding, then that needs to be better explained. If there is bias (e.g. due to under-reporting) in the reported incidence data that the authors use to calibrate their shedding rate per individual, then Figure S16 would indicate that the estimates of absolute incidence cannot be trusted. If the reported incidence values used ARE unbiased, then they could be used to constrain the model. An even better thing to do would be to estimate total incidence and Rt from the reported incidence time series alone and compare those estimates to the waste water-based estimates. The authors indicate in the text (starting in L225) that they don't favor that approach to calibration. Instead they validate based on forecasts of wastewater concentration and Rt; the former works impressively, but forecasts a quantity that isn't operationally useful without translation into cases, or similar.

Response 3.2: Thank you for stressing this important aspect. As you have perfectly pointed out, estimates of I_t under our model are dependent on the calibrated shedding load per infected individual, and we generally do not regard clinical data used for

calibration as unbiased (specifically due to a high risk of underreporting with these data, as you have highlighted). Consequently, our estimates of I_t should not be interpreted as accurate estimates of the true absolute incidence. It is exactly for this reason that we have been using I_t **only for internal model assessment**, but did not report I_t in our main results to avoid misinterpretation. Equivalently, our real-time dashboard shows only loads and R_t, but not incidence estimates (https://wise.ethz.ch), and our R package explicitly discourages interpreting I_t as absolute incidence (see README).

In response to your comment, **we clarified that estimates of I_t should not be interpreted as estimates of absolute incidence** and may only be used for model inspection (line 266 ff.):

"[...], our approach is designed to estimate relative changes in transmission over time rather than absolute numbers of infections in a catchment. The latter are generally difficult to infer from wastewater data alone, due to considerable uncertainty regarding the total amount of pathogen shedding and the loss of detectable genetic material during in-sewer transport and laboratory processing. Therefore, the latent parameter I_t in our model should only be used for model inspection and should not be interpreted in terms of absolute incidence."

Moreover, we better **explained why we prefer calibration of μ^{load} over jointly fitting our model to wastewater and case data** (line 453 f.). The key point is that case counts are often biased through time (e.g. due to changes in testing intensity), and this bias will affect R_t estimates when fitting to such data [17]. In contrast, R_t estimates from wastewater remain robust even when temporally biased case data are used for calibration of μ^{load}. T

To illustrate this, **we added a simulation analysis (new Supplementary Fig. 19, also shown below)** in which we applied a simulated change in the reporting proportion over time to the case counts used for calibration of μ^{load}, showing that the resulting R_t estimates remain practically unchanged.

References

[17] Gostic KM, McGough L, Baskerville EB, Abbott S, Joshi K, Tedijanto C, et al. Practical Considerations for Measuring the Effective Reproductive Number, Rt. PLOS Computational Biology. 2020;16(12):e1008409.

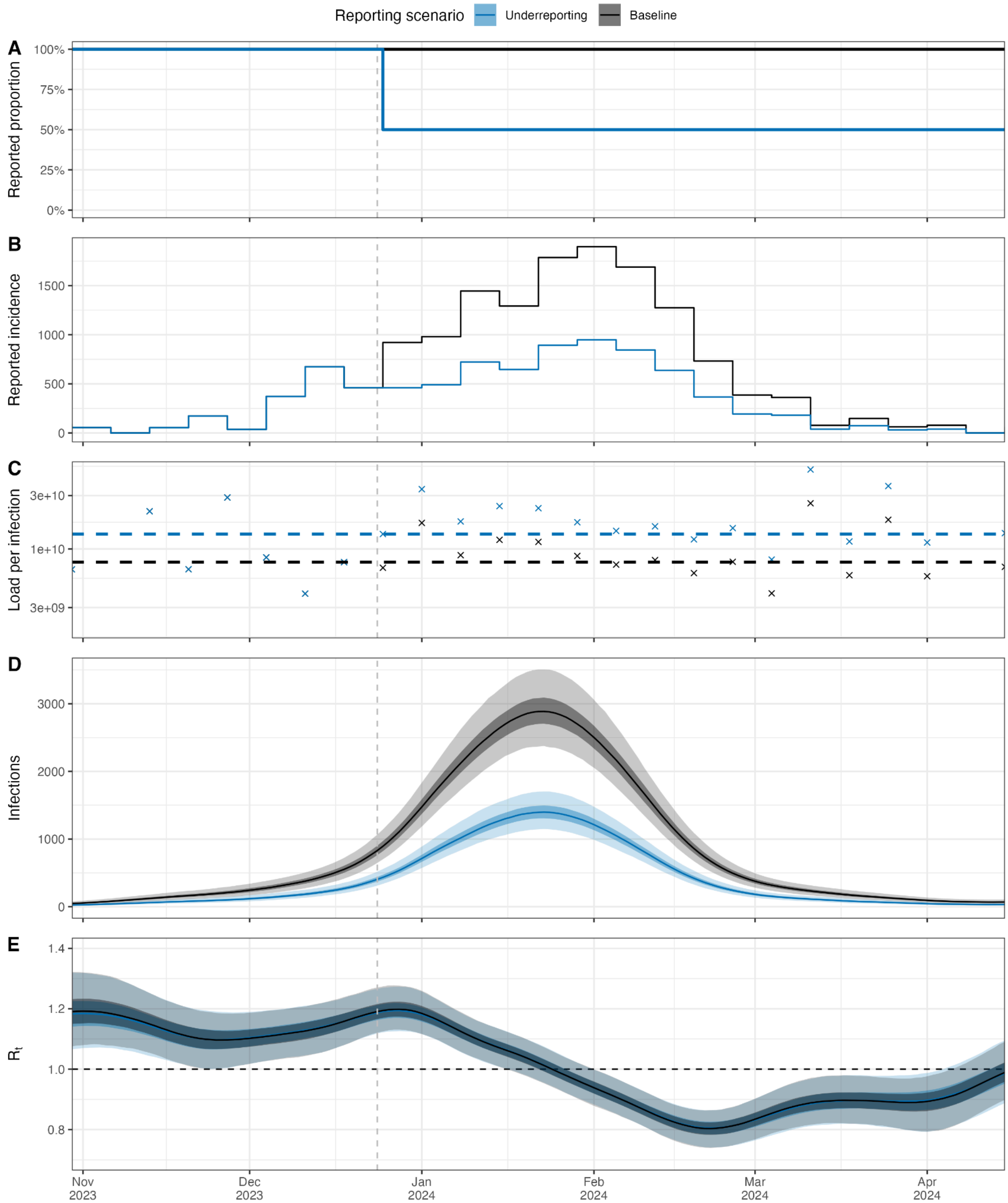

Supplementary Fig. 19. Robustness of estimated transmission dynamics to calibration from case data with time-varying underreporting. (A) We simulated a 50% reduction in the reporting of Influenza A virus infections in Zurich from December 24, 2023 onward (vertical dashed line). (B) This results in a time-varying bias of the reported incidence that would substantially distort case-based R_t estimates. (C) Underreported incidence impacts the calibration of the average total detectable load per infection in our model (horizontal dashed lines). (D) This miscalibration also biases the number of new infections as estimated from wastewater, but only by a constant scaling factor. (E) R_t estimates remain robust to the miscalibration.

Comment 3.3: I would like to see the authors do some validation either to observed time series of cases (illustrating whether incidence is estimable in if shedding is mis-specified) or to simulated data where all values are known. The latter would be tractable if the authors don't feel confident in the application of standard methods to the likely underreported time series are valid. Finally, if this methods works for Rt, but not for incidence, then that needs to be more clearly disclosed in the limitations of the study.

Response 3.3: Indeed, this method uses I_t as a latent internal parameter to infer transmission dynamics, but it does not aim to recover absolute incidence. Our approach is designed to infer relative changes in transmission over time, which depend on the temporal dynamics of the latent infection process rather than its absolute scale. Thereby, our model enables estimation of R_t or epidemic growth rates - which have been widely used to inform public health situational awareness and response - solely from wastewater concentrations while avoiding well-known biases of case data.

In contrast, we do not claim that absolute incidence is identifiable from wastewater data alone, and we did not conduct a simulation or validation analysis targeting absolute incidence. The inability to reliably infer absolute incidence or prevalence from wastewater measurements remains a general limitation of wastewater-based epidemiology [18-20], and we agree that this should be stated more clearly. We thus **expanded the relevant explanations in the Methods and added an explicit statement to the Limitations** (see action items in Responses 3.1 and 3.2).

References

[18] Daughton CG. Wastewater surveillance for population-wide Covid-19: The present and future. Science of the Total Environment. 2020 Sep 20;736:139631.

[19] Wade MJ, Lo Jacomo A, Armenise E, Brown MR, Bunce JT, Cameron GJ, et al. Understanding and Managing Uncertainty and Variability for Wastewater Monitoring beyond the Pandemic: Lessons Learned from the United Kingdom National COVID-19 Surveillance Programmes. Journal of Hazardous Materials. 2022;424:127456.

[20] Huisman JS, Scire J, Caduff L, Fernandez CX, Ganesanandamoorthy P, Kull A, et al. Wastewater-Based Estimation of the Effective Reproductive Number of SARS-CoV-2. Environmental Health Perspectives. 2022;130(5):057011.

Point-by-point response to reviewers

Reviewer #1

Comment 1.0: The authors have addressed all my comments, providing further details in the supporting information document about parameter estimation, outlier modeling, and other minor remarks. I recommend publication without delay.

Response 1.0: We are delighted to hear that all your comments have been sufficiently addressed.

Reviewer #2

Comment 2.0: I am thankful for the authors to address most of my comments. While many are just acknowledging the limitations in the discussion, but I understand those are hard questions in the field with complications and unknown parameters that we are not very clear so far.

Response 2.0: Thank you, we are glad that our revisions have helped to clarify the limitations of our method and the field in general.

Comment 2.1: One point I don't agree with the authors about the comment 2.10, the authors changed their earlier statement to 'Because fecal shedding is distributed over time after infection, wastewater concentrations tend to lag transmission dynamics in the community [34,35]'. I think this is a very strong statement, and not well studied and supported by the two cited papers. The paper by Arts et al. reported time-varying and heterogeneous fecal viral shedding among individuals, and. The paper by Cavany et al. estimated that individual shedding into wastewater peaks on average about 6 days after infection, with a relatively strong overdisperation. Either of the paper suggests that wastewater concentrations tend to lag community infection dynamics.

moreover, the logical link between time-distributed shedding and a community-level lag is weak. In the early phase of an outbreak, wastewater can reflect increases in transmission well and in later phases, wastewater data may not reflect well, but that is likely driven by the aggregation of viral shedding across infected individuals rather than by a true lag in transmission dynamics (see paper https://doi.org/10.1038/s43856-025-00908-5 for reference). So I think wastewater data reflect community infection dynamics in general, unless the virus infection and excretion

have an obvious lag or the wastewater testing is not sensitive enough to capture those early sheddings, at least not caused by the time distributed shedding.

Response 2.1: We apologize for our potentially imprecise statement and agree that time-distributed shedding by itself does not imply a time lag in wastewater concentrations relative to infection incidence (the presence of lags will depend on the shape of the shedding profile). Rather, as you perfectly describe in your comment, time-distributed shedding means that wastewater concentrations reflect the aggregated, partial shedding loads from individuals infected at different points in the past. This has a blurring effect on the observed concentration time series (see [1] for description of a similar effect in case count data with reporting delay distribution), meaning that – even absent a clear lag – inferences of the underlying infection trajectory and transmission dynamics will be subject to uncertainty near the present. In response to your comment, we have revised our explanation of this phenomenon (line 47 ff.):

"Because fecal shedding is distributed over time after infection, current wastewater concentrations reflect the aggregate, partial shedding of individuals infected at different times in the recent past. Consequently, wastewater data provide a temporally blurred signal of community transmission dynamics, imposing a natural limit on the precision with which R_t or growth rates can be estimated near the present, potentially leading to overconfident real-time estimates if not accounted for."

References

[1] Gostic, K. M. et al. Practical considerations for measuring the effective reproductive number, Rt. *PLOS Computational Biology* 16, e1008409 (2020).

Reviewer #3

Comment 3.0: In general I am satisfied with the authors' response to the reviewers. I still believe some of the language overstates the outcomes.

L 84-97 states "produce Bayesian estimates of ... latent parameters useful for model assessment, e.g. implied infection numbers ...", while it is true these estimate are produced they are not reliable. A more honest statement is that this methods estimates Rt and rt. It also produces estimates and projections of total catchment load. But lumping incidence in to this discussion implies that it is also estimated well, which it is not.

Response 3.0: Thank you for your feedback. We did not intend to suggest that the model-derived infection incidence estimates are accurate on an absolute scale (as

clarified in the Discussion, line 266 ff.). However, we believe that inspecting these estimates can be useful for model-checking purposes by revealing the infection numbers implied by the model inputs and assumptions. We therefore chose to report this output as well. For example, inspecting the estimated infection numbers can help identify gross misspecification of the assumed load per infection or mismatches in the units of the flow and concentration data, which would manifest as implausibly high or low incidence values. To clarify this use case, we revised the sentence by replacing “model assessment” with “model inspection and misspecification checking" and by explicitly noting the dependence on the assumed load per infection (line 98 f.):

“... and other latent parameters useful for model inspection and misspecification checking, e.g. infection numbers (implied by the assumed shedding load per infection) and total pathogen loads.”

Comment 3.1: I would encourage the authors to consider changing the wording "transmission dynamics", which is used as a catch-all here. It seems that phrase could be replaced with "effective reproduction number" without affecting the interpretation or value of the work. Of the estimated parameters in table S3, most are not parameters with a meaningful biological interpretation. Those that are relate to rt (from which Rt is derived), It (which the authors indicate is not well estimated), or shedding, which the appears to be strongly constrained by assumptions. The authors don't appear to be presenting results on what was learned about shedding; rather they are using the hierarchical structure of the model to account for uncertainty in shedding dynamics, but not estimating these dynamics, per se. Thus, from the title onwards, "robust real-time estimation of pathogen effective reproduction number form wastewater" would be a more honest accounting of this work. This is not a criticism of the work or the model, which is impressive. If the editor does not agree with this assessment, I will not pursue it further and will support publication pending the small change to the text mentioned above.

Response 3.1: Thank you for this comment. We have chosen to use the term “transmission dynamics” rather than “effective reproduction number” in our manuscript for several reasons. First, we use “transmission dynamics” to jointly refer to the time-varying effective reproduction number R_t and the epidemic growth rate r_t. Both quantities are jointly and consistently estimated in our framework, which we consider a key strength of the Bayesian generative modeling approach. Referring only to the effective reproduction number in the title would not fully reflect this feature of our model. In contrast, the term “transmission dynamics” encompasses reproduction numbers as well as growth rates, and this use of the term is consistent with the prior literature, see for example [1-6].

Second, the methodological challenges addressed in our work are not specific to the effective reproduction number, but apply more broadly to the inference of transmission-related quantities from wastewater data. For example, uncertainty in the shedding profile, as modeled in this work, affects estimates of the effective reproduction number and epidemic growth rate, but would similarly affect estimates of other transmission-related quantities, such as the force of infection λ in compartmental models or the epidemic doubling time. We therefore see “transmission dynamics” as a broader and, in our view, more accurate description of the scope of this work.

References

[1] Dreifuss, David, et al. "Estimated transmission dynamics of SARS-CoV-2 variants from wastewater are unbiased and robust to differential shedding." *Nature Communications* 16.1 (2025): 7456.

[2] Shah, Shimoni, et al. "Wastewater surveillance to infer COVID-19 transmission: A systematic review." *Science of The Total Environment* 804 (2022): 150060.

[3] Deshpande, Jagadish M., Sushmitha J. Shetty, and Zaeem A. Siddiqui. "Environmental surveillance system to track wild poliovirus transmission." *Applied and environmental microbiology* 69.5 (2003): 2919-2927.

[4] Nadeau, Sarah, et al. "Influenza transmission dynamics quantified from RNA in wastewater in Switzerland." *Swiss medical weekly* 154 (2024): 3503.

[5] Phan, Tin, et al. "Post-recovery viral shedding shapes wastewater-based epidemiological inferences." *Communications Medicine* 5.1 (2025): 193.

[6] Zheng, Xiawan, et al. "Wastewater surveillance provides spatiotemporal SARS-CoV-2 infection dynamics." *Engineering* 40 (2024): 70-77.
